# Supplementary material for: An Interactive Mapping and Case Discussion Seminar Introducing Medical Students to Climate Change, Environmental Justice, and Health
Source: MedEdPORTAL. 2024 Apr 16;20:11398. doi: 10.15766/mep_2374-8265.11398 (PMC11018717; doi:10.15766/mep_2374-8265.11398)
Supplement: Supplementary file 1 — Didactic Lectures.pptxSmall-Group Student Handouts Example.docxSmall-Group Debriefs Example.pptxPre- and Postseminar Surveys.docxQuiz.docx [file mep_2374-8265.11398-s001.zip › E. Quiz.docx]

**Appendix E - Quiz**

**Climate Change**

***Instructions:*** *This quiz can be offered electronically as a part of the educational management system. Submission of the quiz can be due one week after the session. Students can use course and outside resources to answer the questions (open content) and have one untimed attempt. A grading rubric is provided at the end of this document.*

Question 1

**1 pts**

Climate change can be defined as:

1. The state of the atmosphere at any given moment associated with average weather patterns
2. Short-term change in weather patterns
3. **Systematic change of the long-term state of the atmosphere**
4. Natural variability of regular patterns that occur over weeks to months

Question 2

**1 pts**

Human activities contributing to global warming include:

1. Extreme heat produced by solar electric panels
2. **Emission of greenhouse gasses from burning of fossil fuels and wood products**
3. Volcanic eruptions
4. Long residence times of carbon dioxide sinks

Question 3

**1 pts**

Climate change can affect health in both direct and indirect ways. Which of the following consequences of climate change can *directly*affect health?

1. **Degraded air quality**
2. Food and water scarcity
3. Increase in vector-born infectious diseases
4. Conflict displacement and forced migration

Question 4

**1 pts**

[Local site] is ranked the #XX USA city with respect to urban heat island intensity. Which of the following factors drive the urban heat island effect?

1. Excess ground storage of rainfall after large storms
2. **Prioritization of industry and transport in historically redlined neighborhoods over green space**
3. Paved surfaces that absorb less heat
4. Lack of resiliency efforts to prevent flooding in areas prone to flooding due to rising sea levels

Question 5

**5 pts**

Of the aspects of the comprehensive medical history presented in the lecture, which stood out to you as particularly important when considering the effect of climate change on a patient's health and why?

Question 6

**5 pts**

Based on the lecture, what are 5 domains of climate change mitigation that health care systems can implement to reduce their carbon footprint?

Answer: Decrease waste production, make energy use more efficient, decrease water use, improve transportation, upgrade supply chain to be more environmentally friendly

Question 7

**1 pts**

One strategy to reduce waste production by the health care industry includes which of the following?

1. Prohibit emergency vehicle idling outside of hospitals
2. Transition to renewable energy sources
3. **Redesign medical procedure kits to minimize waste**
4. Ask patients about their ability to recycle in their homes and neighborhoods

Question 8

**5 pts**

Of the topics discussed in the case scenarios, what stood out to you as particularly important or surprising and why?

**Total points: 20**

**Grading Rubric for essay questions**

Please take note of the proportion of the quiz final score that the question represents and 
ensure the effort used to generate the answer reflects this value. Students may receive either full, 
partial, or no credit for their answer. 
 Full credit 
 Partial credit – score will reflect less than the full value of the question’s worth 
 No credit (zero score) 
 
Full credit: Answer demonstrates thoughtful reflection, engagement with the course material and 
activities, and understanding of the topic 
 
Partial credit: While complete, the answer fails to demonstrate thoughtfulness, meaningful 
engagement with the materials, and/or full understanding of the topic 
 
No credit: The answer was incomplete, showed no evidence of meaningful reflection or 
thoughtfulness, or did not address the topic of the question 
 
Common pitfalls that will result in partial credit: 
 
1) The answer does not reflect the effort needed to fulfill full credit based on the weight of the 
question’s worth. Many short answer questions are worth 5 points, while multiple choice 
questions would be worth 1 point each. It is important to demonstrate the thoughtfulness and 
effort that will warrant such a high weight for one question. For example, it is difficult to 
demonstrate that you spent time and effort worth 5 points in just 2-3 sentences. 
 
2) While the length of the response is adequate, within the content of the response the ideas are 
repetitive, do not demonstrate any critical analysis of the course content, do not demonstrate 
self-reflection, or seem for the large part to copy the stem of the questions itself without 
adding any significantly new information. 
 
3) The response seems generic or reflective of broad themes and does not cite examples or 
reflect effort in engaging with the course materials/small group sessions.
