# Supplementary material for: An Interactive Mapping and Case Discussion Seminar Introducing Medical Students to Climate Change, Environmental Justice, and Health
Source: MedEdPORTAL. 2024 Apr 16;20:11398. doi: 10.15766/mep_2374-8265.11398 (PMC11018717; doi:10.15766/mep_2374-8265.11398)
Supplement: Supplementary file 1 — Didactic Lectures.pptxSmall-Group Student Handouts Example.docxSmall-Group Debriefs Example.pptxPre- and Postseminar Surveys.docxQuiz.docx [file mep_2374-8265.11398-s001.zip › B. Small-Group Student Handouts Example.docx]

**Appendix B: Small Group Activities**

**Student Handout Instructions and Examples**

This appendix provides student instructions for two breakout group activities: an interactive mapping exercise and a case studies activity. The mapping exercises explores how climate change affects the health of local populations. Examples provided highlight issues such as air pollution and asthma, urban heat islands due to redlining, and housing insecurity from flooding. The case studies illustrate how climate change presents in clinical settings and ways healthcare providers can tackle these issues. The examples provided address topics such as flooding’s impact on water quality and access to care, Lyme disease, and climate migrants.

The interactive mapping exercise should follow the “Climate Change and Population Health” lecture (Appendix A). The case studies activity follows the “ Climate Change and Health Systems” lecture (Appendix A). Each activity involves dividing the class into three groups (Group 1, Group 2, Group 3). Students are encouraged to form smaller nearby groups within their assigned group number to collaboratively answer the associated questions. They have 20 minutes to complete the activity before rejoining the larger group for discussion and sharing their findings (Appendix C).

| **Table of Contents** |  |
| --- | --- |
| Small Group Activity 1: Mapping Activity ............................................................................................ | 2 |
| Sources for Maps .................................................................................................................... | 2 |
| Reasoning for Selecting Specific Map Activities ...................................................................... | 3 |
| Example of Mapping Activities ................................................................................................ | 4 |
| Group 1: Air Pollution ................................................................................................ | 4 |
| Group 2: Redlining & Urban Heat Islands .................................................................. | 4 |
| Group 3: Flooding, Housing Insecurity ....................................................................... | 5 |
| Small Group Activity 2: Case Studies Activity ..................................................................................... | 6 |
| Reasoning for Selecting Specific Case Activities ..................................................................... | 6 |
| Example of Case Activities ....................................................................................................... | 7 |
| Group 1: Flooding, contaminated water, access to care ........................................... | 7 |
| Group 2: Urban Heat Islands ...................................................................................... | 7 |
| Group 3: Food insecurity & Climate Migrants........................................................... | 7 |
| References .............................................................................................................................. | 8 |

Small Group Activity 1: Mapping Activity

**Sources for Maps:**

This small group activity is focused on a mapping activity looking at climate change in the local context. There are sources publicly available that focus on specific aspects of the climate crisis including, but not limited to, urban heat island effects, flooding, etc. To make this small group activity most relevant and specific to your local area, we have provided a list of sources below. Most data available is for larger cities in the East Coast and Midwest. It is important to note that while the digital map resources listed below are publicly available at the time of submission, there is always the possibility that these websites may update, change and/or not be available in the future.

1. Maps for climate change in the local context.
   1. **Environmental Resource Inventory (ERI):** Reach out to your city’s Environmental Commission and ask if they have an Environmental Resource Inventory (ERI), report, or information related to the changing climate and how it is affecting the health of communities in your local area.
2. Maps for Redlining
   1. **“Redlining and Exposure to Urban Heat Islands Map '':** This interactive web map looks at USA neighborhoods and provides information on Home Owners Loan Corporation (HOLC) grades, average land surface temperature, tree coverage percentage, impervious surface percentage, and demographic information.
   2. **The Digital Scholarship Lab, “Mapping Inequality: Redlining in New Deal America,”.** Digital copy of original county and city maps made by the Federal Home Loan Bank Board and the HOLC with information on their HOLC grade.
3. Maps for Flooding
   1. **Neighborhoods at Risk:** These maps illustrate demographics at risk of experiencing extreme temperatures, coastal flooding, intense rainstorms, changed seasonal patterns, and different risk factors.
   2. **Climate Central.** Climate Central has an interactive coastal risk screening tool allowing users to explore sea level rise and coastal flood threats in a neighborhood by adjusting different controls (ex: year, sea-level projection source, pollution pathway, affordable housing, etc).
4. Maps for high temperature, flooding
   1. **The Climate Explorer:** Made by the National Oceanic and Atmospheric Administration, this online tool helps visualize past and projected future conditions regarding flooding and extreme temperatures. It also provides a list of top climate hazards for a selected location.

**Reasoning for Selecting Specific Map Activities:**

Map Activity #1: Air pollution and Asthma: Air pollution is associated with both the onset and exacerbation of asthma. Newark has a higher rate of adult asthma compared to the New Jerey state average.^1^ Students compare various maps to identify sources of air pollution and environmental risk factors that explain the distribution of asthma rates across different neighborhoods. Doing so allows students to conclude that although large healthcare systems aim to better the health of their communities, they may also be harming them.

Map Activity #2: Redlining and Urban Heat Islands (UHI): Across the USA, redlining led to the industrialization of many historically racially marginalized neighborhoods, contributing to the (UHI) effect today. ^2^ Newark, New Jersey ranks second in USA cities with the highest UHI intensity,^3^ and as a state, New Jersey is warming twice as fast as the average for the lower 48 states.^4^ As temperatures rise, the UHI increases, disproportionately exposing certain populations to heat-related illnesses.^5^ Maps are used to illustrate the relationship between spatial patterns of UHI and historically redlined neighborhoods. Understanding the effects of extreme heat on populations is essential to medical education, as many students will go on to complete residency training at large academic medical institutions, many of which were built in and serve populations in historically redlined areas.

Map Activity #3: Flooding and Housing Insecurity: New Jersey has the largest number of affordable-housing units exposed to sea-level rise amongst coastal states.^6^ The amount of affordable housing units vulnerable to coastal flooding can increase to more than four times by 2050.^6,7^ This is particularly concerning for Newark residents, as 26.5% of the city of Newark is already a flood zone.^8^ Maps are used to identify which neighborhoods and populations were most at risk of housing insecurity due to the flooding. By completing the activity, students understand how increased risk of flooding events and low socioeconomic status threaten housing security of socially disadvantaged communities and their health.

**Examples of Mapping Activities**

**Group 1: Air Pollution**

Increased air temperature from climate change, the burning of fossil fuels and the continued emission of greenhouse gases worsens air quality. This in turn leads to increase pollen levels as well as particulate matter and ground level ozone pollution, exacerbating respiratory illnesses including asthma.^9^ Newark has higher rate of adult asthma (12.9%) compared to the New Jersey state average (9%).^8^

1. Certain wards in Newark are more affected by air pollution than others. Using the Newark Environmental Resource Inventory, compare the “Ground-level Ozone” and “Asthma Rate in Adults by Census Tract” maps. ^8^ Which wards have the highest level of adult asthma? What environmental conditions might influence asthma rates/severity in those wards?
2. In Newark, University Hospital is a major sources of air pollution (see the ‘Air Quality Permitted Facilities’ map), ^8^ and thus are thought to be a contributor to the above average asthma rates seen in Newark. Other than asthma, what other diseases can be caused by, exacerbated and/or associated with air pollution?

**Group 2: Redlining & Urban Heat Islands**

The location of urban heat islands in Newark have been attributed to redlining by the Home Owners Loan Corporation in the 1930s.^3,8,10^ With time, these wards became less monetarily valuable. Businesses then had the opportunity to acquire and develop these areas into heavily industrialized zones, where infrastructure and transportation were prioritized. Waterways were utilized as industrial dumping sites, and infrastructure was characterized by multiple lanes of traffic and narrow sidewalks. Today, in the presence of excessive heat, this results in urban heat islands in historically red-lined zones.^3^

1. Using the Redlining and Exposure to Urban Heat Islands Map, ^10^ search “Essex Country, NJ” and locate University Hospital. This area received a Home Owners’ Loan Corporation grade of D (Hazardous). What neighborhood characteristics have contributed to this area’s temperature difference (+3℃) compared to the citywide average?
2. Living in urban heat islands can affect fluid balance in the body. Taking what you know from previous organ system blocks, what types of medications affecting fluid balance put patients at an increased risk for heat stroke during a heat wave?

**Group 3: Flooding, Housing Insecurity**

Newark’s elevation ranges from sea level in the East Ward to about 260 feet along the City’s western boundary.^8^ Because of the low elevation, relatively flat topography, proximity to waterways, impervious surfaces, and limited vegetation, about 55.2% of the East Ward and 26.5% of the city of Newark is currently a flood zone.^8^

1. Using the Climate Central: Land Projected Flood Level map, ^7^ investigate Newark’s flood risk between the years 2030-2100 under different scenarios using the settings key. Which areas of Newark are most at risk for flooding by 2050 if we continue on our current trajectory?
2. Using the Newark Environmental Resource Inventory, compare the “Climate Exposure: Sea, Lake and Overland Surges from Hurricanes (SLOSH) Category 1 Flood Zones” and “Affordable Housing” maps,^8^ which types of housing units are currently at risk for flooding? What other populations are also at risk for housing insecurity due to flooding?

**Small Group Activity 2: Case Studies Activity**

**Reasoning for Selecting Specific Case Activities:**

Case Activity #1: Flooding, contaminated water, access to care: This case study was inspired by real events that occurred when Hurricane Ida hit the Ironbound section of Newark, New Jersey.^11^ It looks at access to water and increased risk of infection when flooding occurs.

Case Activity #2: Vector Borne Illnesses- Lyme Disease: According to the Environmental Protection Agency, Lyme Disease is a climate change indicator,^12^ and New Jersey has one of the highest rates of cases in the USA.^13^ As the climate crisis continues, the number of cases is expected to increase.^12^ This case was designed to emphasize protective measures against Lyme disease and how to recognize symptoms outside of the traditional season.

Case Activity #3: Food Insecurity and Climate Migrants: This case study was inspired by events that occurred when Tropical Storm Grace hit Haiti, resulting in many Haitian individuals seeking refuge in the USA.^14^ Historically, the New York-Newark-Jersey City Metropolitan area is among the top five areas in regards to concentration of Haitian immigrants.^15^ With an increase in extreme weather events, we anticipate an influx of Haitian climate migrants in Newark. This case study illustrated how climate change and extreme weather events affect ability to seek, access, and provide care.

**Examples of Case Activities**

**Group 1: Flooding, contaminated water, access to care**

In September 2021 torrential downpour from Hurricane Ida caused storm surges, which led to significant flooding in Clarissa’s apartment.^11^ She comes in complaining of an infected wound she acquired during the flood and you diagnose her with *Staphylococcus* *aureus* skin and soft tissue infection, a common flood-related bacterial skin infection from traumatic wounds.^16^ Clarissa also wants to know if it’s safe to use tap water since it's been days since the storm occurred.

1. What counseling would you provide patients about drinking water and water-borne illnesses during floods and/or storm surges?
2. ‘Urban flooding’ is flooding that occurs when heavy rainfall overwhelms the local stormwater drainage capacity, a frequent problem in multiple neighborhoods across the city. How can flooding impact a patient’s ability to access healthcare? How can it impact healthcare workers/staff/students’ ability to come into the hospital and provide care?

**Group 2: Vector Borne Illnesses- Lyme Disease**

It’s mid-November and a 55-year-old patient comes in with fever, fatigue, joint aches, and a target sign rash. She mentions she went hiking in Northern New Jersey near the New York border last week to enjoy the last bit of the fall foliage. Although out of season, you diagnose her with Lyme disease.

1. New Jersey ranks third nationally in cases of Lyme Disease, with tick season occurring from April through October. ^12,13^ How has climate change has contributed to tick survival, and thus made it possible to acquire Lyme Disease outside the normal tick season?
2. Each year, approximately 476,000 people in the USA are diagnosed with Lyme disease.^13^ How would you educate your patients about the different ways to protect themselves from Lyme Disease?

**Group 3: Food insecurity & Climate Migrants**

Haiti made the news in 2021. In July a presidential assassination was followed by an earthquake and hurricane in August.^14^ Esther and her family were originally farmers in Haiti and their diet was heavily plant based. Already suffering from low crop yields, the aftermath of Tropical Storm Grace left them with no harvest and limited food. This coupled with government instability, poverty, violence, and an ongoing COVID-19 pandemic has resulted in thousands of climate migrants. Now she and her family have fled to the US-Mexican border.

1. Increased CO2 levels have been shown to cause plants to grow faster, but they may contain a lower content of N, K, P, Ca, S, Mg, Fe, Zn, and Cu.^17^ Thus, populations that have traditionally eaten a heavy plant-based diet are at risk for which diseases/illnesses? Which individuals in society are most vulnerable to micronutrient deficiencies?
2. What public health concerns may come up with mass migrations? Access to care?
3. Although developing countries have contributed the least to greenhouse gas emissions and climate change, they are already feeling its impact. Without the resources to adapt, this increases the potential for instability due to struggles over natural resources, food security, and financial uncertainty, leading to increased war and/or mass migrations.^18^ How can the US healthcare system prepare for climate migrants now and in the future?

**References**

1. Newark Environmental Commission. *City of Newark Environmental Resource Inventory.* 2021. Accessed September 7, 2021. <https://data-newgin.opendata.arcgis.com/pages/eri>
2. Hoffman JS, Shandas V, Pendleton N. The Effects of Historical Housing Policies on Resident Exposure to Intra-Urban Heat: A Study of 108 US Urban Areas. *Climate*. 2020;8(1):12. doi:10.3390/cli8010012
3. Climate Central. *Hot Zones: Urban Heat Islands*. 2021. Accessed July 7, 2021. <https://assets.ctfassets.net/cxgxgstp8r5d/1XZZjkLYwtcmKL5k3wEinl/5f8c9b5b2d8dd56e1bda7f51278fc3d2/2021_UHI_Report.pdf>
4. State Climate Summaries, New Jersey. NOAA National Centers for Environmental Information. 2019. Accessed December 4, 2021. <https://statesummaries.ncics.org/chapter/nj/>
5. Luber G, Lemery J. *Global Climate Change and Human Health: From Science to Practice.* 2020.
6. Buchanan MK, Kulp S, Cushing L, Morello-Frosch R, Nedwick T, Strauss B. Sea level rise and coastal flooding threaten affordable housing. *Environ. Res. Lett.* 2020;15(12):124020. doi:10.1088/1748-9326/abb266
7. Land Projected to be Below 10-Year Flood Zone by 2050 Map. Climate Central. Published 2020. Accessed December 11, 2021. <https://coastal.climatecentral.org/map/12/-73.9605/40.7101/?theme=sea_level_rise&map_type=year&basemap=roadmap&contiguous=true&elevation_model=best_available&forecast_year=2050&pathway=rcp45&percentile=p50&refresh=true&return_level=return_level_1&rl_model=tebaldi_2012&slr_model=kopp_2014>
8. Newark Environmental Commission. *City of Newark Environmental Resource Inventory.* 2021. Accessed September 7, 2021. <https://data-newgin.opendata.arcgis.com/pages/eri>
9. Orru H, Ebi KL, Forsberg B. The Interplay of Climate Change and Air Pollution on Health. *Curr Environ Health Rep*. 2017;4(4):504-513. doi: 10.1007/s40572-017-0168-6
10. Science Museum of Virginia, Esri. Redlining and Exposure to Urban Heat Islands Map. ArcGIS Living Atlas of the World. Published 2020. Accessed December 11, 2021. <https://www.arcgis.com/apps/dashboards/73e329457b6644e7aeff13ecce43c8d8>
11. Bonamo M. As Ironbound Recovers From Ida Residents Look to Long Term Flooding Solutions. *TAP into Newark*. Published September 10, 2021. Accessed December 8, 2021. <https://www.tapinto.net/towns/newark/sections/east-ward/articles/as-ironbound-recovers-from-ida-residents-look-to-long-term-flooding-solutions>
12. Climate Change Indicators: Lyme Disease. United States Environmental Protection Agency. Published 2021. Accessed January 9, 2021. <https://www.epa.gov/climate-indicators/climate-change-indicators-lyme-disease>
13. Lyme Disease Data and Surveillance. Centers for Disease Control and Prevention. Updated 2019. Accessed July 10, 2021. <https://www.cdc.gov/lyme/datasurveillance/index.html>
14. Ramirez R. Climate change is intensifying the US border crisis. it will only get worse. *CNN*. Published September 26, 2021. Accessed October 9, 2021. <https://www.cnn.com/2021/09/26/us/climate-change-migration-border-haiti/index.html>
15. Olsen-Medina K, Batalova J. Haitian Immigrants in the United States. Migration Policy Institute. Published August 12, 2020. Accessed April 10, 2021. <https://www.migrationpolicy.org/article/haitian-immigrants-united-states>
16. Tempark T, Lueangarun S, Chatproedprai S, Wananukul S. Flood-related skin diseases: a literature review. *Int J Dermatol*. 2013;52(10):1168-1176. doi:10.1111/ijd.12064
17. Semba RD, Askari S, Gibson S, Bloem MW, Kraemer K. The Potential Impact of Climate Change on the Micronutrient-Rich Food Supply. *Adv Nutr*. 2022;13(1):80-100. doi:10.1093/advances/nmab104
18. The Office of the Director of National Intelligence. *Climate Change and International Responses Increasing Challenges to US National Security Through 2040.* 2021. NIC-NIE-2021-10030-A. Accessed November 14, 2021. <https://www.dni.gov/index.php/newsroom/reports-publications/reports-publications-2021/3575-national-intelligence-estimate-on-climate-change>
