# Supplementary material for: An Interactive Mapping and Case Discussion Seminar Introducing Medical Students to Climate Change, Environmental Justice, and Health
Source: MedEdPORTAL. 2024 Apr 16;20:11398. doi: 10.15766/mep_2374-8265.11398 (PMC11018717; doi:10.15766/mep_2374-8265.11398)
Supplement: Supplementary file 1 — Didactic Lectures.pptxSmall-Group Student Handouts Example.docxSmall-Group Debriefs Example.pptxPre- and Postseminar Surveys.docxQuiz.docx [file mep_2374-8265.11398-s001.zip › C. Small-Group Debriefs Example.pptx]

## Slide 1
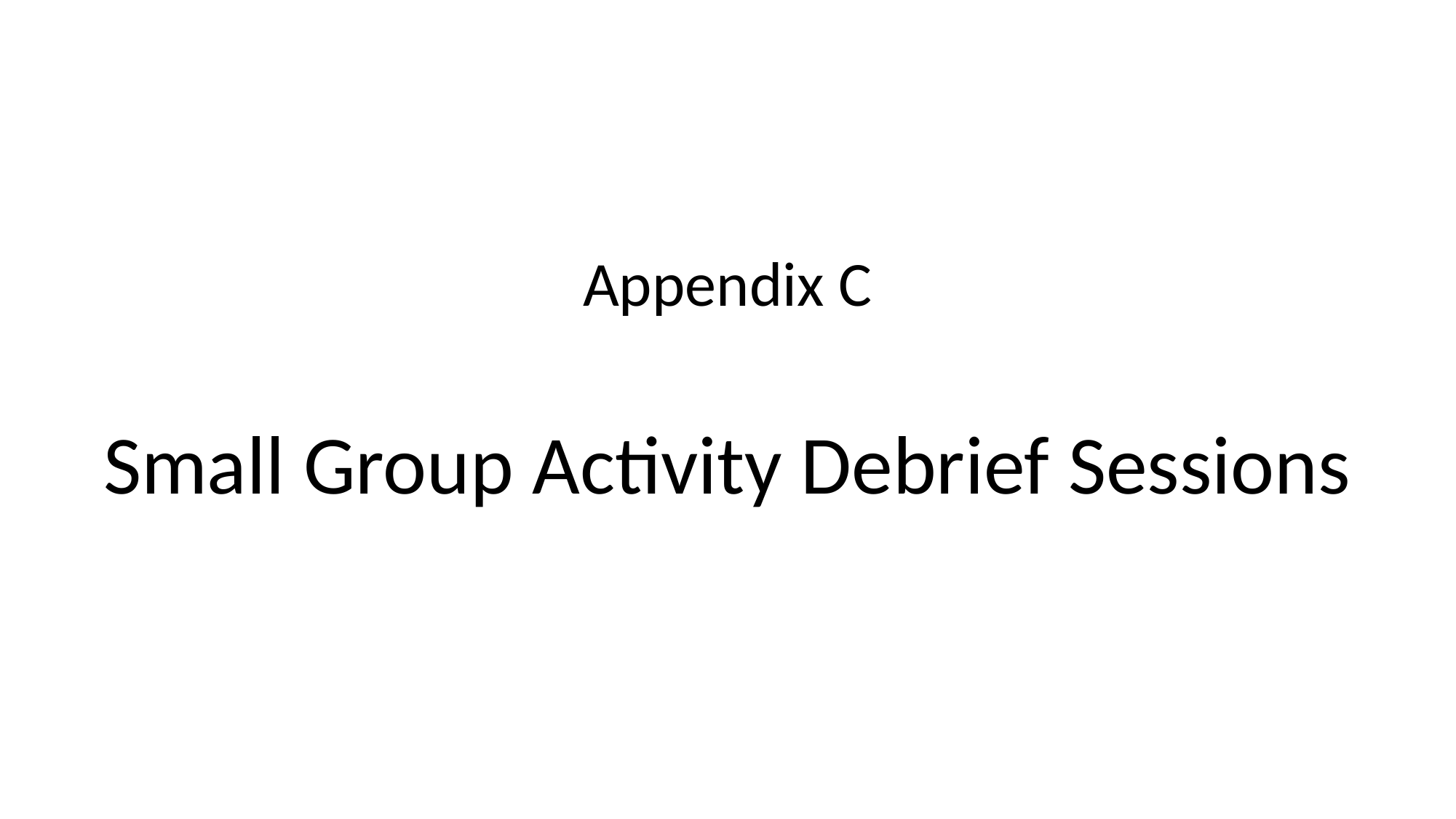

# Appendix C
Small Group Activity Debrief Sessions

## Slide 2
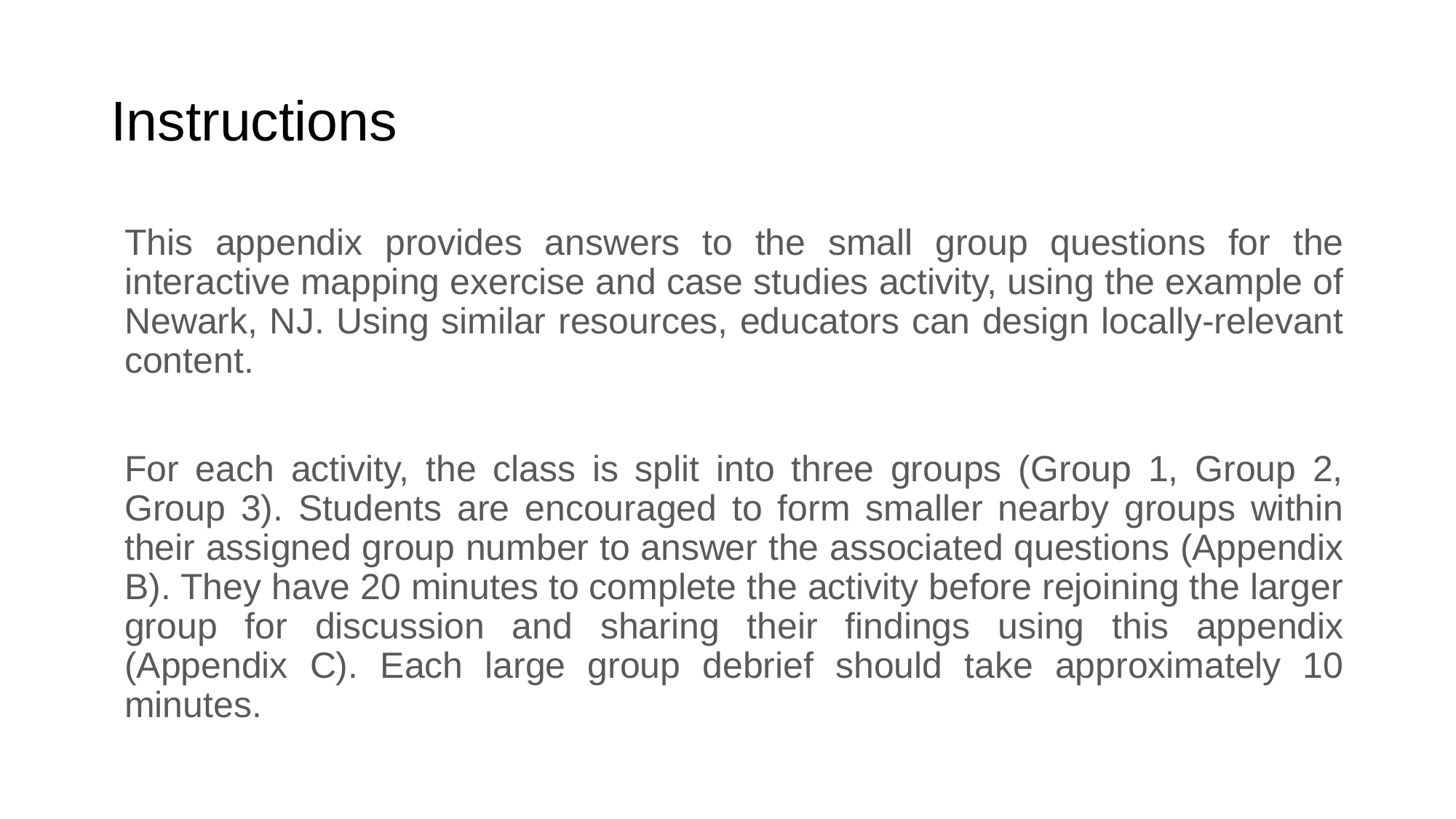

# Instructions
This appendix provides answers to the small group questions for the interactive mapping exercise and case studies activity, using the example of Newark, NJ. Using similar resources, educators can design locally-relevant content.
For each activity, the class is split into three groups (Group 1, Group 2, Group 3). Students are encouraged to form smaller nearby groups within their assigned group number to answer the associated questions (Appendix B). They have 20 minutes to complete the activity before rejoining the larger group for discussion and sharing their findings using this appendix (Appendix C). Each large group debrief should take approximately 10 minutes.

## Slide 3
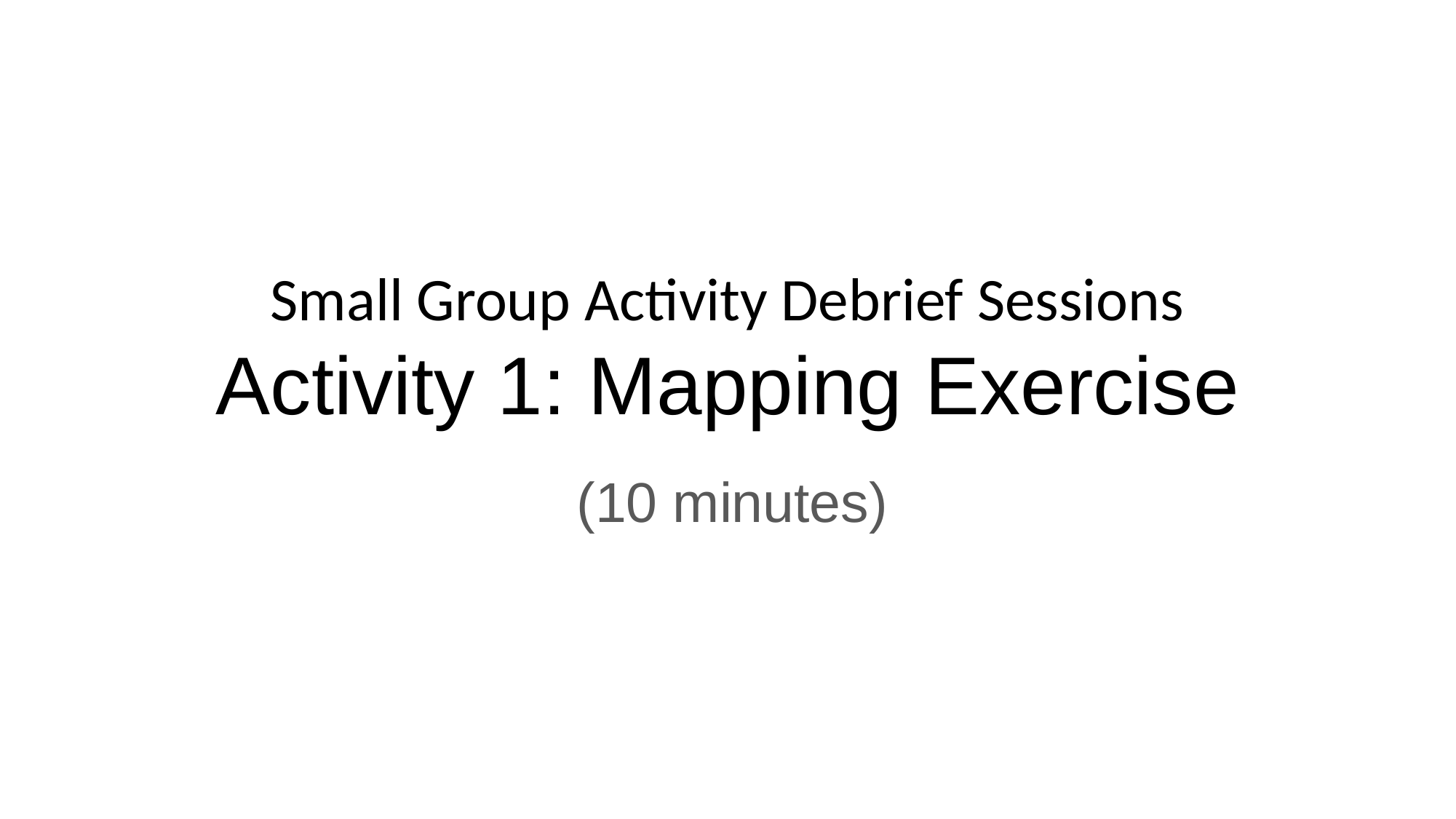

# Small Group Activity Debrief Sessions Activity 1: Mapping Exercise
(10 minutes)

## Slide 4
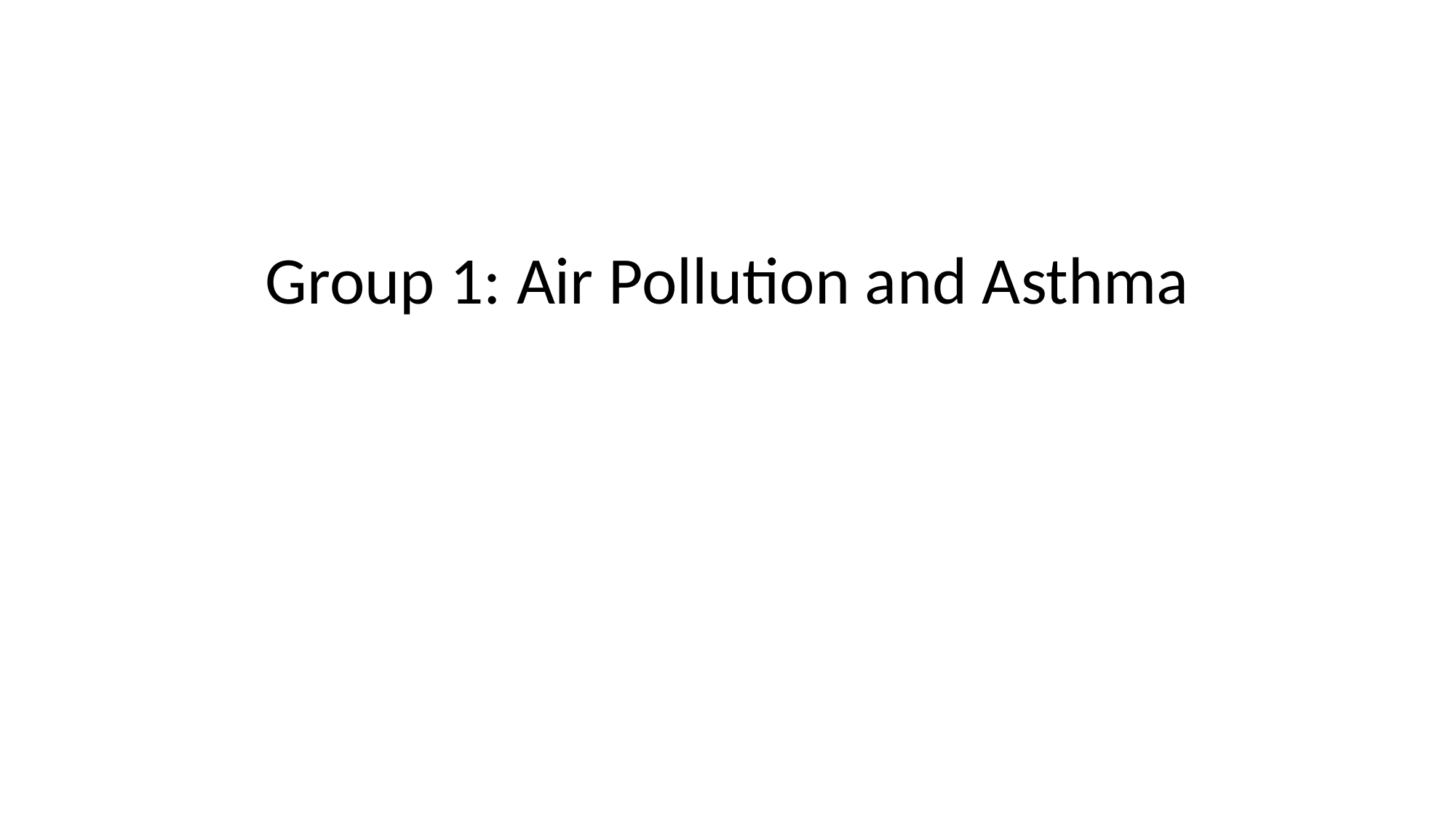

# Group 1: Air Pollution and Asthma

## Slide 5
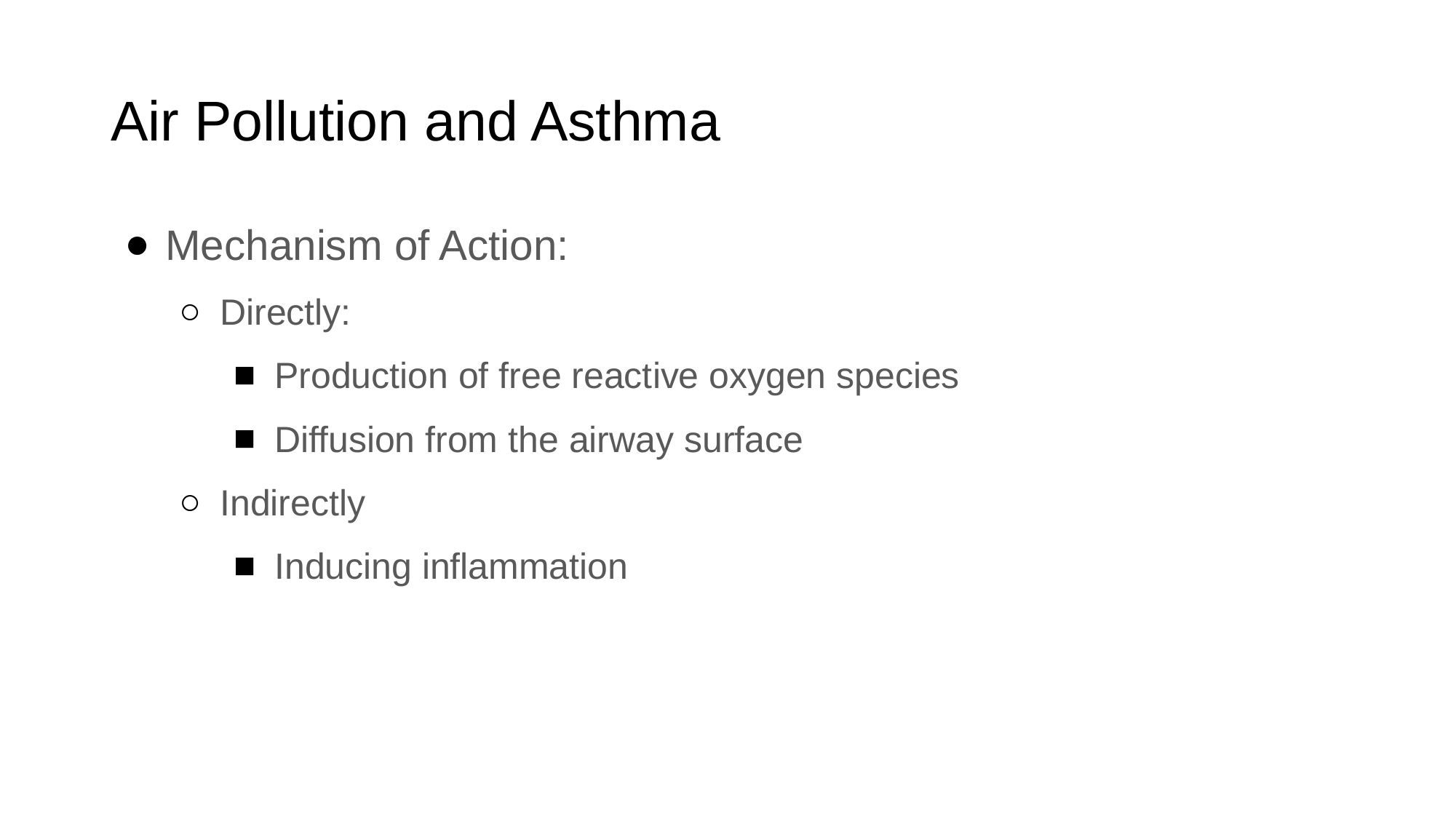

# Air Pollution and Asthma
Mechanism of Action:
Directly:
Production of free reactive oxygen species
Diffusion from the airway surface
Indirectly
Inducing inflammation

## Slide 6
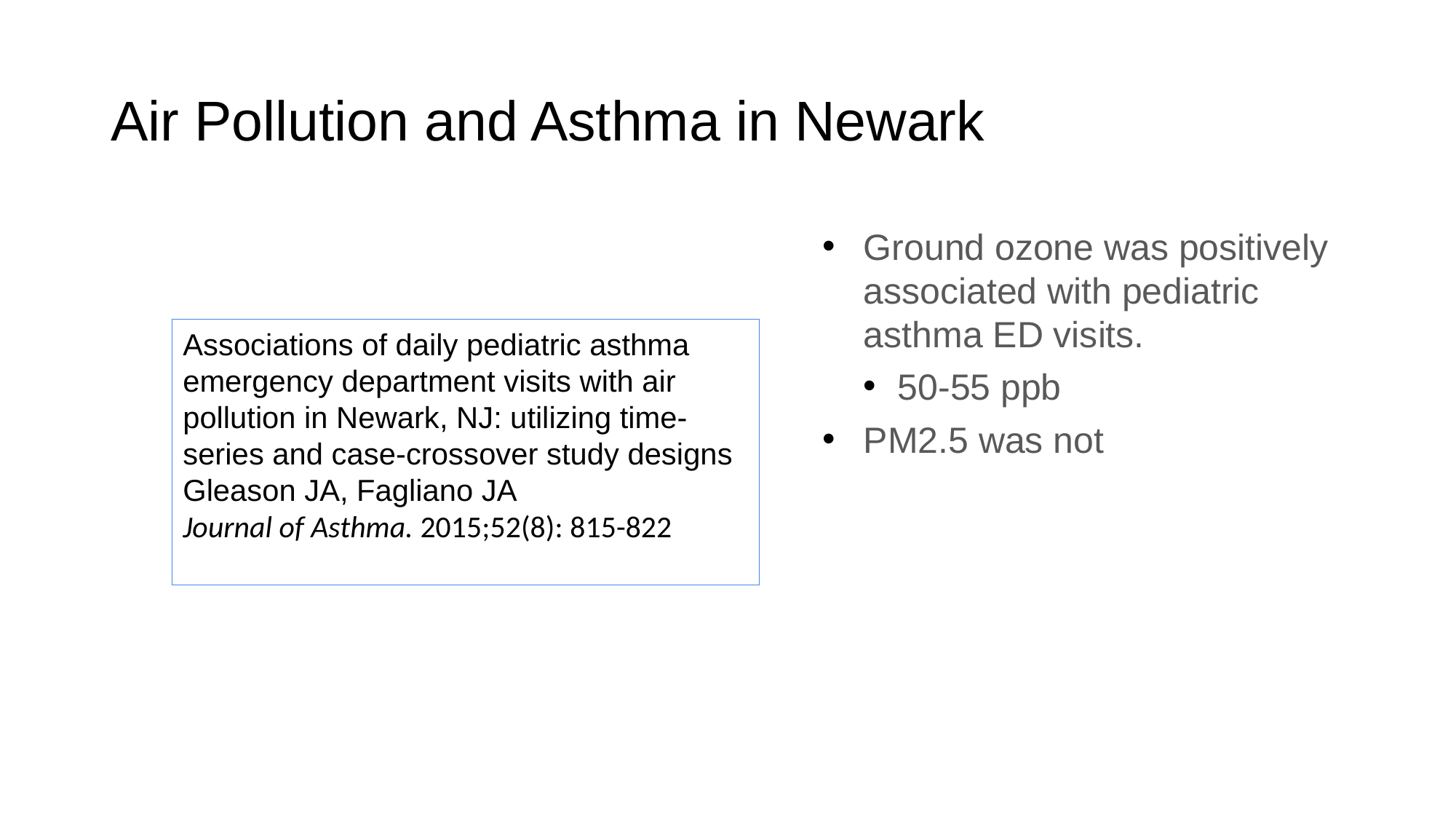

# Air Pollution and Asthma in Newark
Ground ozone was positively associated with pediatric asthma ED visits.
50-55 ppb
PM2.5 was not
Associations of daily pediatric asthma emergency department visits with air pollution in Newark, NJ: utilizing time-series and case-crossover study designs
Gleason JA, Fagliano JA
Journal of Asthma. 2015;52(8): 815-822

## Slide 7
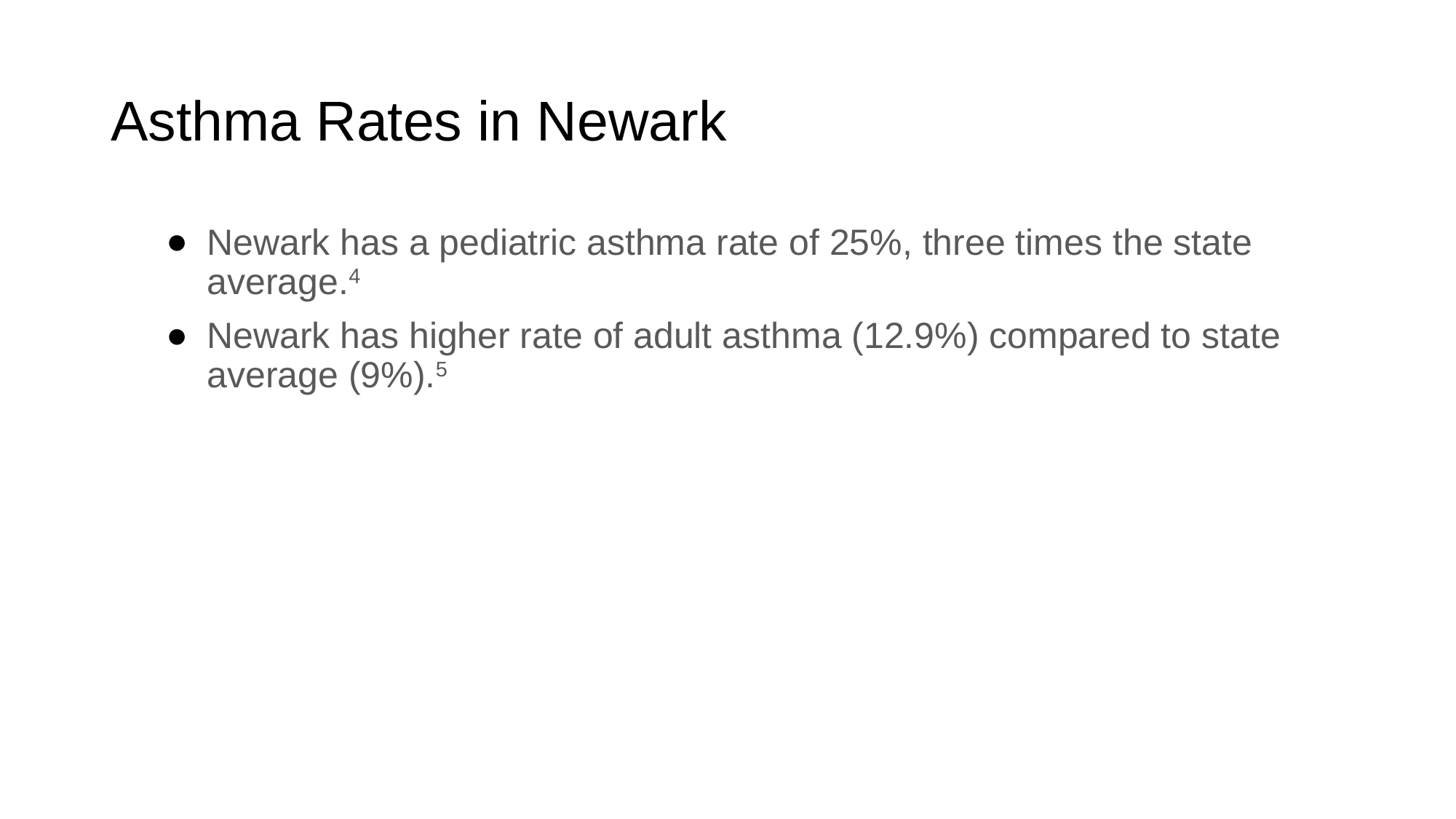

# Asthma Rates in Newark
Newark has a pediatric asthma rate of 25%, three times the state average.4
Newark has higher rate of adult asthma (12.9%) compared to state average (9%).5

## Slide 8
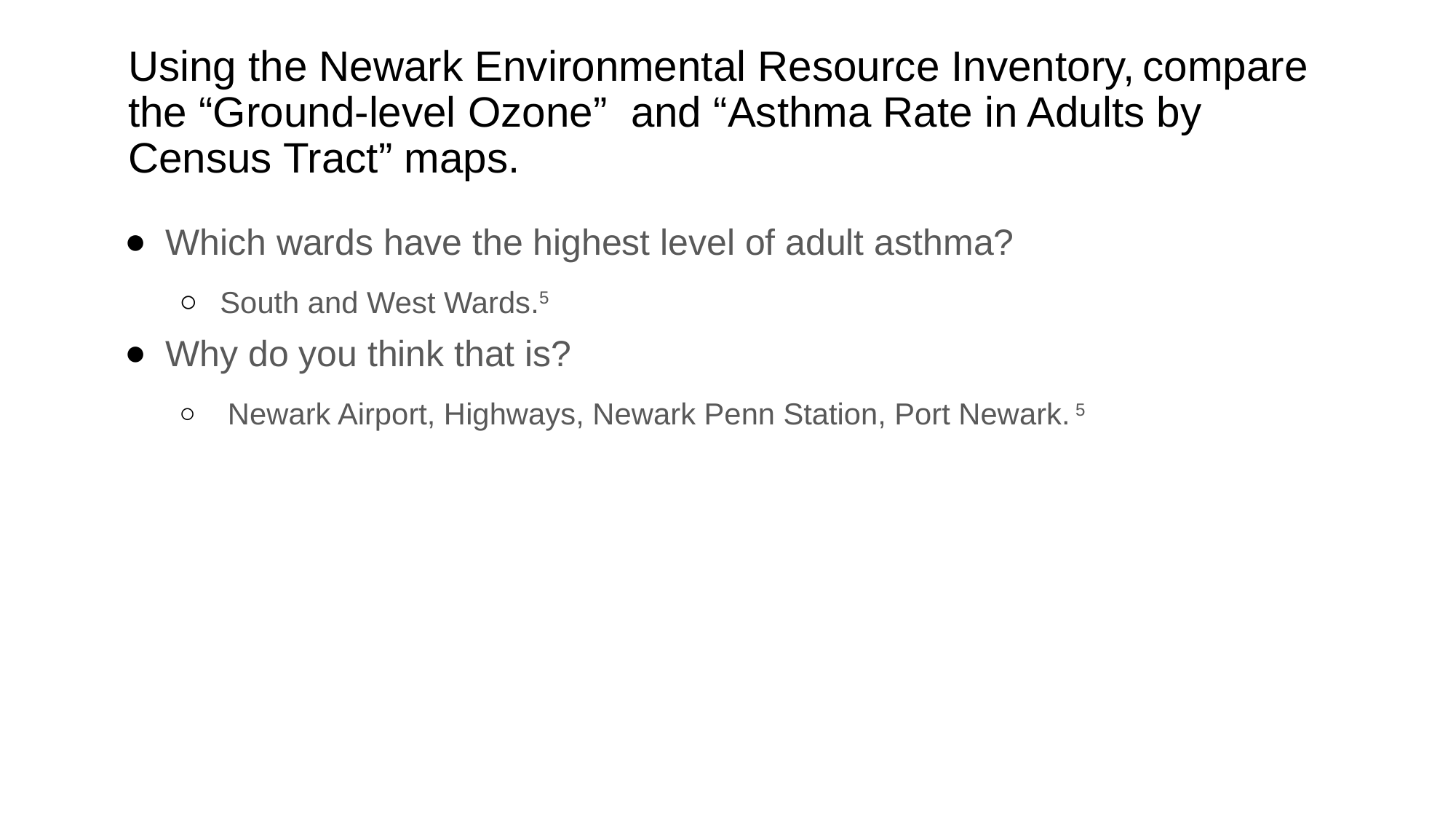

# Using the Newark Environmental Resource Inventory, compare the “Ground-level Ozone” and “Asthma Rate in Adults by Census Tract” maps.
Which wards have the highest level of adult asthma?
South and West Wards.5
Why do you think that is?
 Newark Airport, Highways, Newark Penn Station, Port Newark. 5

## Slide 9
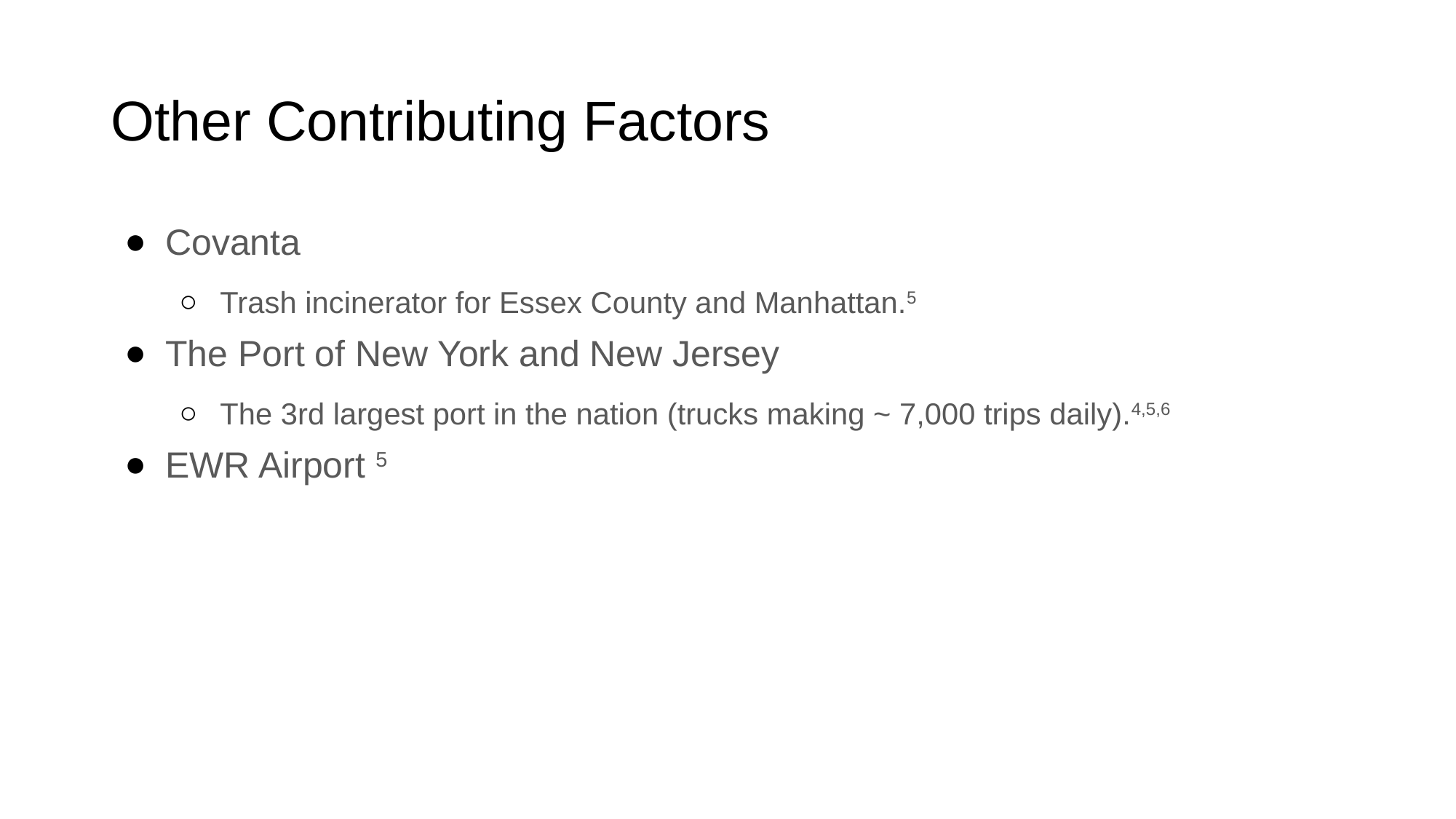

# Other Contributing Factors
Covanta
Trash incinerator for Essex County and Manhattan.5
The Port of New York and New Jersey
The 3rd largest port in the nation (trucks making ~ 7,000 trips daily).4,5,6
EWR Airport 5

## Slide 10
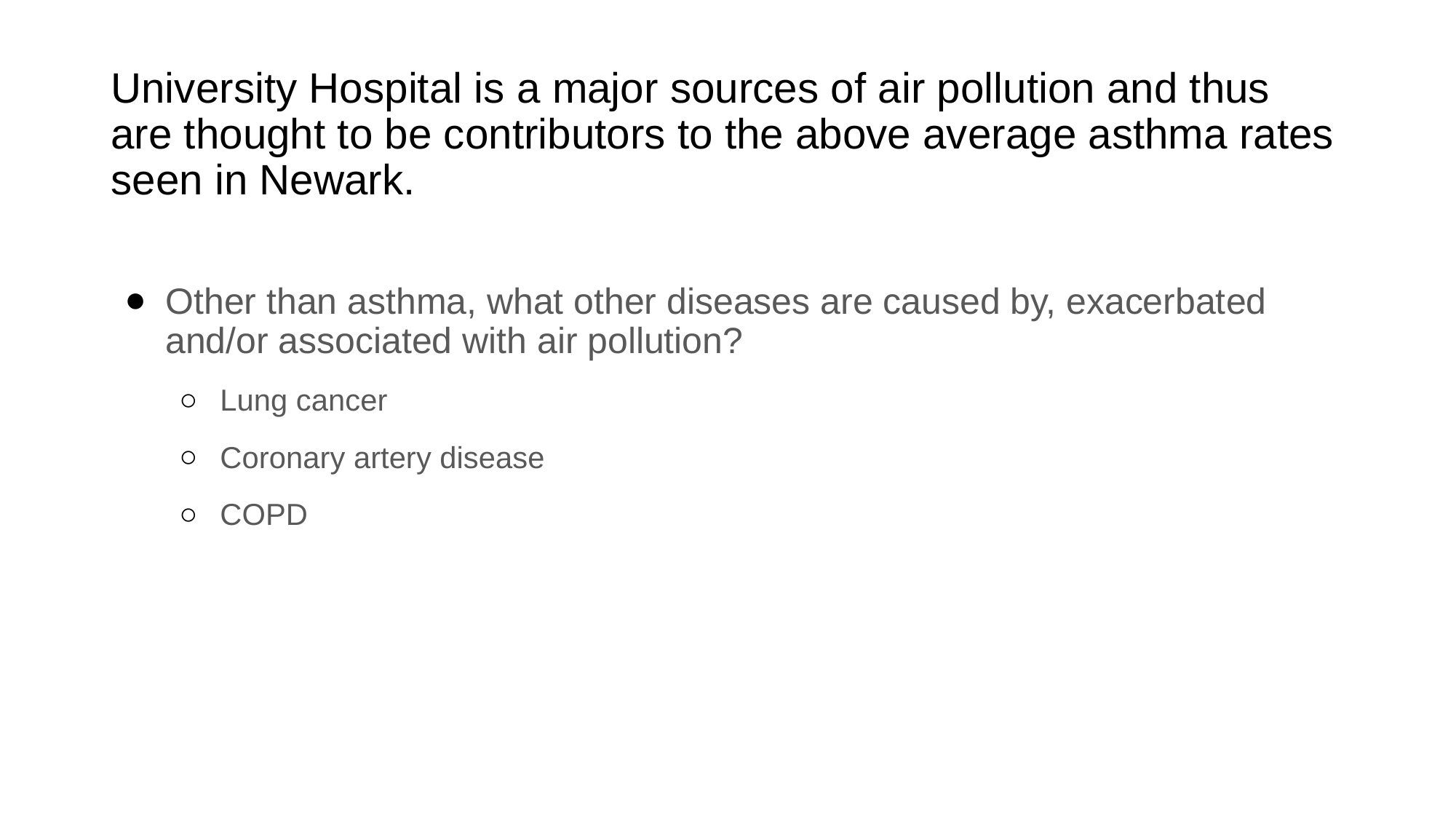

# University Hospital is a major sources of air pollution and thus are thought to be contributors to the above average asthma rates seen in Newark.
Other than asthma, what other diseases are caused by, exacerbated and/or associated with air pollution?
Lung cancer
Coronary artery disease
COPD

## Slide 11
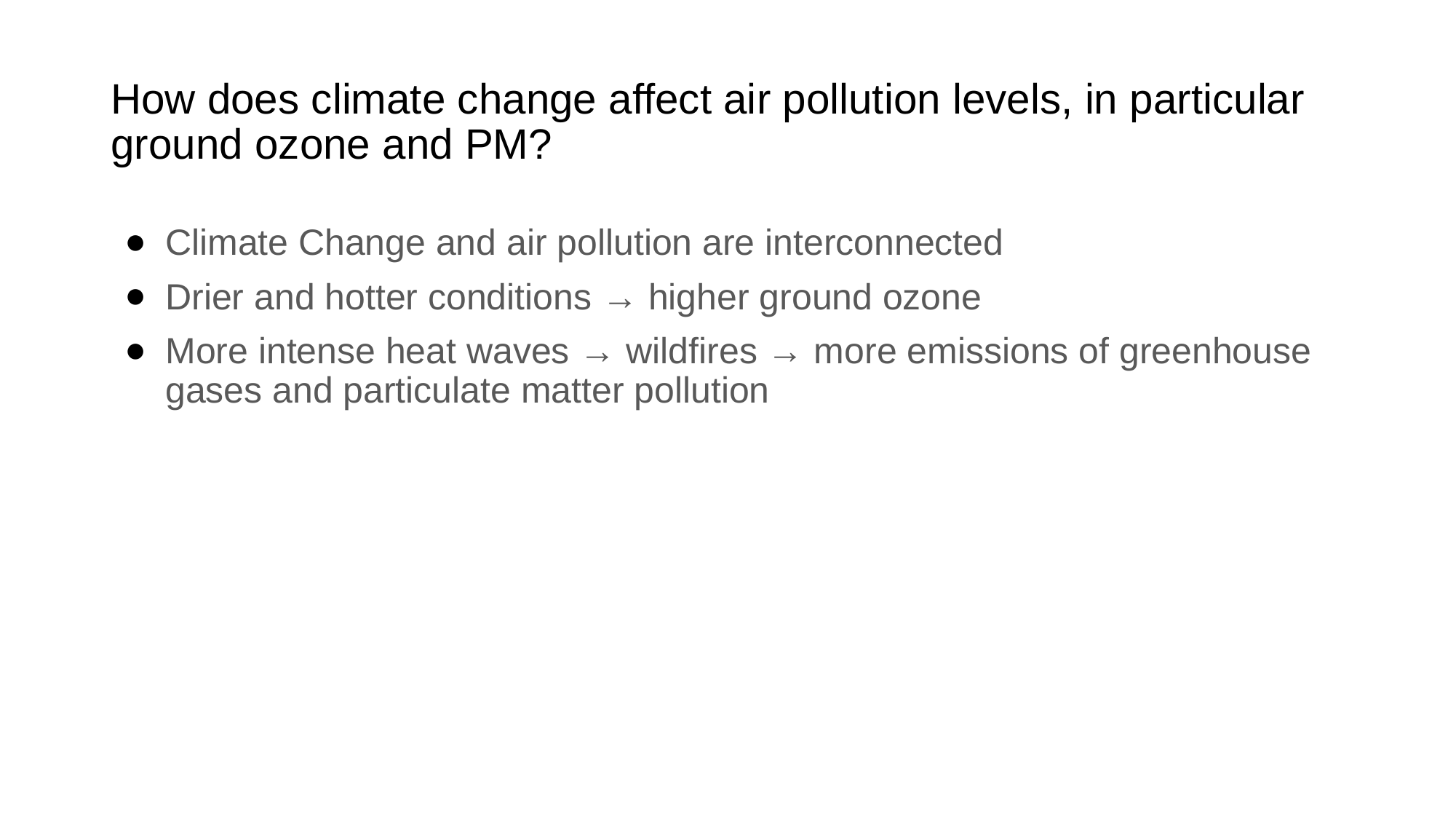

# How does climate change affect air pollution levels, in particular ground ozone and PM?
Climate Change and air pollution are interconnected
Drier and hotter conditions → higher ground ozone
More intense heat waves → wildfires → more emissions of greenhouse gases and particulate matter pollution

## Slide 12
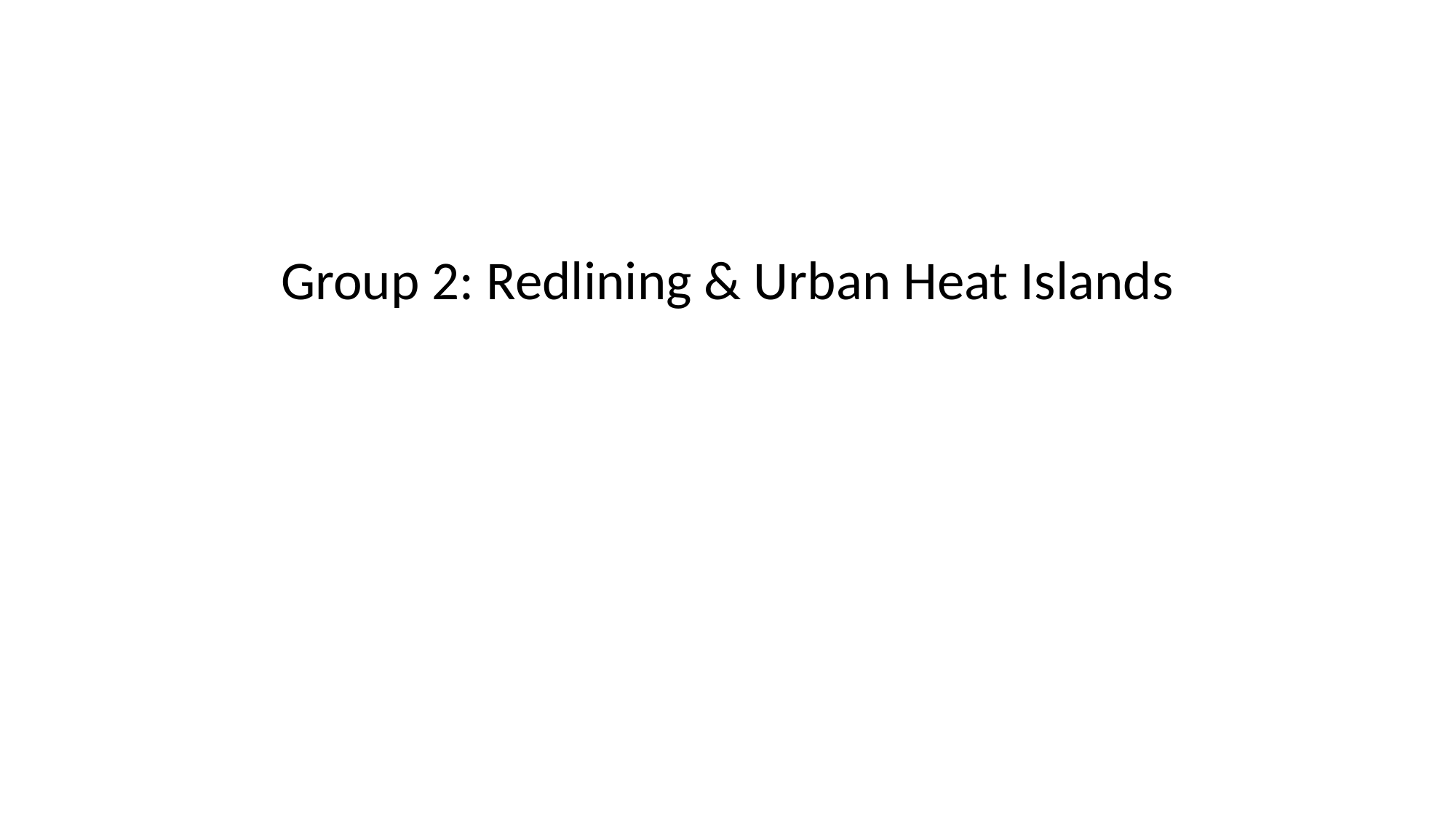

# Group 2: Redlining & Urban Heat Islands

## Slide 13
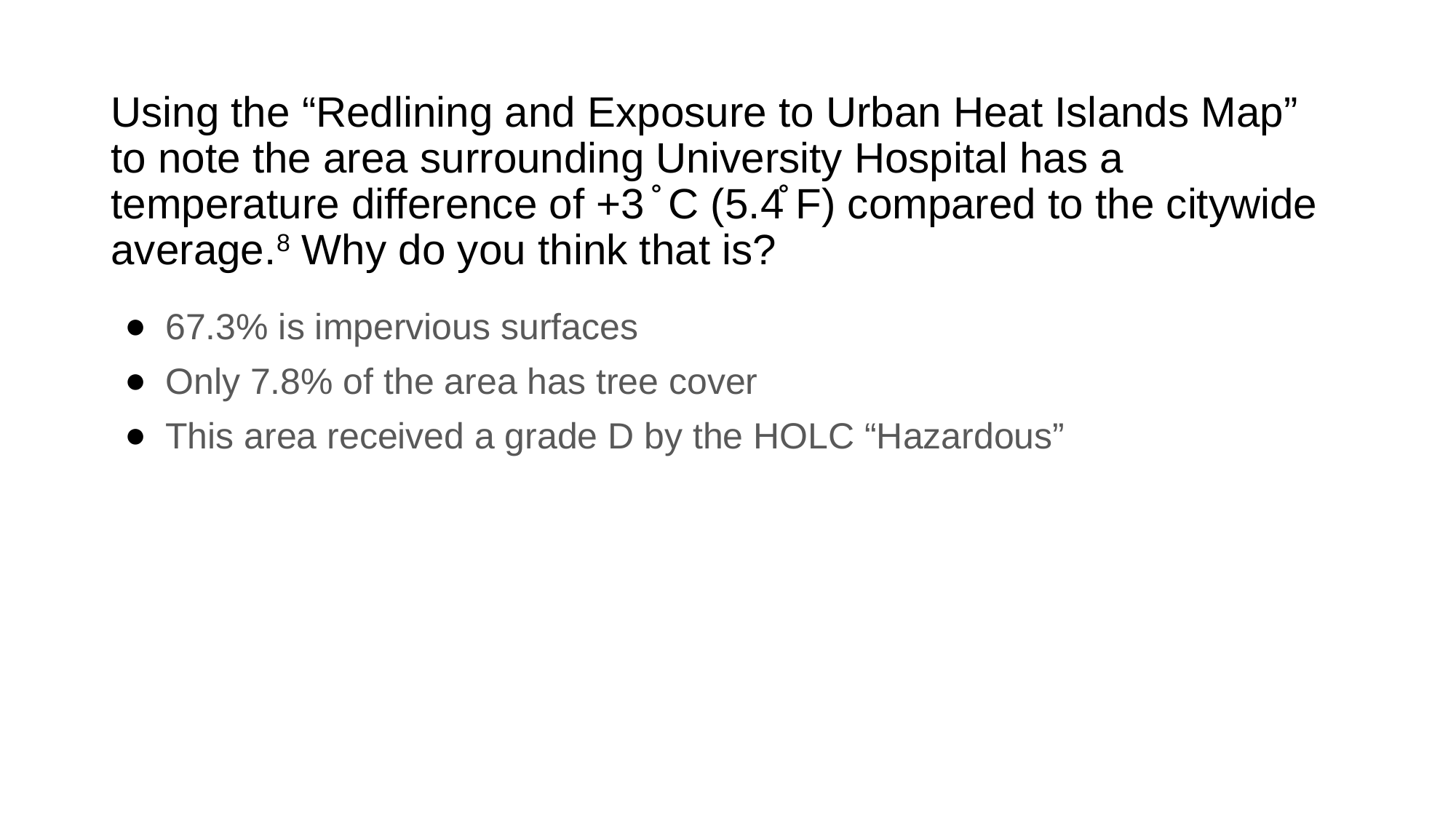

# Using the “Redlining and Exposure to Urban Heat Islands Map” to note the area surrounding University Hospital has a temperature difference of +3 ̊ C (5.4̊ F) compared to the citywide average.8 Why do you think that is?
67.3% is impervious surfaces
Only 7.8% of the area has tree cover
This area received a grade D by the HOLC “Hazardous”

## Slide 14
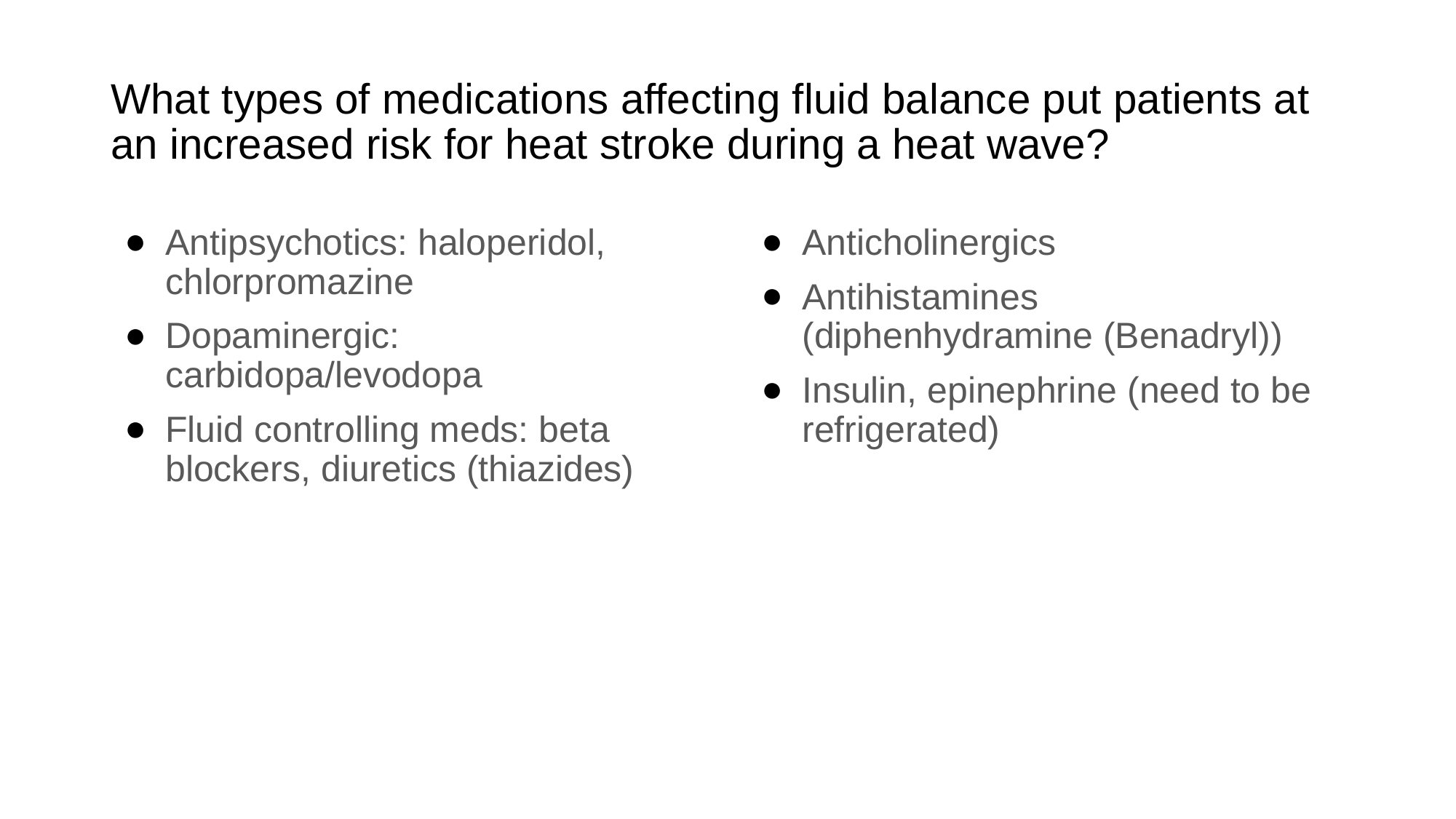

# What types of medications affecting fluid balance put patients at an increased risk for heat stroke during a heat wave?
Antipsychotics: haloperidol, chlorpromazine
Dopaminergic: carbidopa/levodopa
Fluid controlling meds: beta blockers, diuretics (thiazides)
Anticholinergics
Antihistamines (diphenhydramine (Benadryl))
Insulin, epinephrine (need to be refrigerated)

## Slide 15
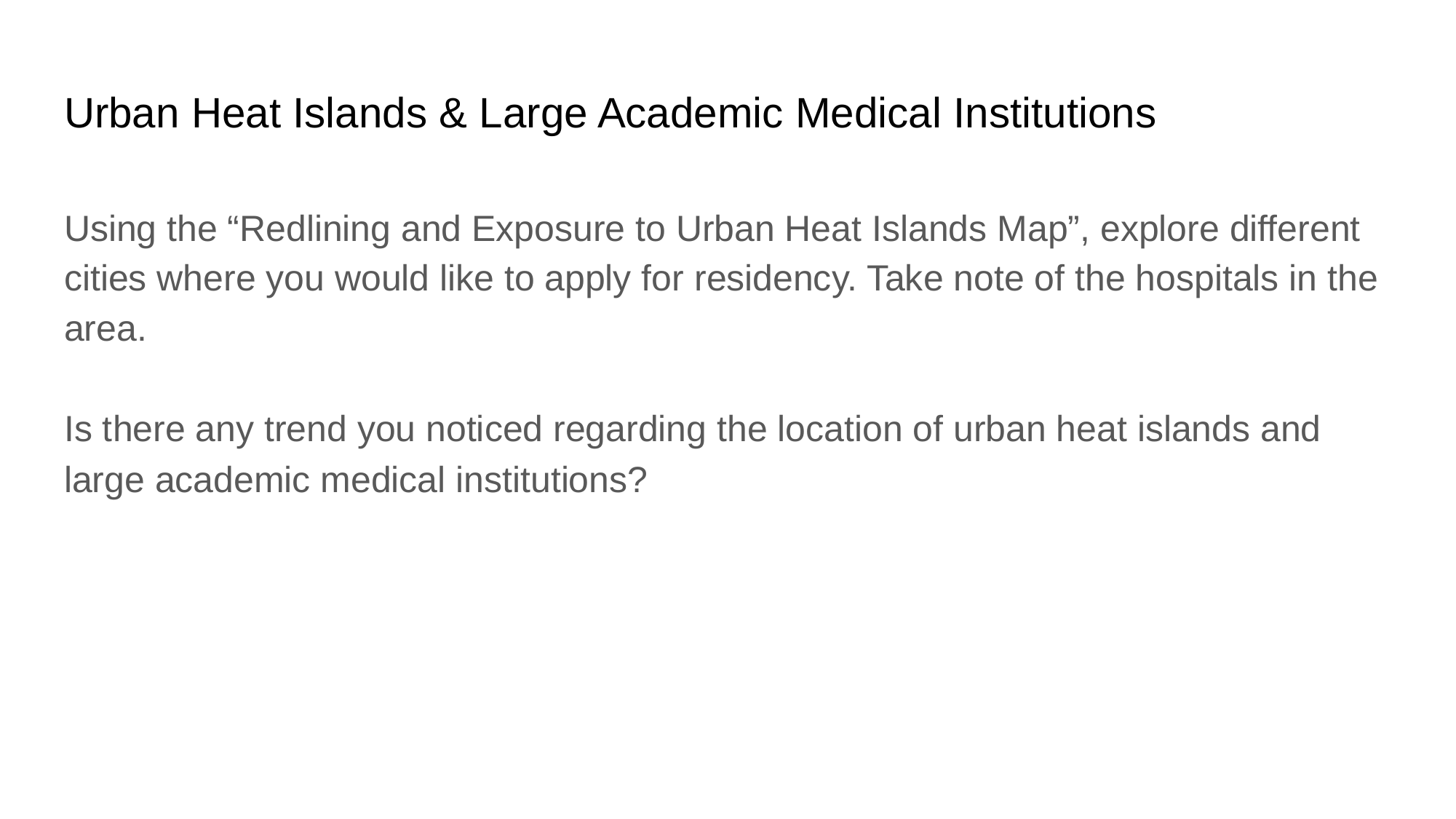

# Urban Heat Islands & Large Academic Medical Institutions
Using the “Redlining and Exposure to Urban Heat Islands Map”, explore different cities where you would like to apply for residency. Take note of the hospitals in the area.
Is there any trend you noticed regarding the location of urban heat islands and large academic medical institutions?

## Slide 16
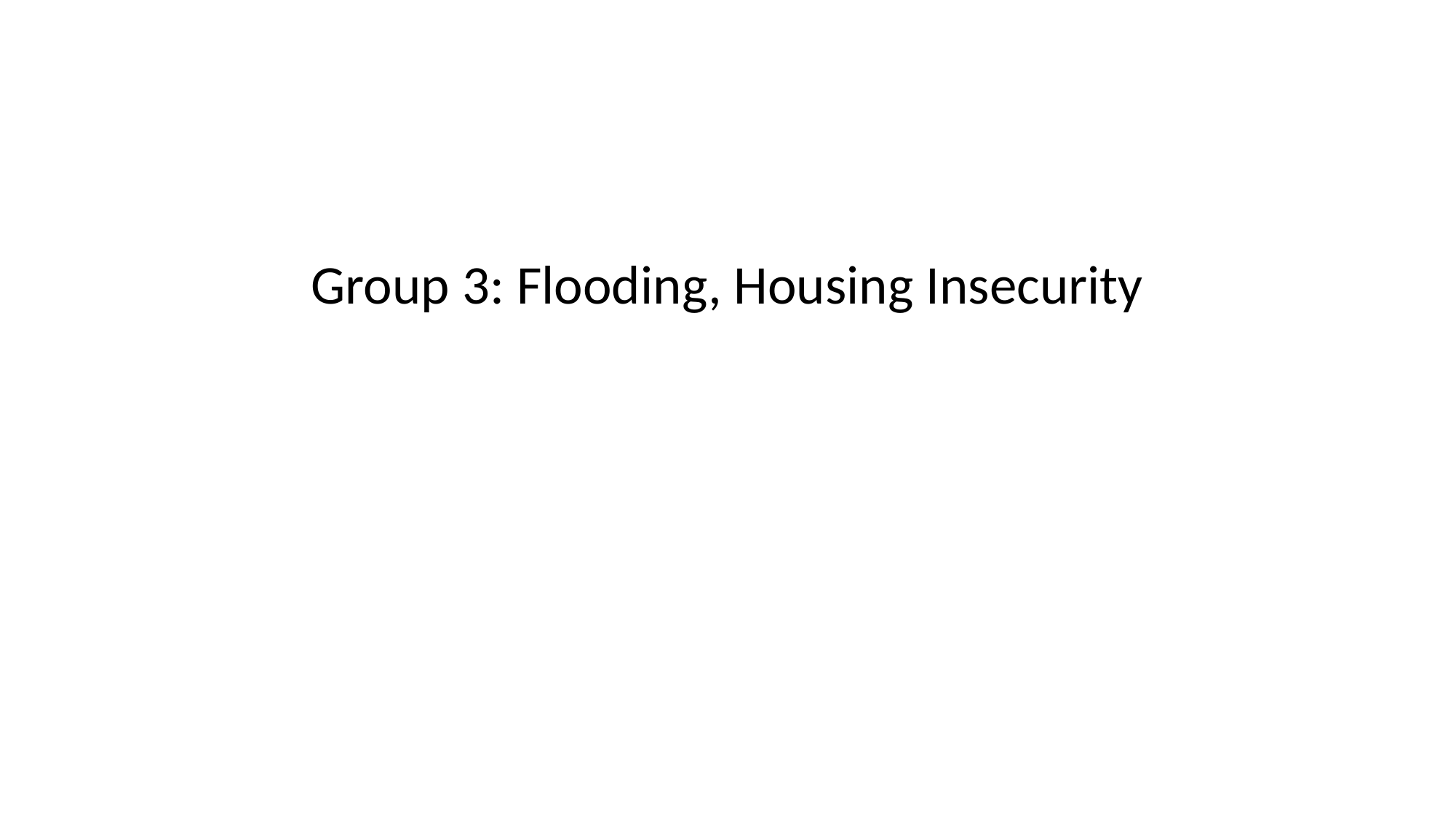

# Group 3: Flooding, Housing Insecurity

## Slide 17
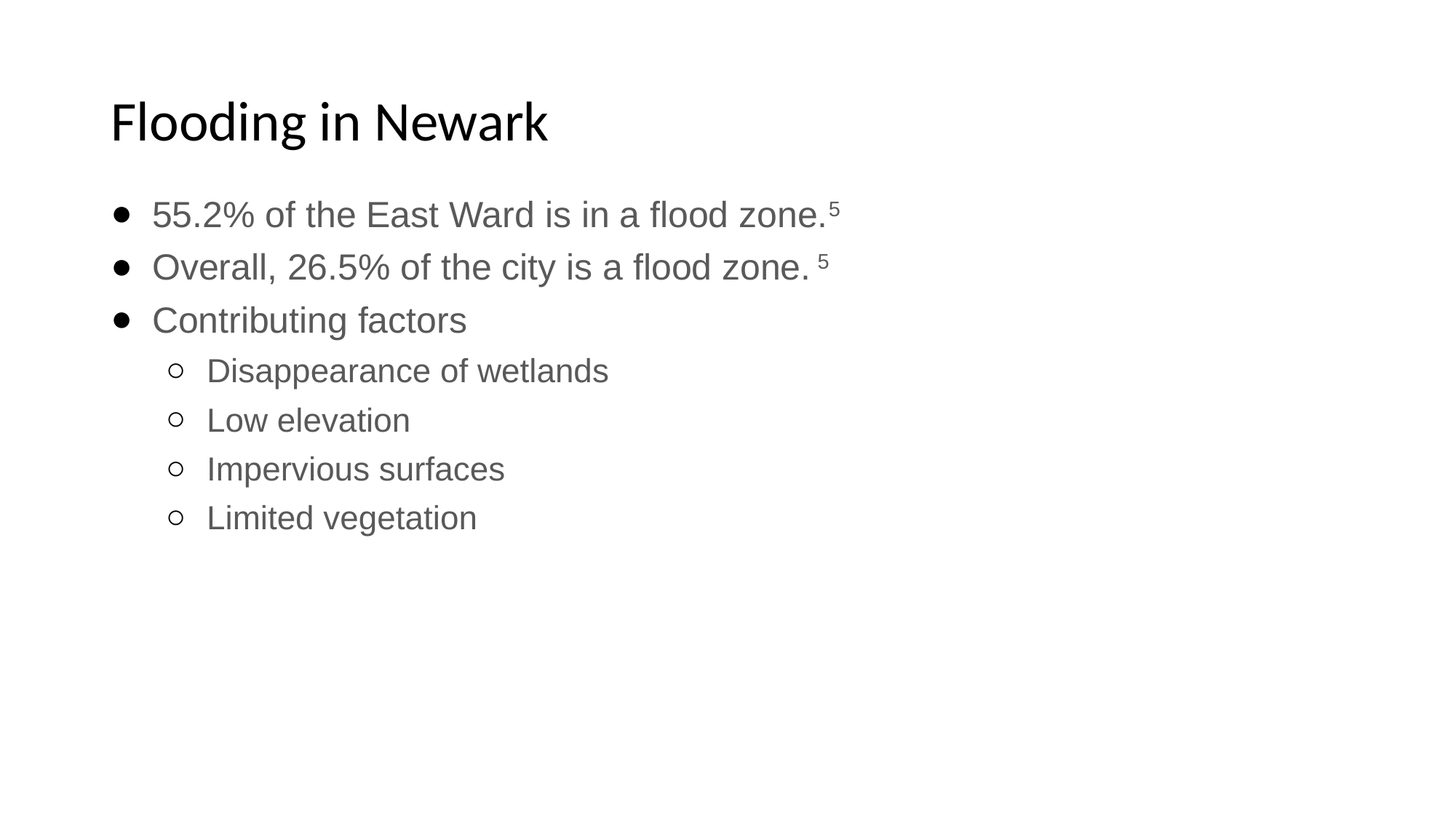

# Flooding in Newark
55.2% of the East Ward is in a flood zone.5
Overall, 26.5% of the city is a flood zone. 5
Contributing factors
Disappearance of wetlands
Low elevation
Impervious surfaces
Limited vegetation

## Slide 18
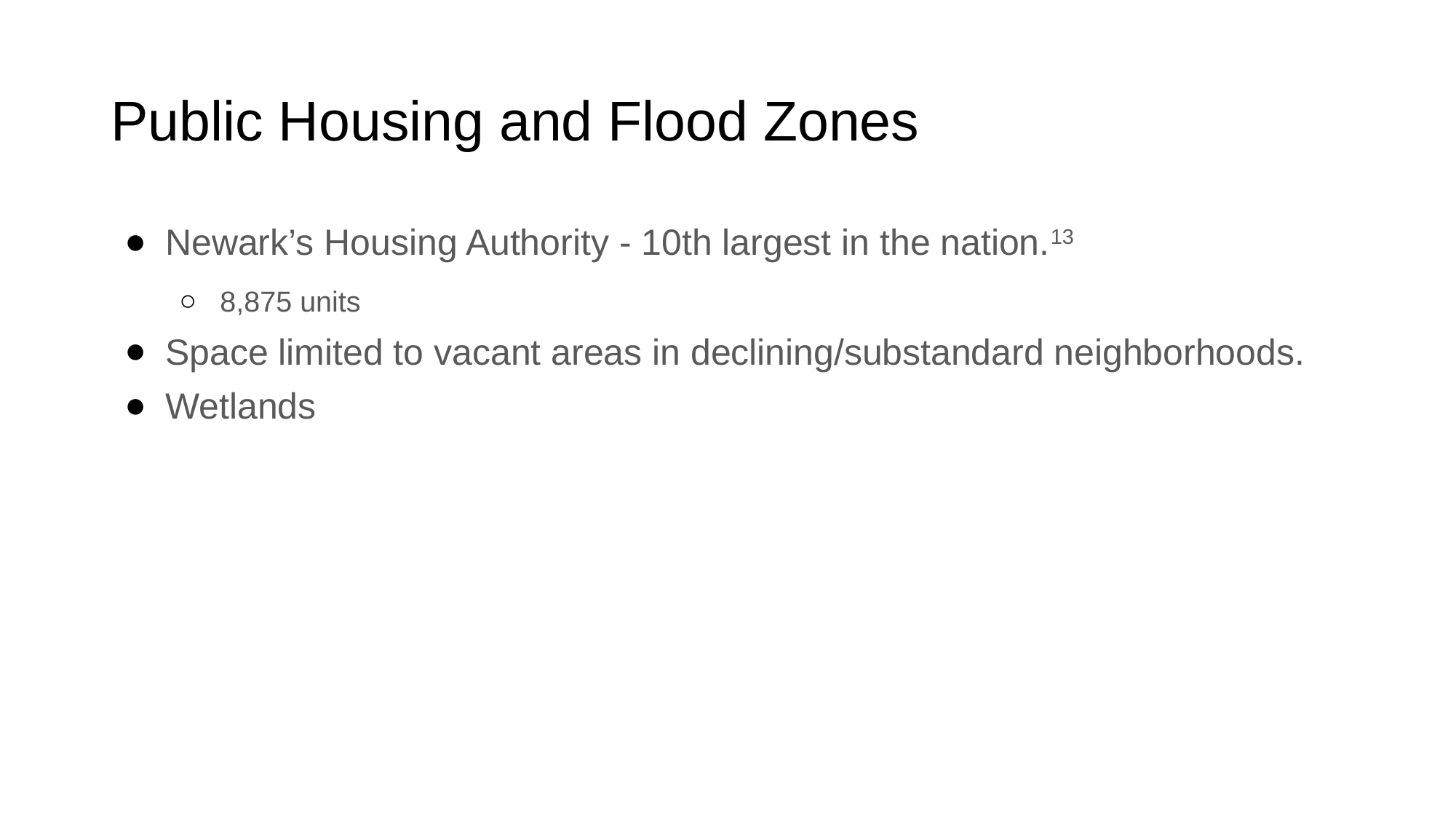

# Public Housing and Flood Zones
Newark’s Housing Authority - 10th largest in the nation.13
8,875 units
Space limited to vacant areas in declining/substandard neighborhoods.
Wetlands

## Slide 19
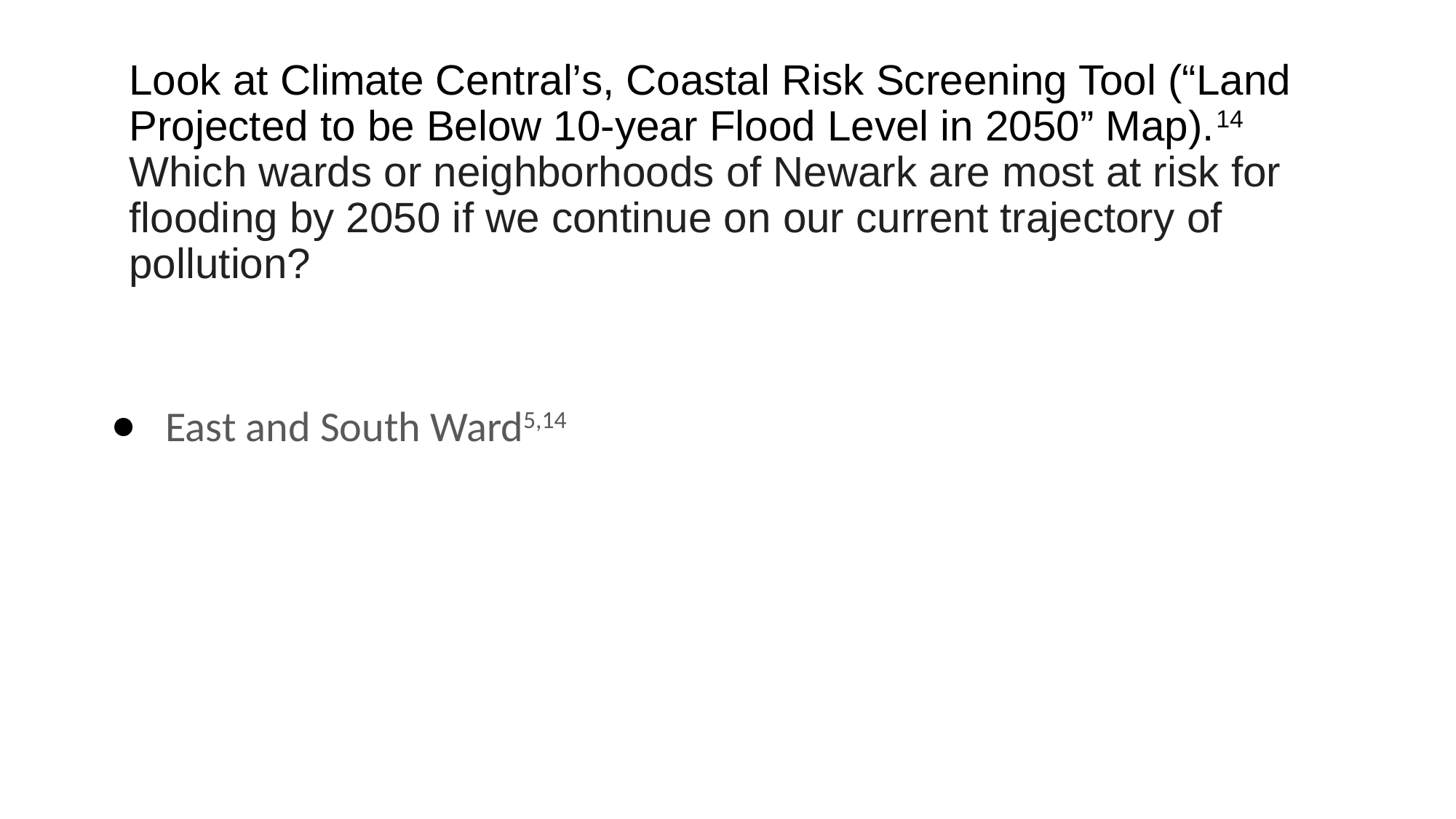

# Look at Climate Central’s, Coastal Risk Screening Tool (“Land Projected to be Below 10-year Flood Level in 2050” Map).14 Which wards or neighborhoods of Newark are most at risk for flooding by 2050 if we continue on our current trajectory of pollution?
East and South Ward5,14

## Slide 20
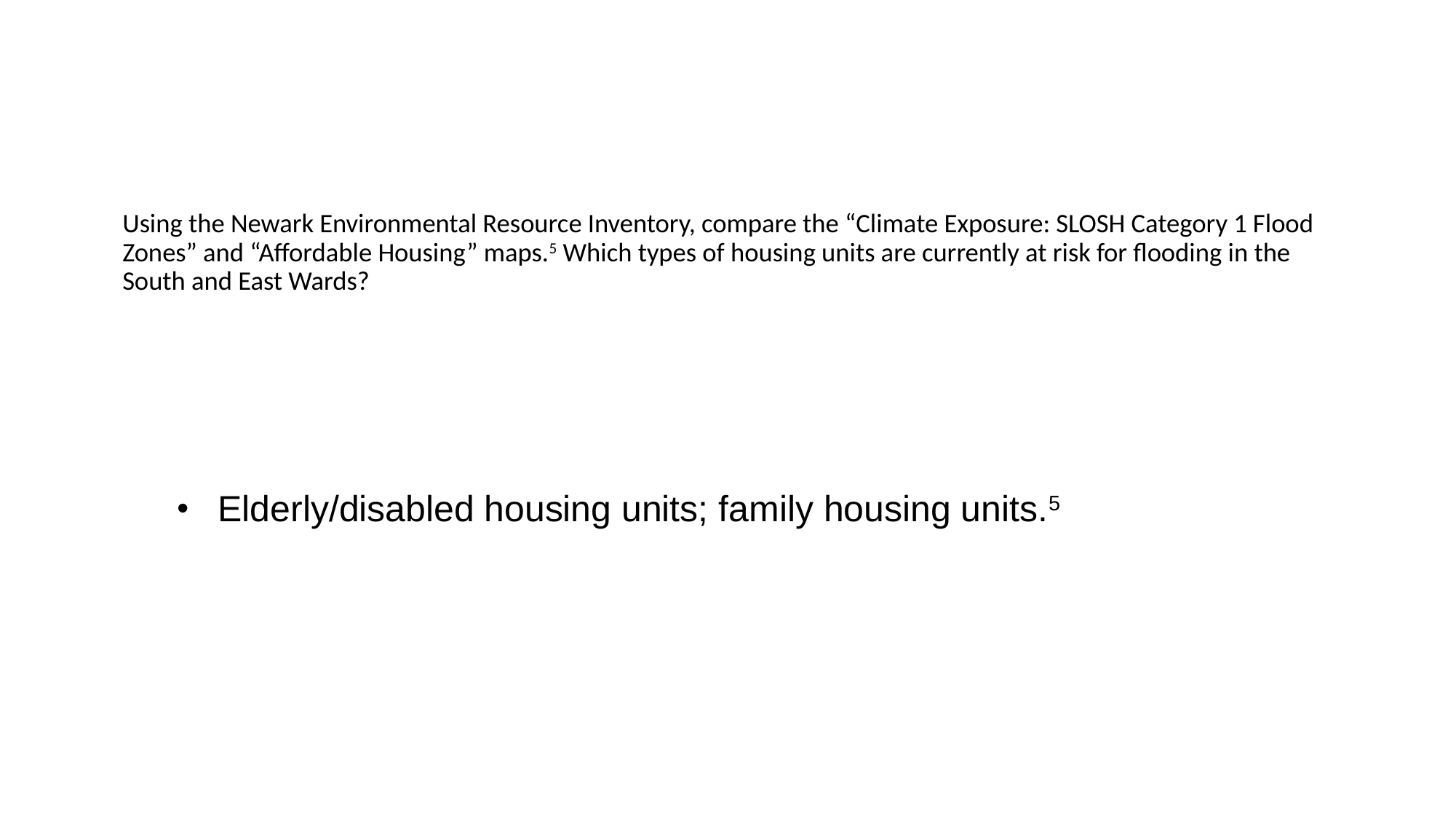

# Using the Newark Environmental Resource Inventory, compare the “Climate Exposure: SLOSH Category 1 Flood Zones” and “Affordable Housing” maps.5 Which types of housing units are currently at risk for flooding in the South and East Wards?
Elderly/disabled housing units; family housing units.5

## Slide 21
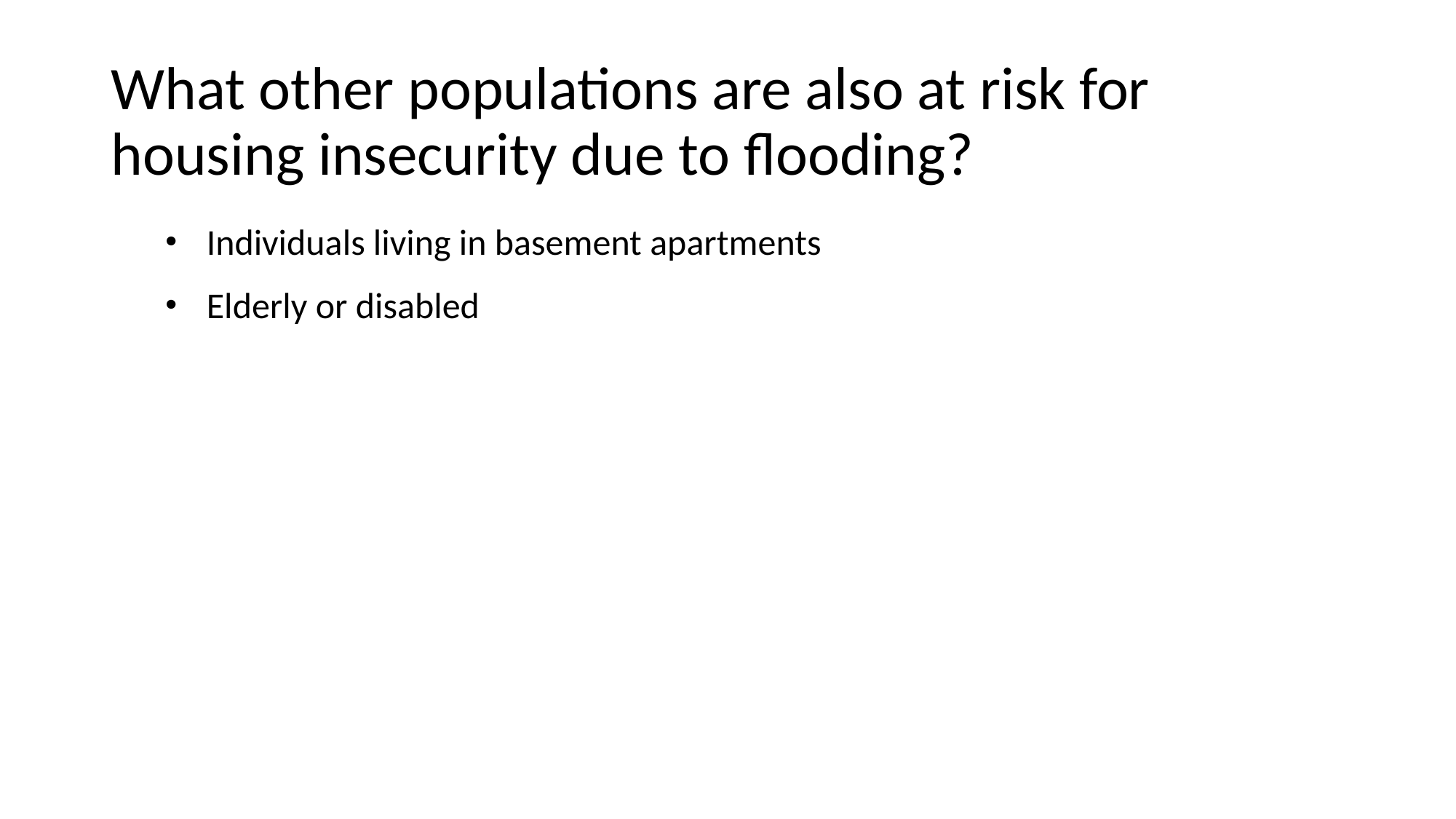

# What other populations are also at risk for housing insecurity due to flooding?
Individuals living in basement apartments
Elderly or disabled

## Slide 22
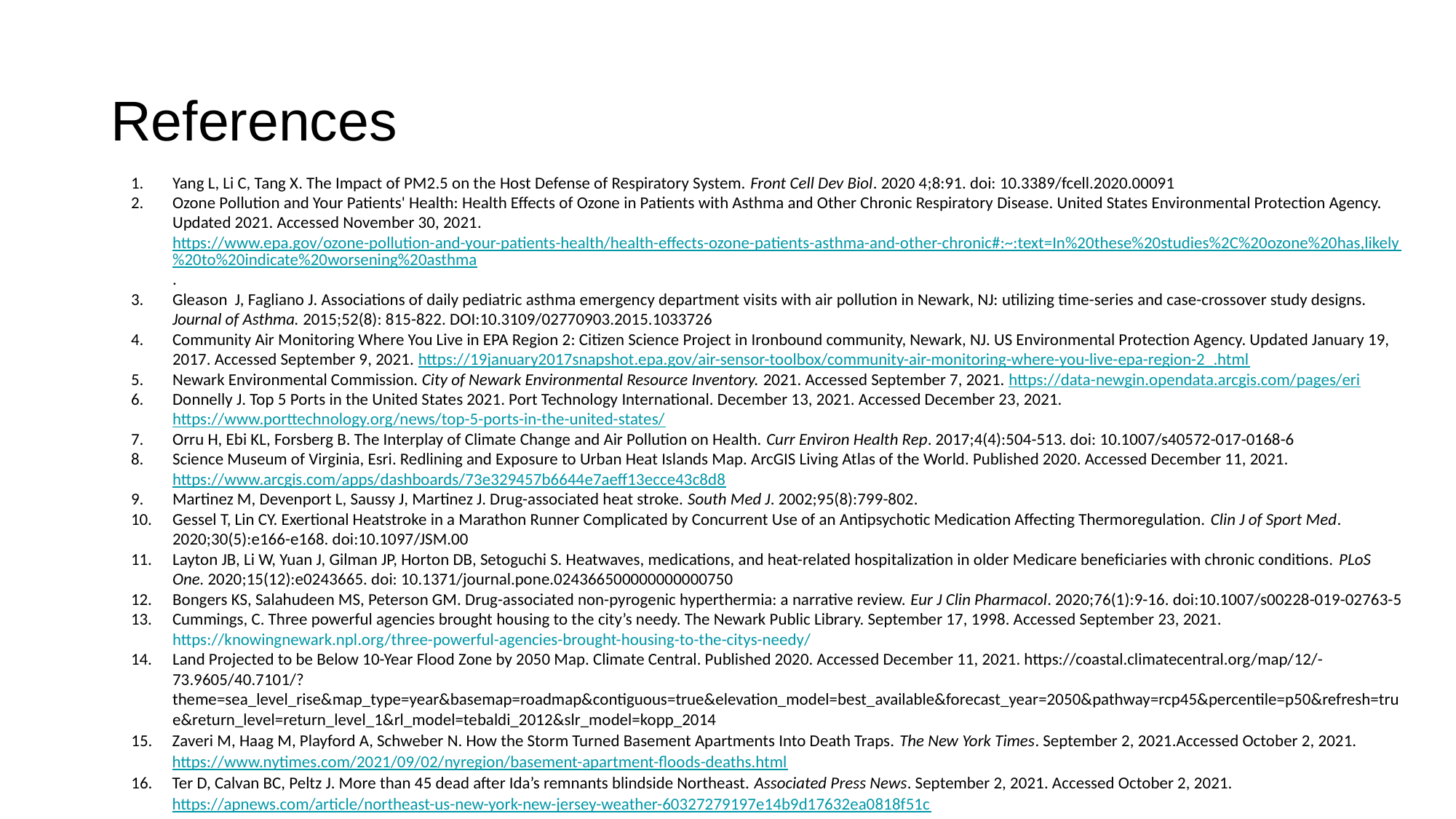

# References
Yang L, Li C, Tang X. The Impact of PM2.5 on the Host Defense of Respiratory System. Front Cell Dev Biol. 2020 4;8:91. doi: 10.3389/fcell.2020.00091
Ozone Pollution and Your Patients' Health: Health Effects of Ozone in Patients with Asthma and Other Chronic Respiratory Disease. United States Environmental Protection Agency. Updated 2021. Accessed November 30, 2021. https://www.epa.gov/ozone-pollution-and-your-patients-health/health-effects-ozone-patients-asthma-and-other-chronic#:~:text=In%20these%20studies%2C%20ozone%20has,likely%20to%20indicate%20worsening%20asthma.
Gleason J, Fagliano J. Associations of daily pediatric asthma emergency department visits with air pollution in Newark, NJ: utilizing time-series and case-crossover study designs. Journal of Asthma. 2015;52(8): 815-822. DOI:10.3109/02770903.2015.1033726
Community Air Monitoring Where You Live in EPA Region 2: Citizen Science Project in Ironbound community, Newark, NJ. US Environmental Protection Agency. Updated January 19, 2017. Accessed September 9, 2021. https://19january2017snapshot.epa.gov/air-sensor-toolbox/community-air-monitoring-where-you-live-epa-region-2_.html
Newark Environmental Commission. City of Newark Environmental Resource Inventory. 2021. Accessed September 7, 2021. https://data-newgin.opendata.arcgis.com/pages/eri
Donnelly J. Top 5 Ports in the United States 2021. Port Technology International. December 13, 2021. Accessed December 23, 2021. https://www.porttechnology.org/news/top-5-ports-in-the-united-states/
Orru H, Ebi KL, Forsberg B. The Interplay of Climate Change and Air Pollution on Health. Curr Environ Health Rep. 2017;4(4):504-513. doi: 10.1007/s40572-017-0168-6
Science Museum of Virginia, Esri. Redlining and Exposure to Urban Heat Islands Map. ArcGIS Living Atlas of the World. Published 2020. Accessed December 11, 2021. https://www.arcgis.com/apps/dashboards/73e329457b6644e7aeff13ecce43c8d8
Martinez M, Devenport L, Saussy J, Martinez J. Drug-associated heat stroke. South Med J. 2002;95(8):799-802.
Gessel T, Lin CY. Exertional Heatstroke in a Marathon Runner Complicated by Concurrent Use of an Antipsychotic Medication Affecting Thermoregulation. Clin J of Sport Med. 2020;30(5):e166-e168. doi:10.1097/JSM.00
Layton JB, Li W, Yuan J, Gilman JP, Horton DB, Setoguchi S. Heatwaves, medications, and heat-related hospitalization in older Medicare beneficiaries with chronic conditions. PLoS One. 2020;15(12):e0243665. doi: 10.1371/journal.pone.024366500000000000750
Bongers KS, Salahudeen MS, Peterson GM. Drug-associated non-pyrogenic hyperthermia: a narrative review. Eur J Clin Pharmacol. 2020;76(1):9-16. doi:10.1007/s00228-019-02763-5
Cummings, C. Three powerful agencies brought housing to the city’s needy. The Newark Public Library. September 17, 1998. Accessed September 23, 2021. https://knowingnewark.npl.org/three-powerful-agencies-brought-housing-to-the-citys-needy/
Land Projected to be Below 10-Year Flood Zone by 2050 Map. Climate Central. Published 2020. Accessed December 11, 2021. https://coastal.climatecentral.org/map/12/-73.9605/40.7101/?theme=sea_level_rise&map_type=year&basemap=roadmap&contiguous=true&elevation_model=best_available&forecast_year=2050&pathway=rcp45&percentile=p50&refresh=true&return_level=return_level_1&rl_model=tebaldi_2012&slr_model=kopp_2014
Zaveri M, Haag M, Playford A, Schweber N. How the Storm Turned Basement Apartments Into Death Traps. The New York Times. September 2, 2021.Accessed October 2, 2021. https://www.nytimes.com/2021/09/02/nyregion/basement-apartment-floods-deaths.html
Ter D, Calvan BC, Peltz J. More than 45 dead after Ida’s remnants blindside Northeast. Associated Press News. September 2, 2021. Accessed October 2, 2021. https://apnews.com/article/northeast-us-new-york-new-jersey-weather-60327279197e14b9d17632ea0818f51c

## Slide 23
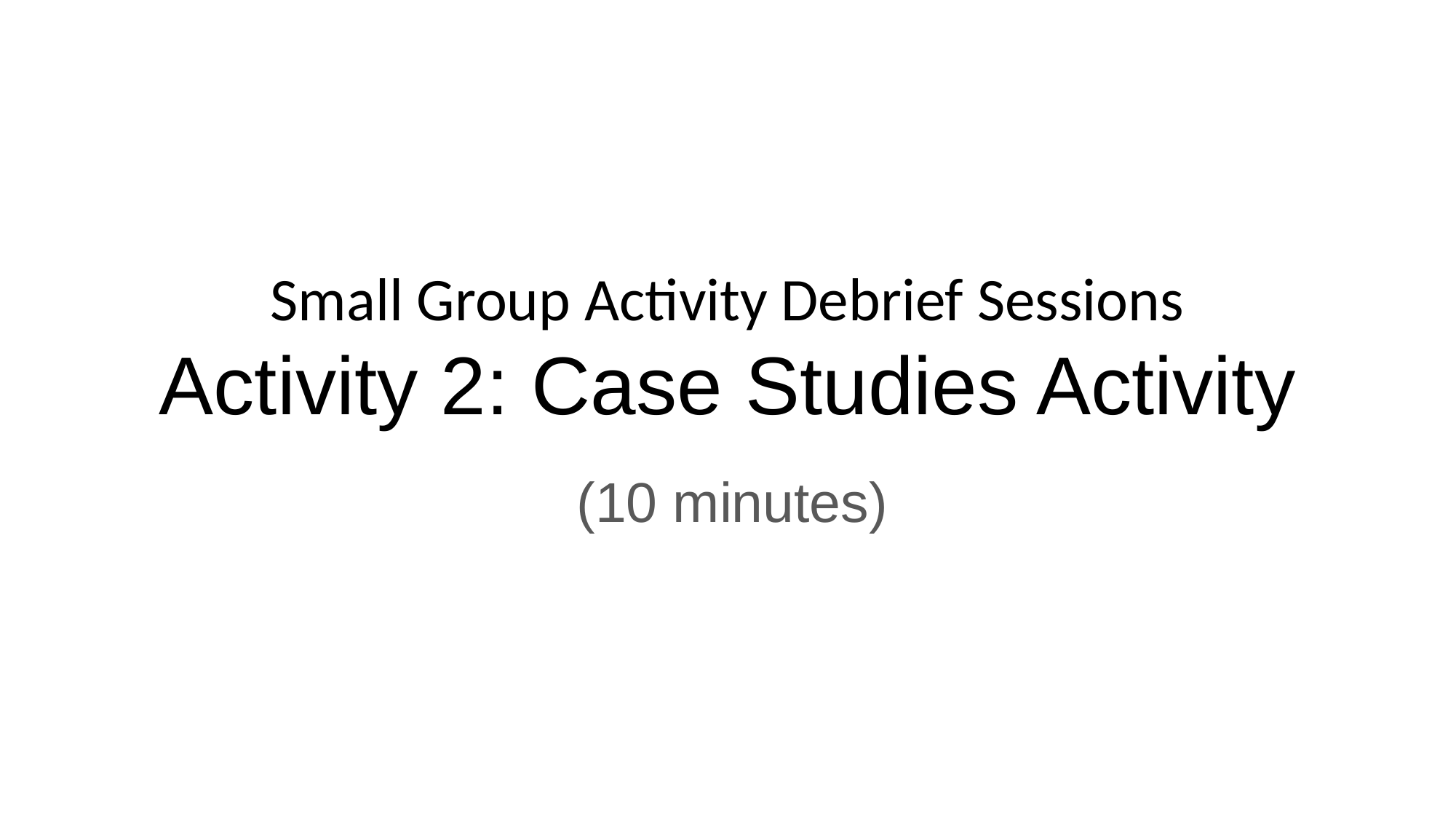

# Small Group Activity Debrief Sessions Activity 2: Case Studies Activity
(10 minutes)

## Slide 24
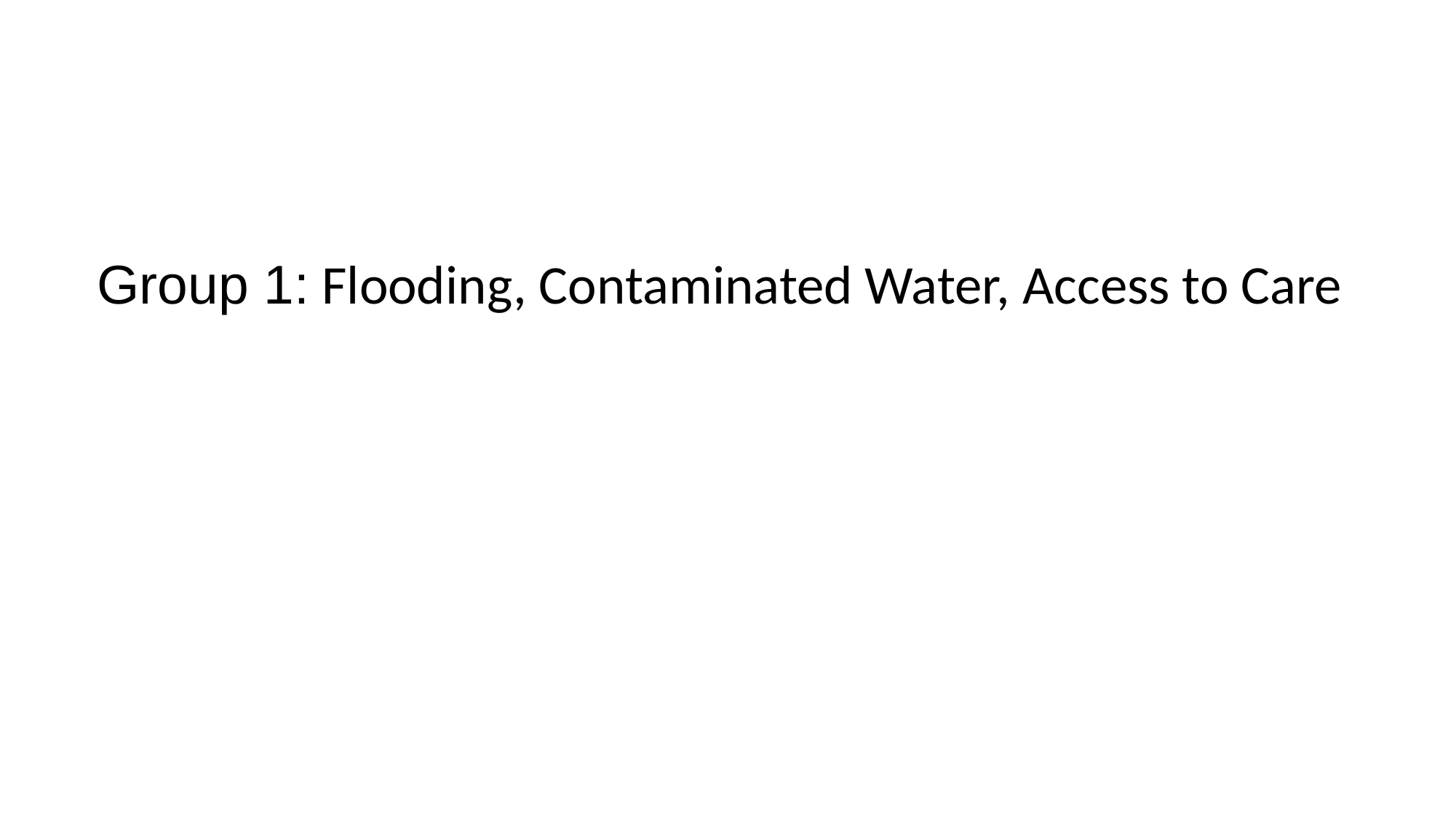

# Group 1: Flooding, Contaminated Water, Access to Care

## Slide 25
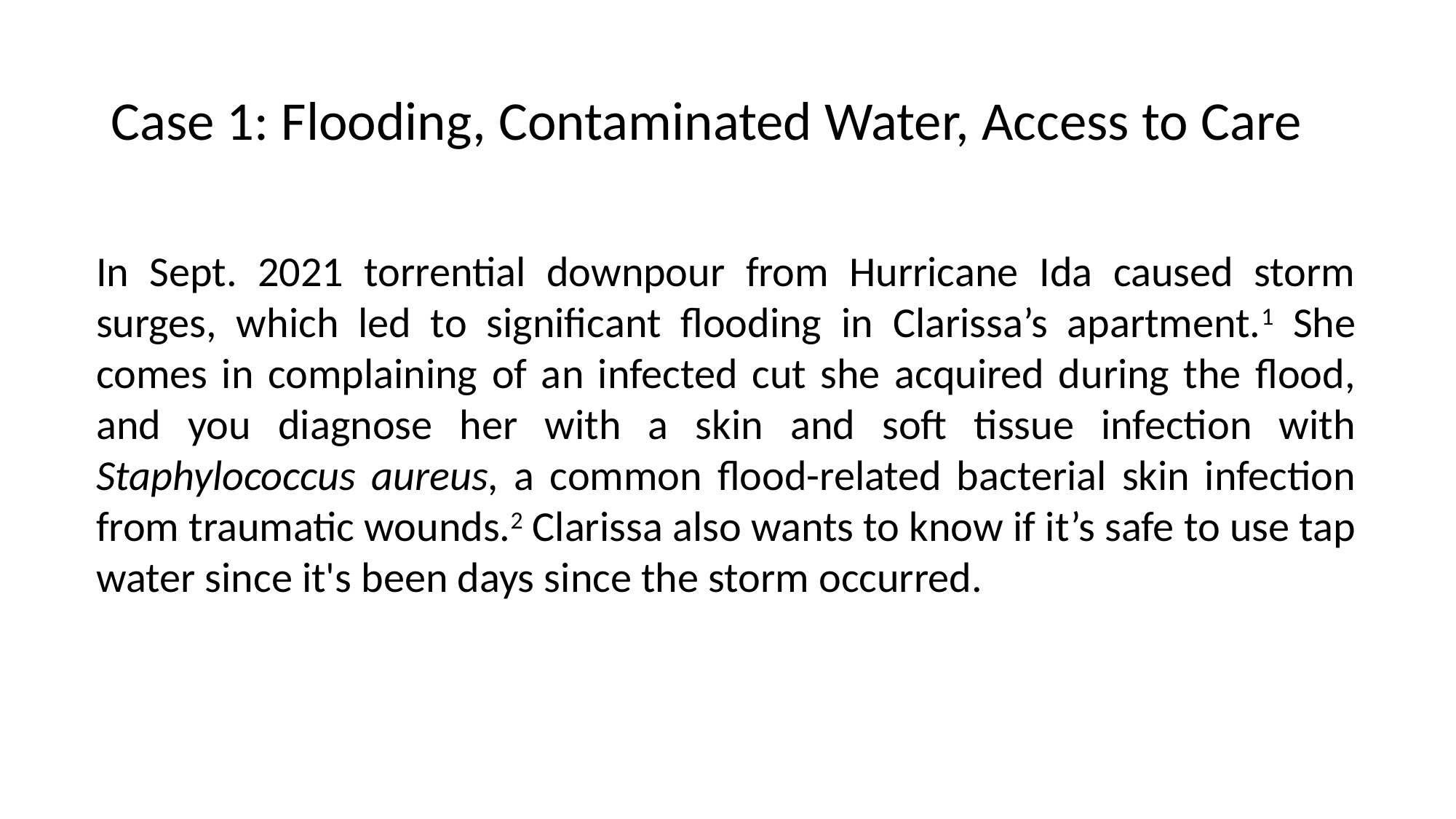

# Case 1: Flooding, Contaminated Water, Access to Care
In Sept. 2021 torrential downpour from Hurricane Ida caused storm surges, which led to significant flooding in Clarissa’s apartment.1 She comes in complaining of an infected cut she acquired during the flood, and you diagnose her with a skin and soft tissue infection with Staphylococcus aureus, a common flood-related bacterial skin infection from traumatic wounds.2 Clarissa also wants to know if it’s safe to use tap water since it's been days since the storm occurred.

## Slide 26
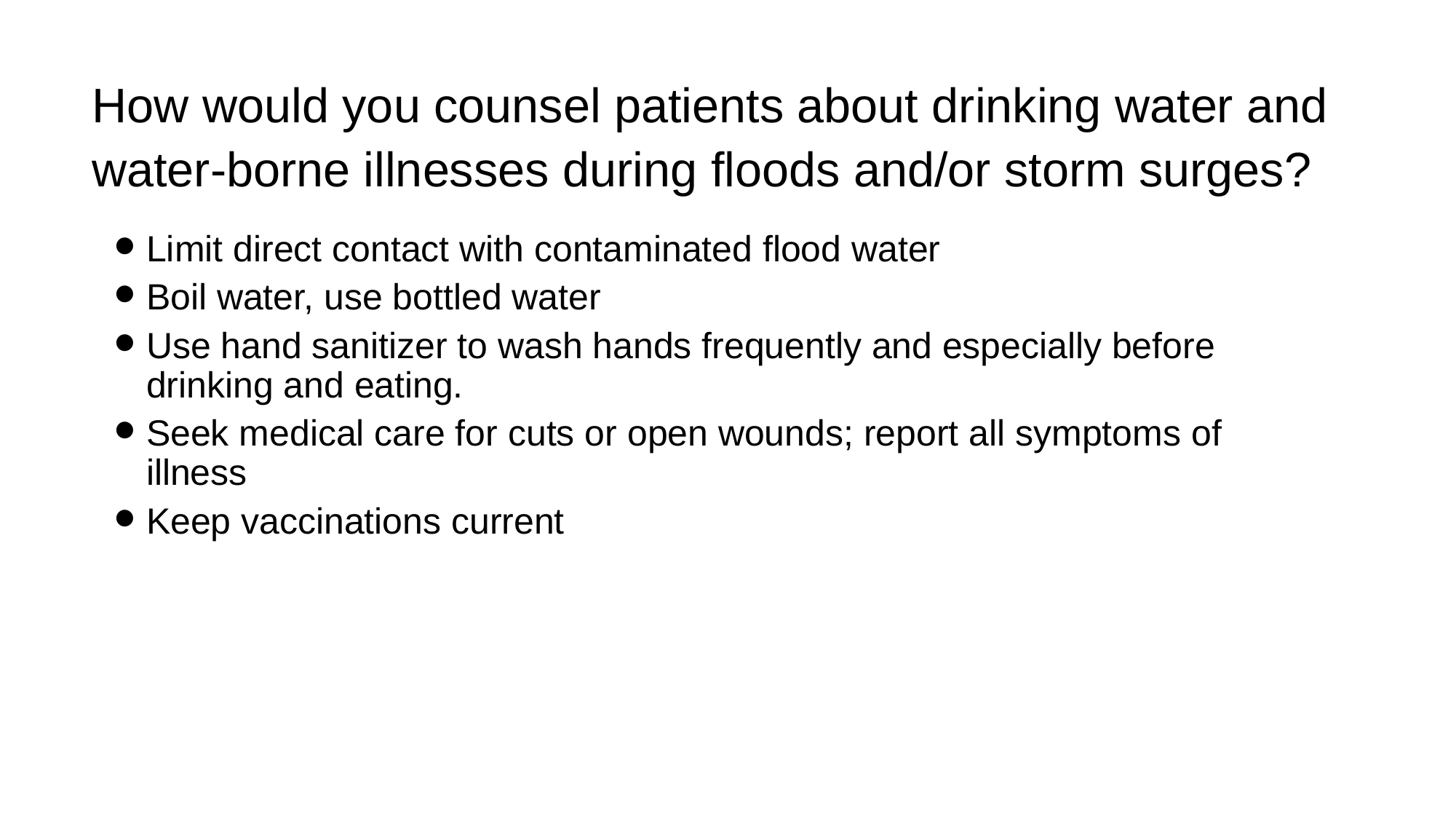

How would you counsel patients about drinking water and water-borne illnesses during floods and/or storm surges?
Limit direct contact with contaminated flood water
Boil water, use bottled water
Use hand sanitizer to wash hands frequently and especially before drinking and eating.
Seek medical care for cuts or open wounds; report all symptoms of illness
Keep vaccinations current

## Slide 27
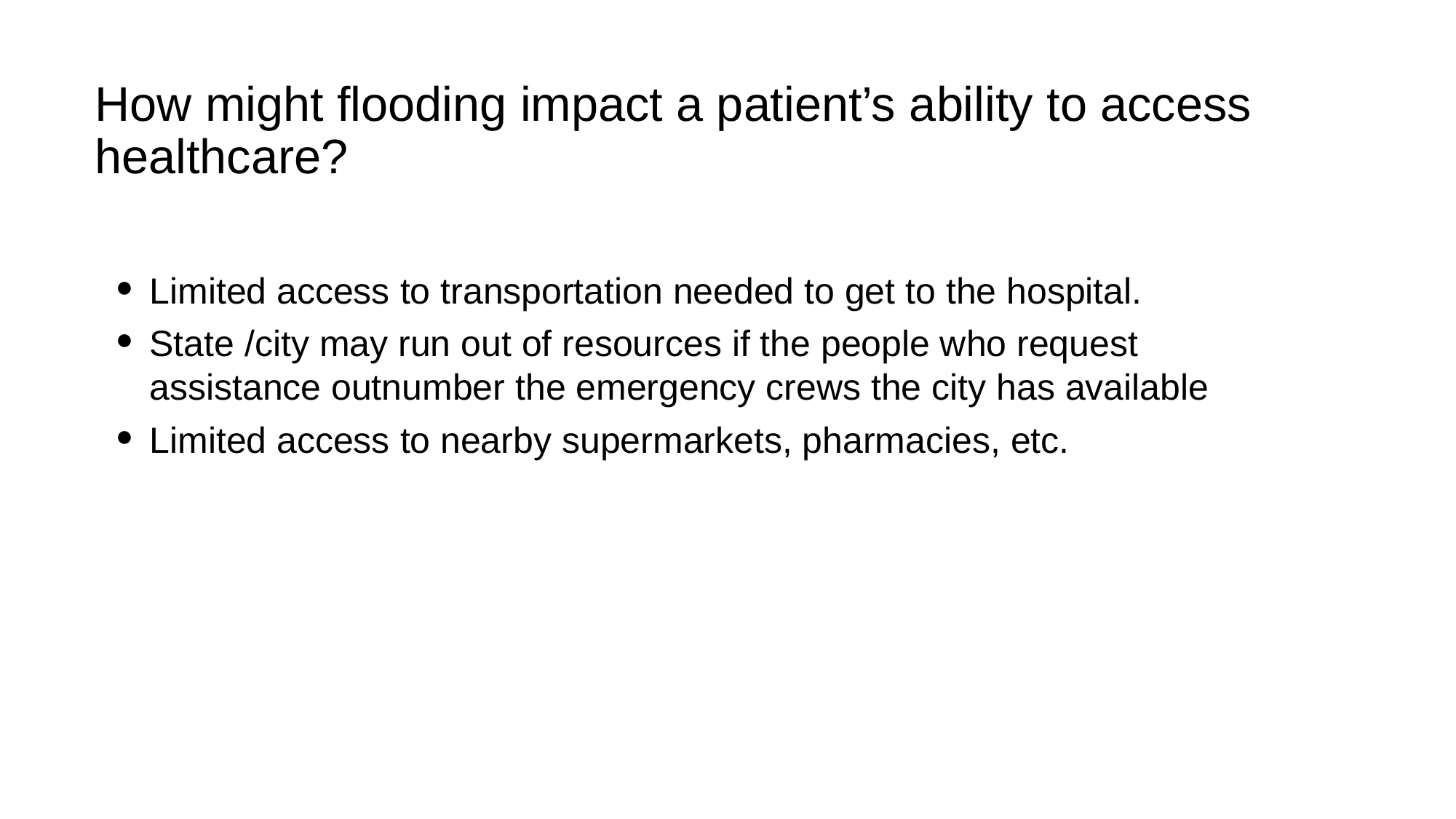

How might flooding impact a patient’s ability to access healthcare?
Limited access to transportation needed to get to the hospital.
State /city may run out of resources if the people who request assistance outnumber the emergency crews the city has available
Limited access to nearby supermarkets, pharmacies, etc.

## Slide 28
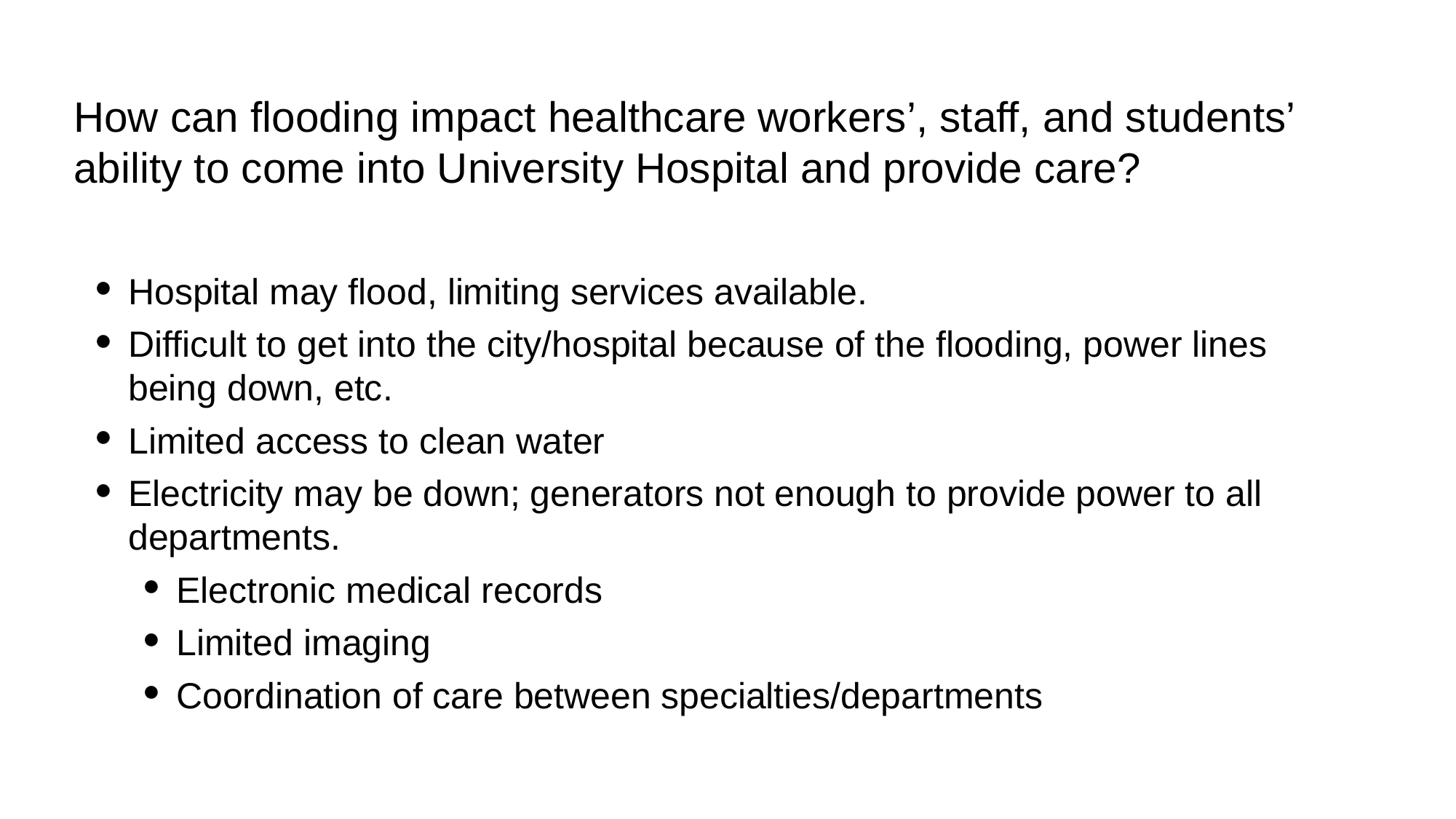

How can flooding impact healthcare workers’, staff, and students’ ability to come into University Hospital and provide care?
Hospital may flood, limiting services available.
Difficult to get into the city/hospital because of the flooding, power lines being down, etc.
Limited access to clean water
Electricity may be down; generators not enough to provide power to all departments.
Electronic medical records
Limited imaging
Coordination of care between specialties/departments

## Slide 29
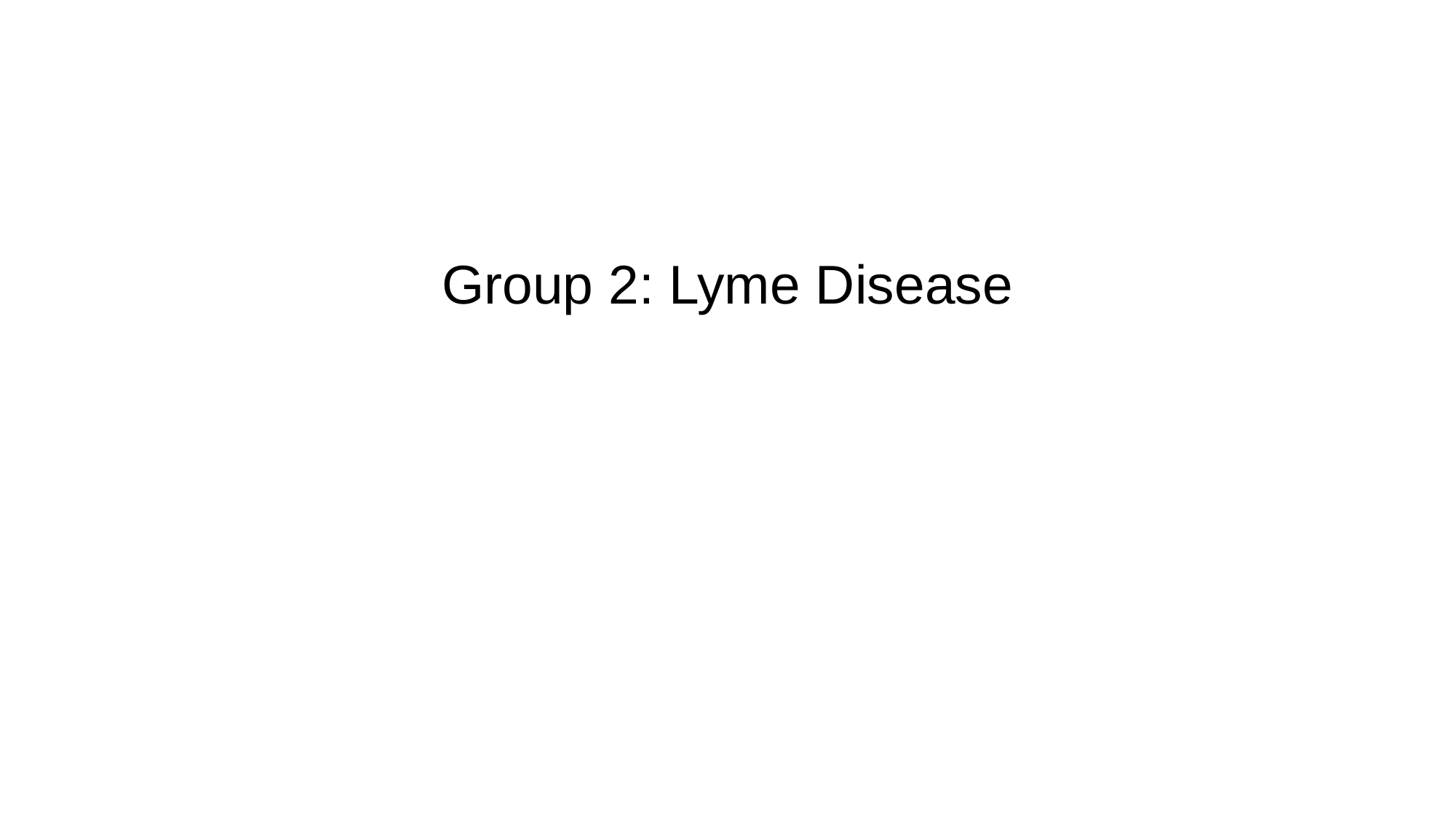

# Group 2: Lyme Disease

## Slide 30
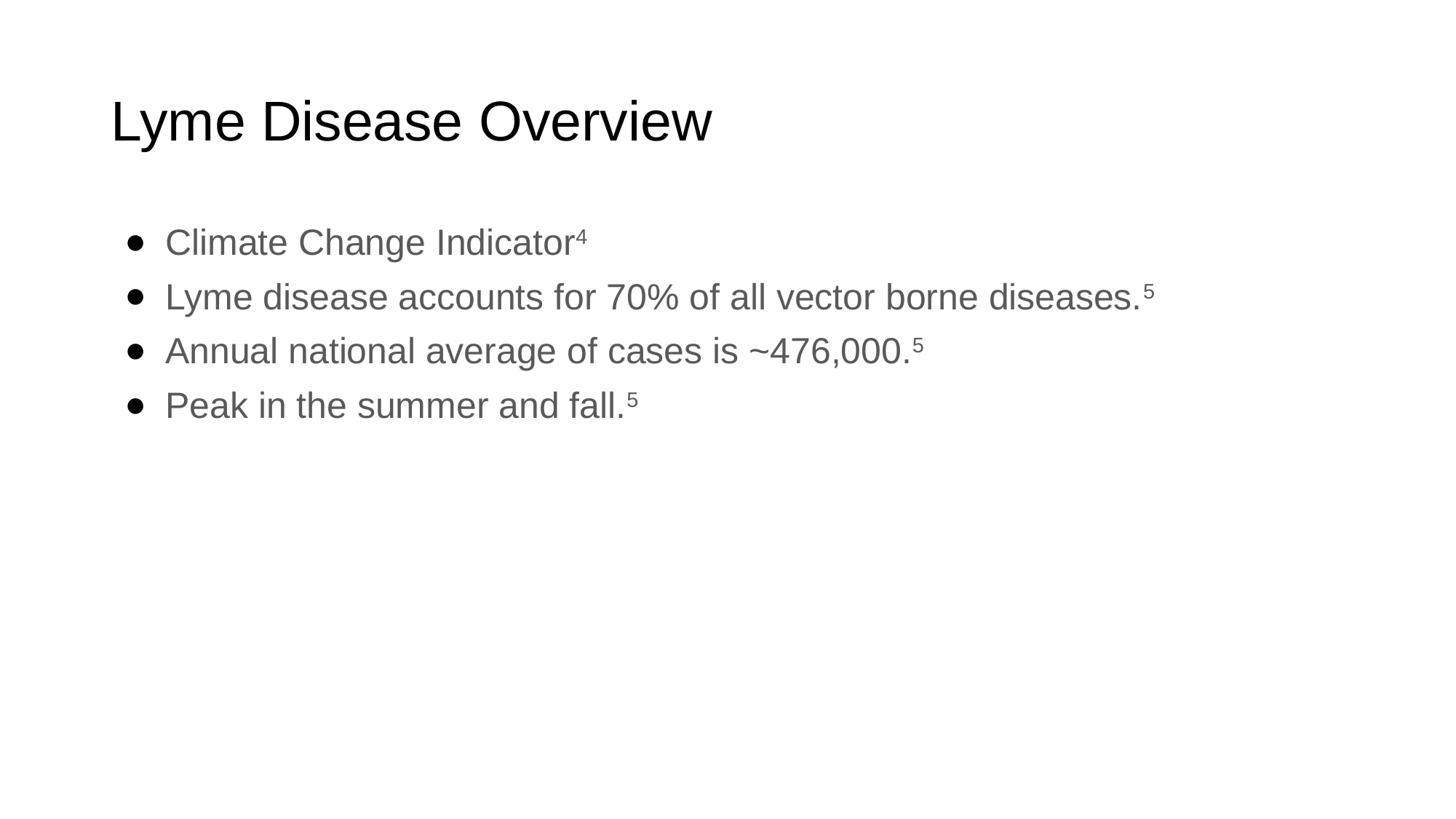

# Lyme Disease Overview
Climate Change Indicator4
Lyme disease accounts for 70% of all vector borne diseases.5
Annual national average of cases is ~476,000.5
Peak in the summer and fall.5

## Slide 31
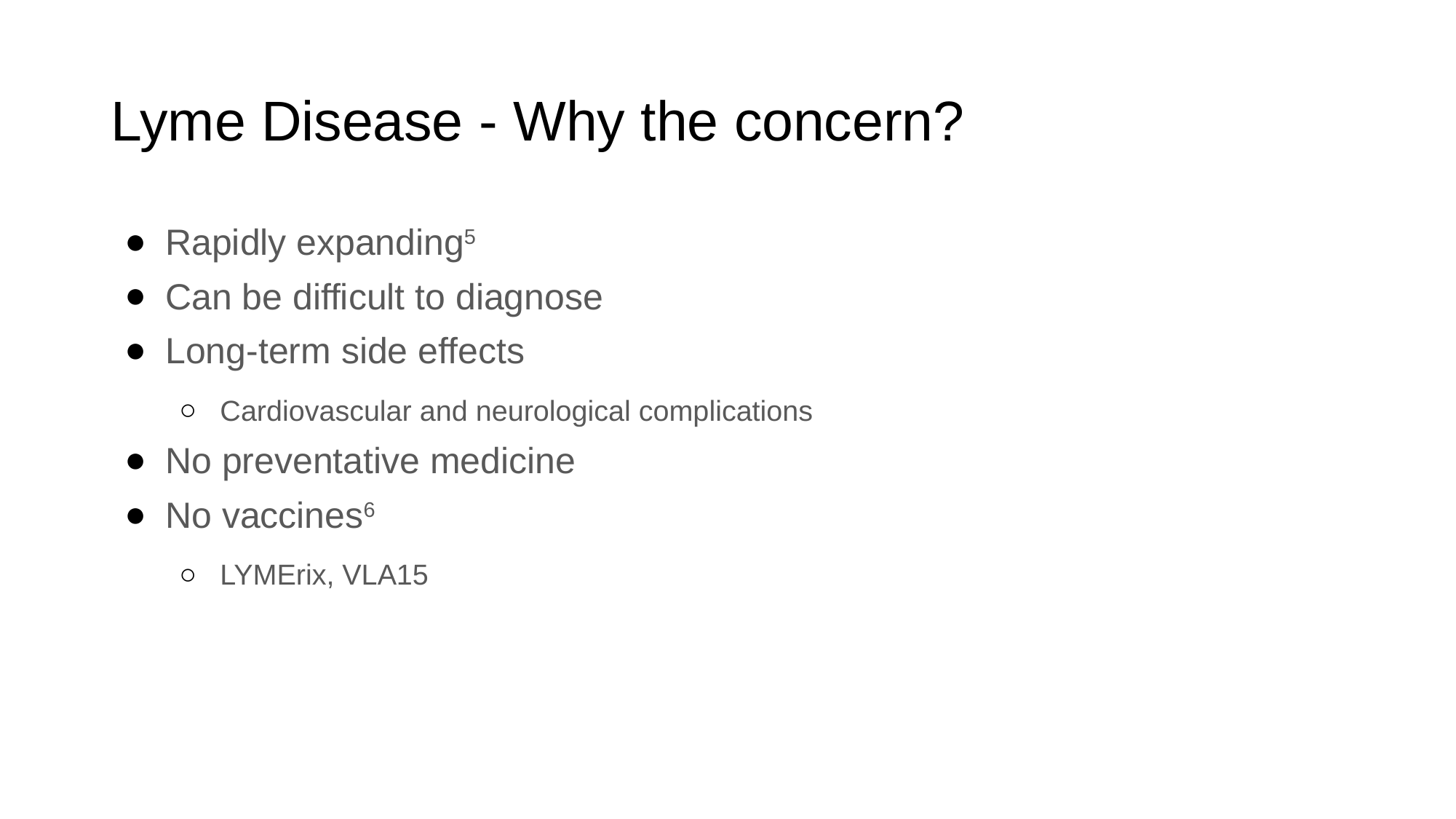

# Lyme Disease - Why the concern?
Rapidly expanding5
Can be difficult to diagnose
Long-term side effects
Cardiovascular and neurological complications
No preventative medicine
No vaccines6
LYMErix, VLA15

## Slide 32
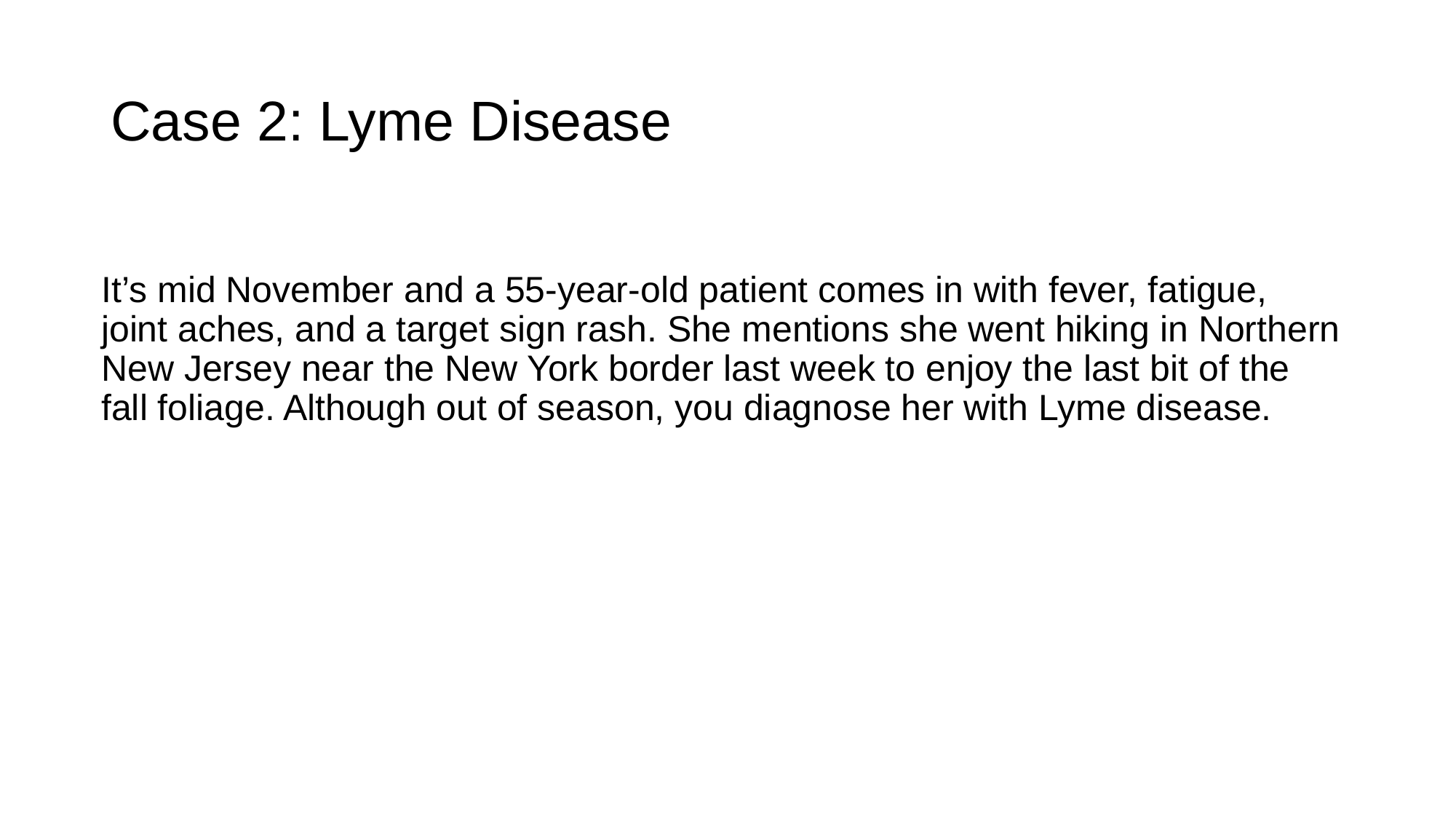

# Case 2: Lyme Disease
It’s mid November and a 55-year-old patient comes in with fever, fatigue, joint aches, and a target sign rash. She mentions she went hiking in Northern New Jersey near the New York border last week to enjoy the last bit of the fall foliage. Although out of season, you diagnose her with Lyme disease.

## Slide 33
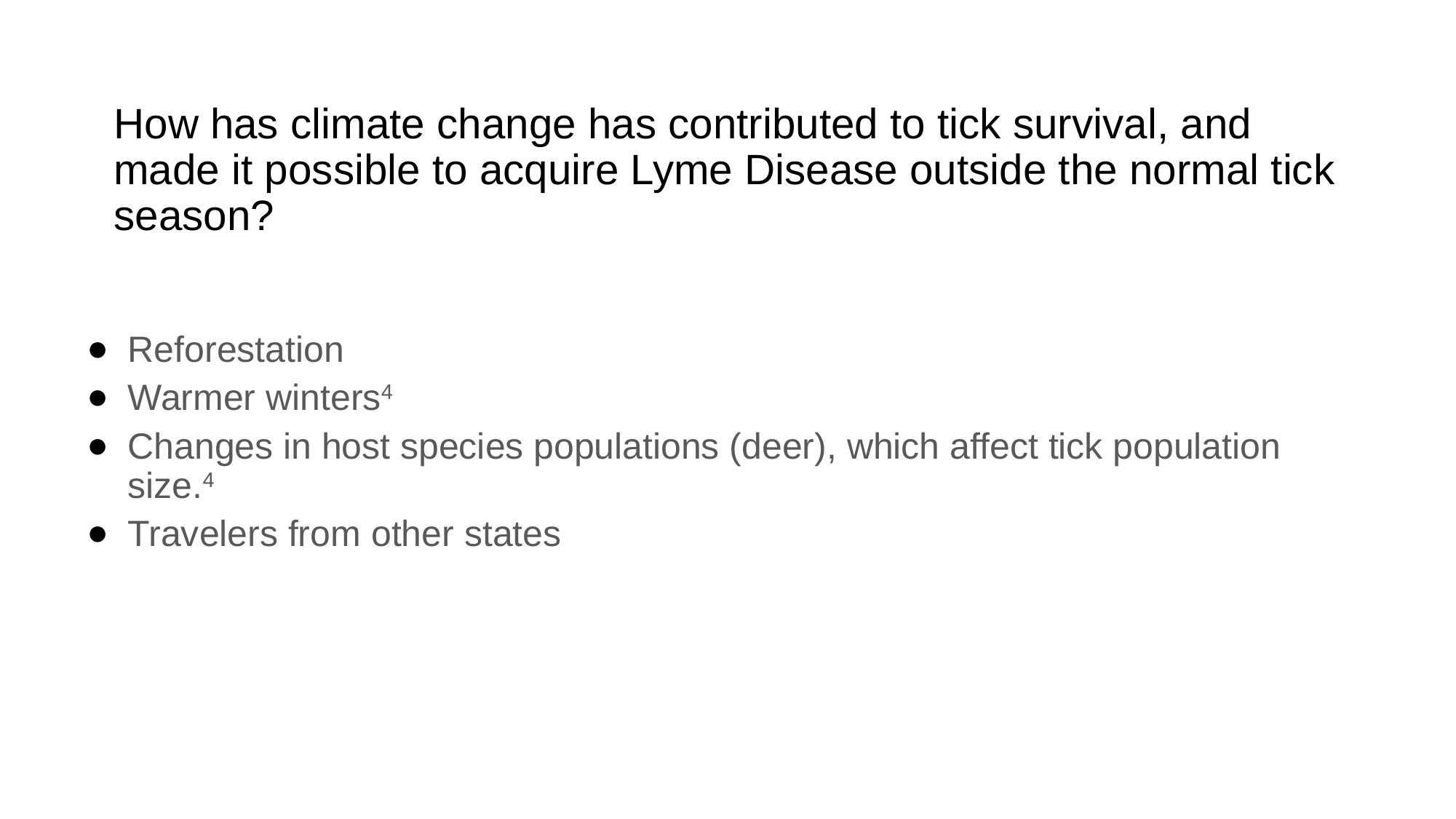

How has climate change has contributed to tick survival, and made it possible to acquire Lyme Disease outside the normal tick season?
Reforestation
Warmer winters4
Changes in host species populations (deer), which affect tick population size.4
Travelers from other states

## Slide 34
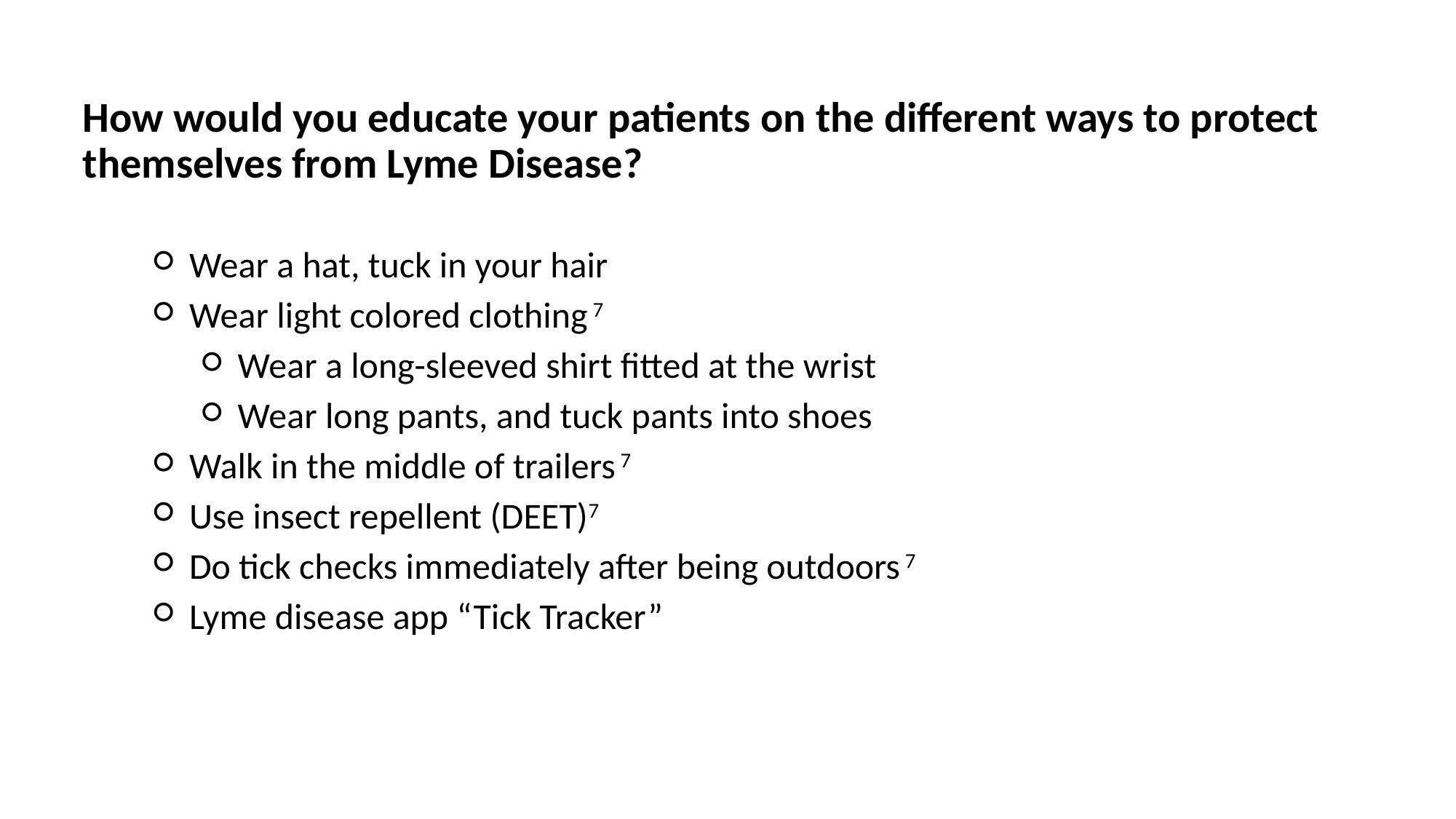

How would you educate your patients on the different ways to protect themselves from Lyme Disease?
Wear a hat, tuck in your hair
Wear light colored clothing 7
Wear a long-sleeved shirt fitted at the wrist
Wear long pants, and tuck pants into shoes
Walk in the middle of trailers 7
Use insect repellent (DEET)7
Do tick checks immediately after being outdoors 7
Lyme disease app “Tick Tracker”

## Slide 35
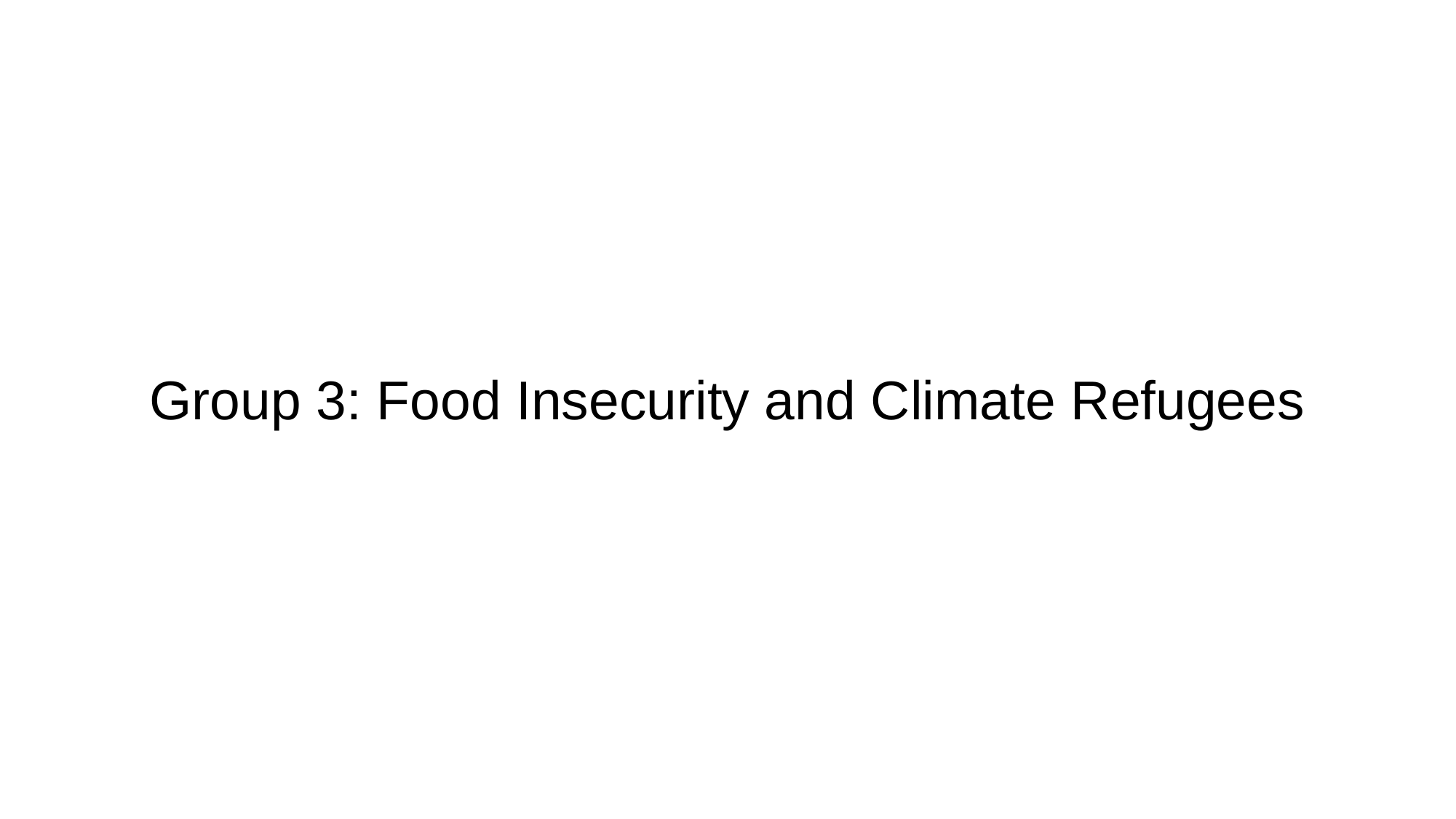

# Group 3: Food Insecurity and Climate Refugees

## Slide 36
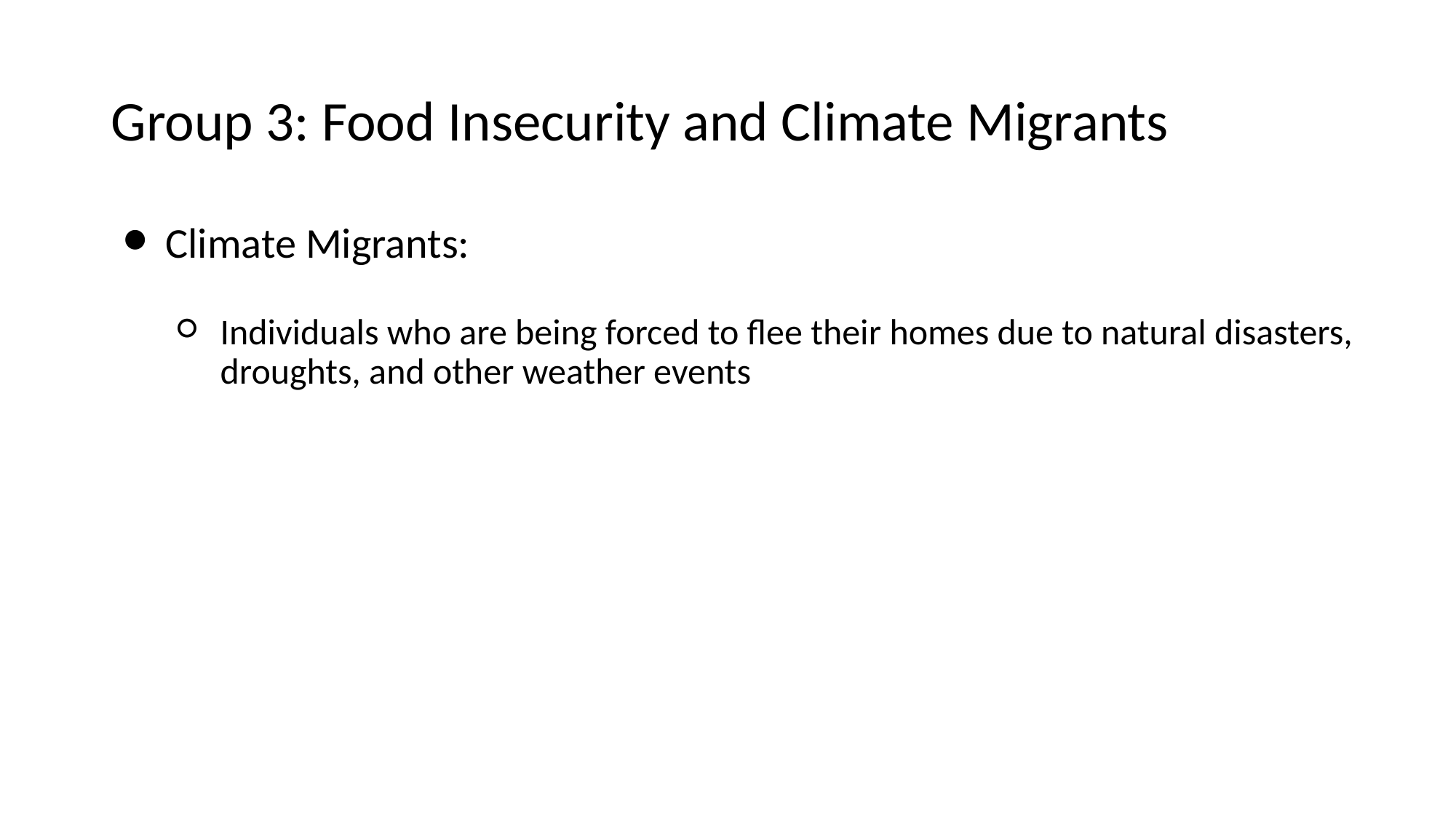

# Group 3: Food Insecurity and Climate Migrants
Climate Migrants:
Individuals who are being forced to flee their homes due to natural disasters, droughts, and other weather events

## Slide 37
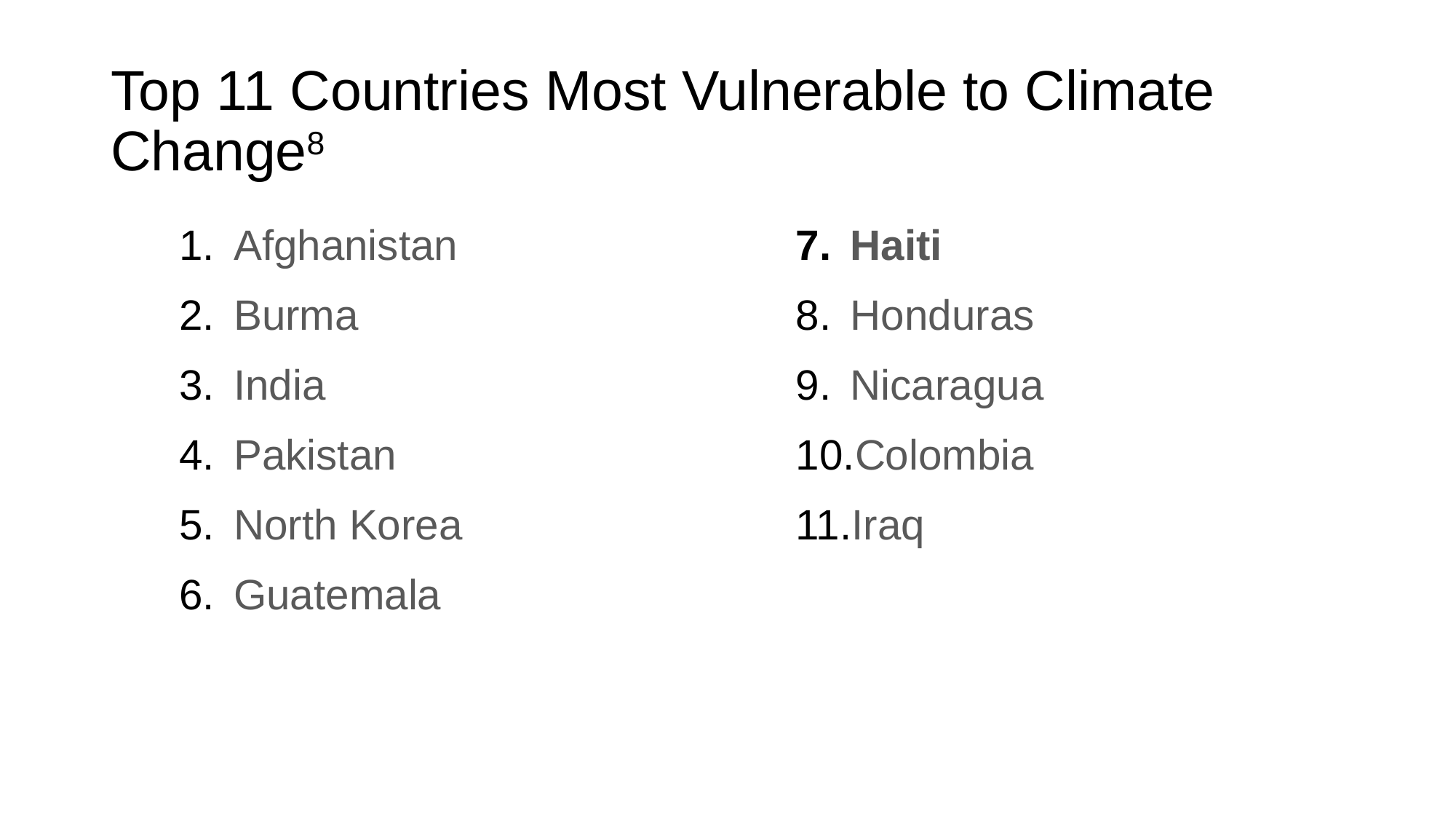

# Top 11 Countries Most Vulnerable to Climate Change8
Afghanistan
Burma
India
Pakistan
North Korea
Guatemala
Haiti
Honduras
Nicaragua
Colombia
Iraq

## Slide 38
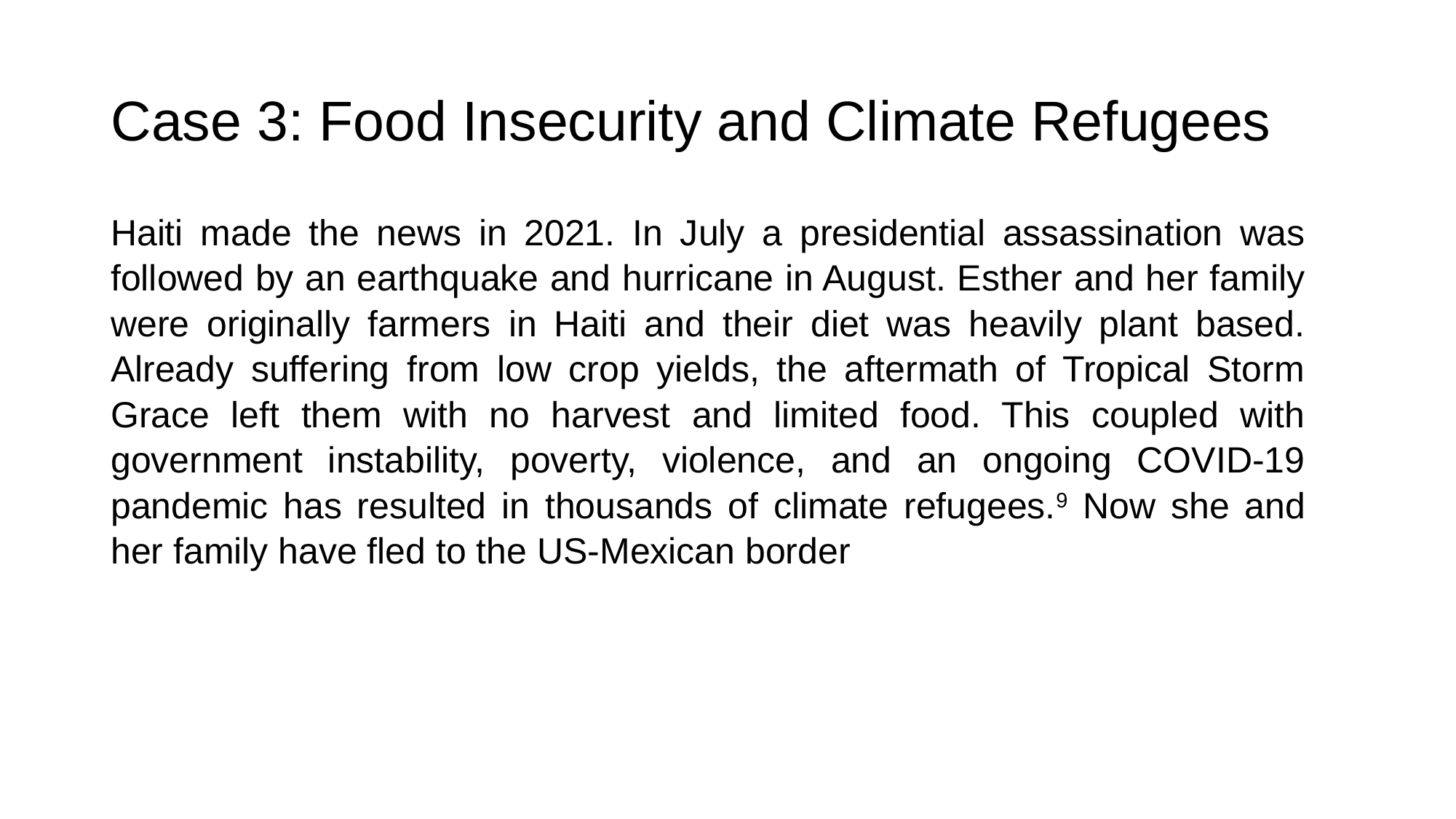

# Case 3: Food Insecurity and Climate Refugees
Haiti made the news in 2021. In July a presidential assassination was followed by an earthquake and hurricane in August. Esther and her family were originally farmers in Haiti and their diet was heavily plant based. Already suffering from low crop yields, the aftermath of Tropical Storm Grace left them with no harvest and limited food. This coupled with government instability, poverty, violence, and an ongoing COVID-19 pandemic has resulted in thousands of climate refugees.9 Now she and her family have fled to the US-Mexican border

## Slide 39
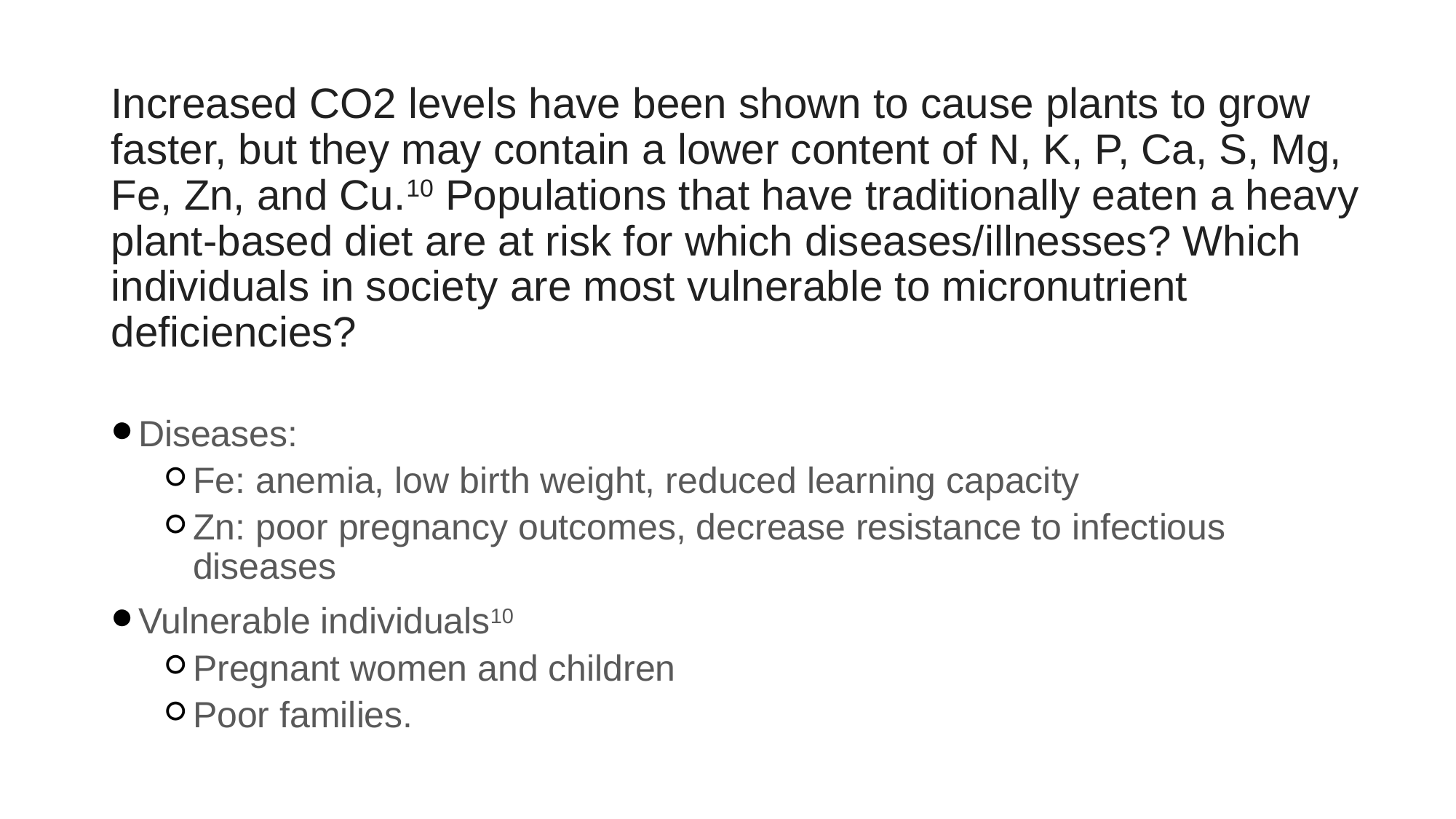

Increased CO2 levels have been shown to cause plants to grow faster, but they may contain a lower content of N, K, P, Ca, S, Mg, Fe, Zn, and Cu.10 Populations that have traditionally eaten a heavy plant-based diet are at risk for which diseases/illnesses? Which individuals in society are most vulnerable to micronutrient deficiencies?
Diseases:
Fe: anemia, low birth weight, reduced learning capacity
Zn: poor pregnancy outcomes, decrease resistance to infectious diseases
Vulnerable individuals10
Pregnant women and children
Poor families.

## Slide 40
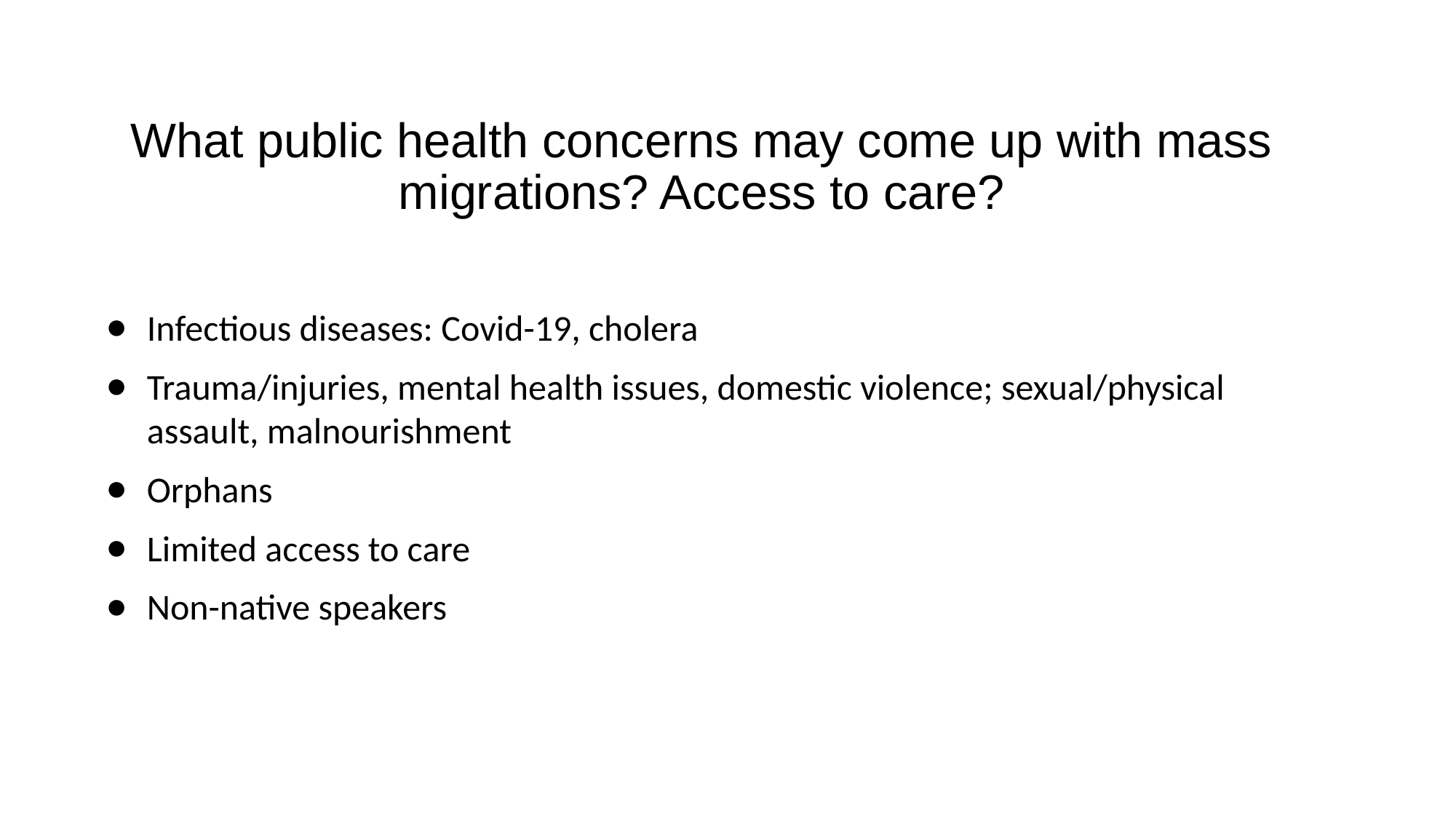

What public health concerns may come up with mass migrations? Access to care?
Infectious diseases: Covid-19, cholera
Trauma/injuries, mental health issues, domestic violence; sexual/physical assault, malnourishment
Orphans
Limited access to care
Non-native speakers

## Slide 41
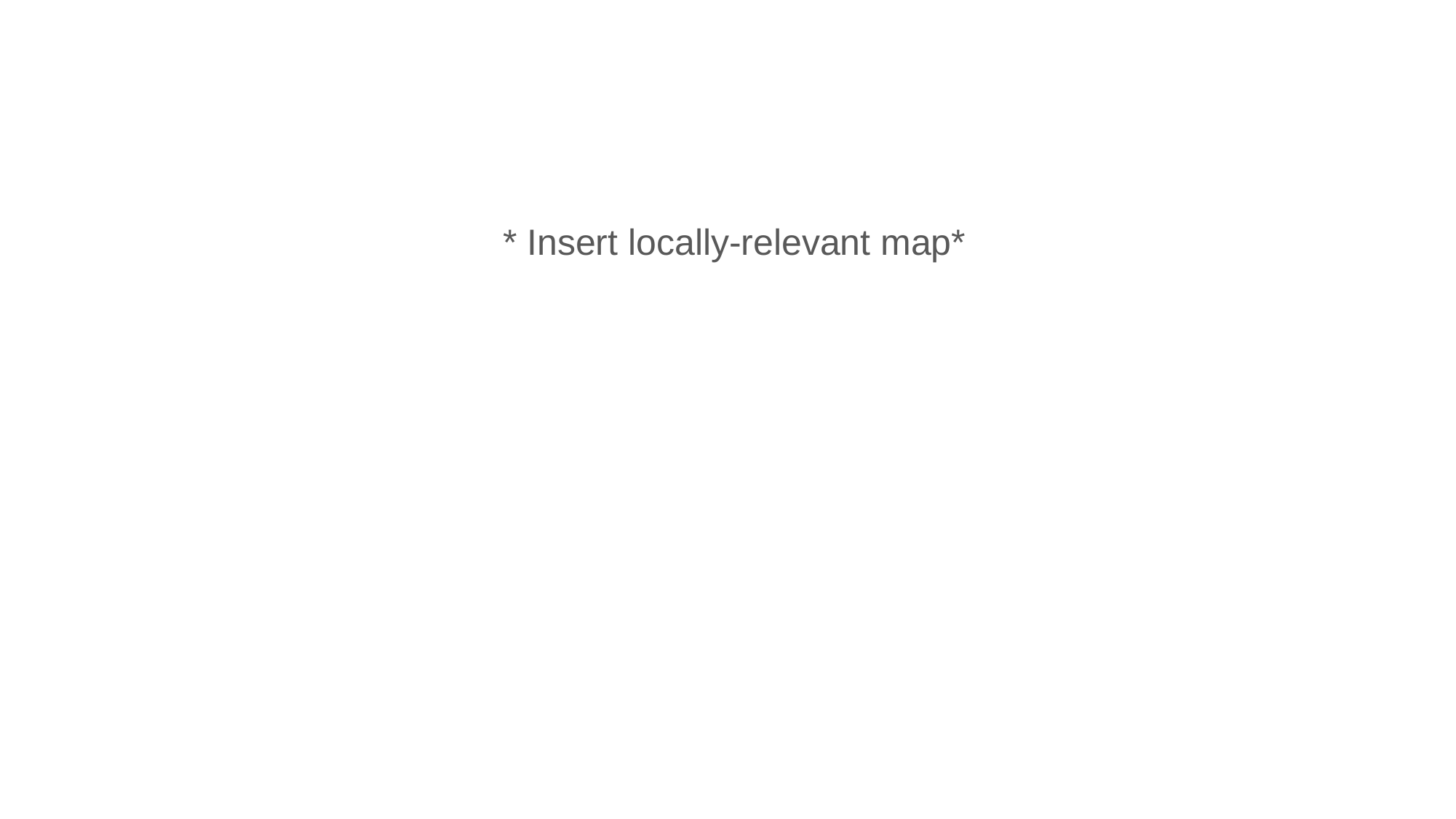

#
* Insert locally-relevant map*

## Slide 42
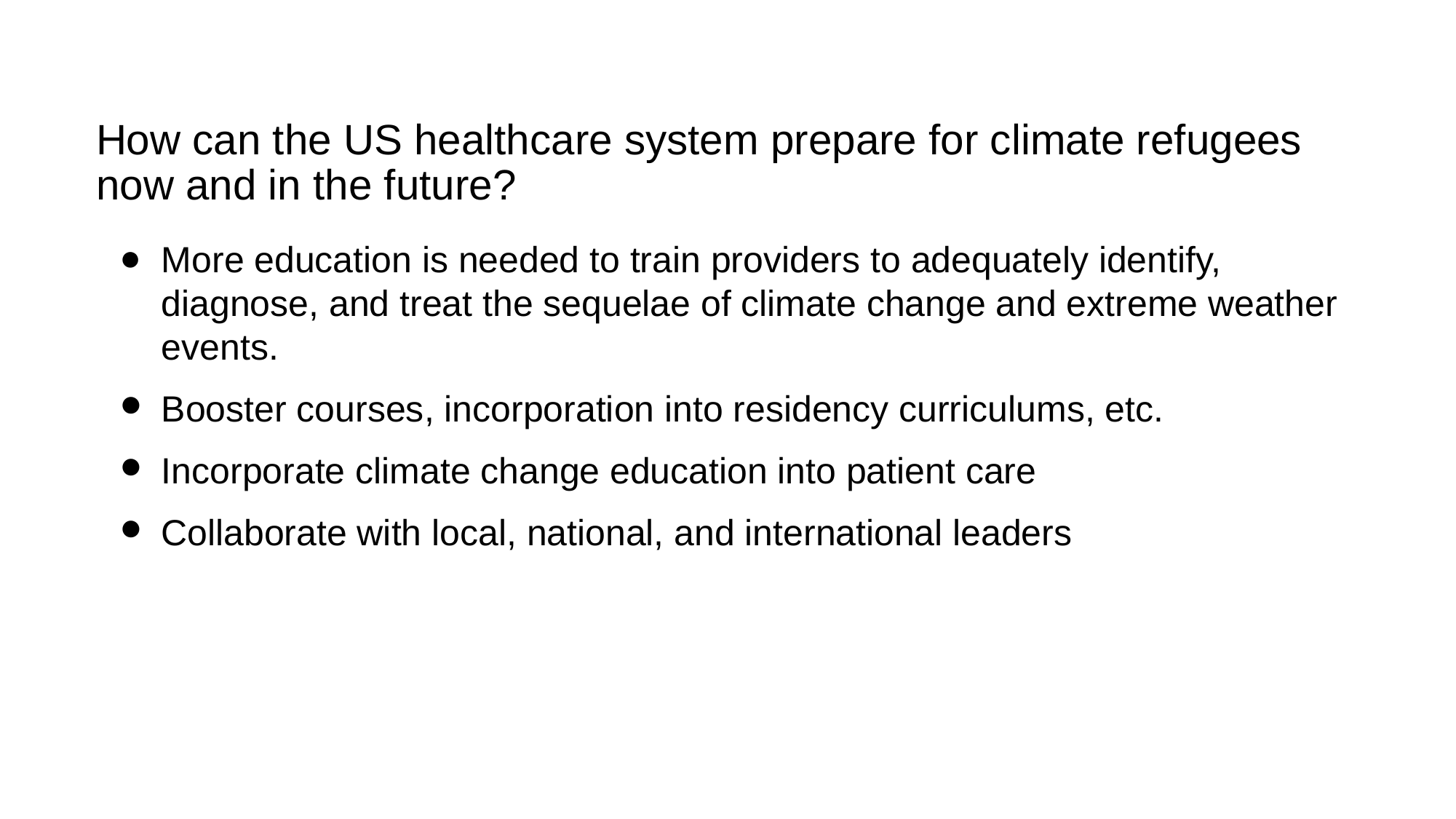

How can the US healthcare system prepare for climate refugees now and in the future?
More education is needed to train providers to adequately identify, diagnose, and treat the sequelae of climate change and extreme weather events.
Booster courses, incorporation into residency curriculums, etc.
Incorporate climate change education into patient care
Collaborate with local, national, and international leaders

## Slide 43
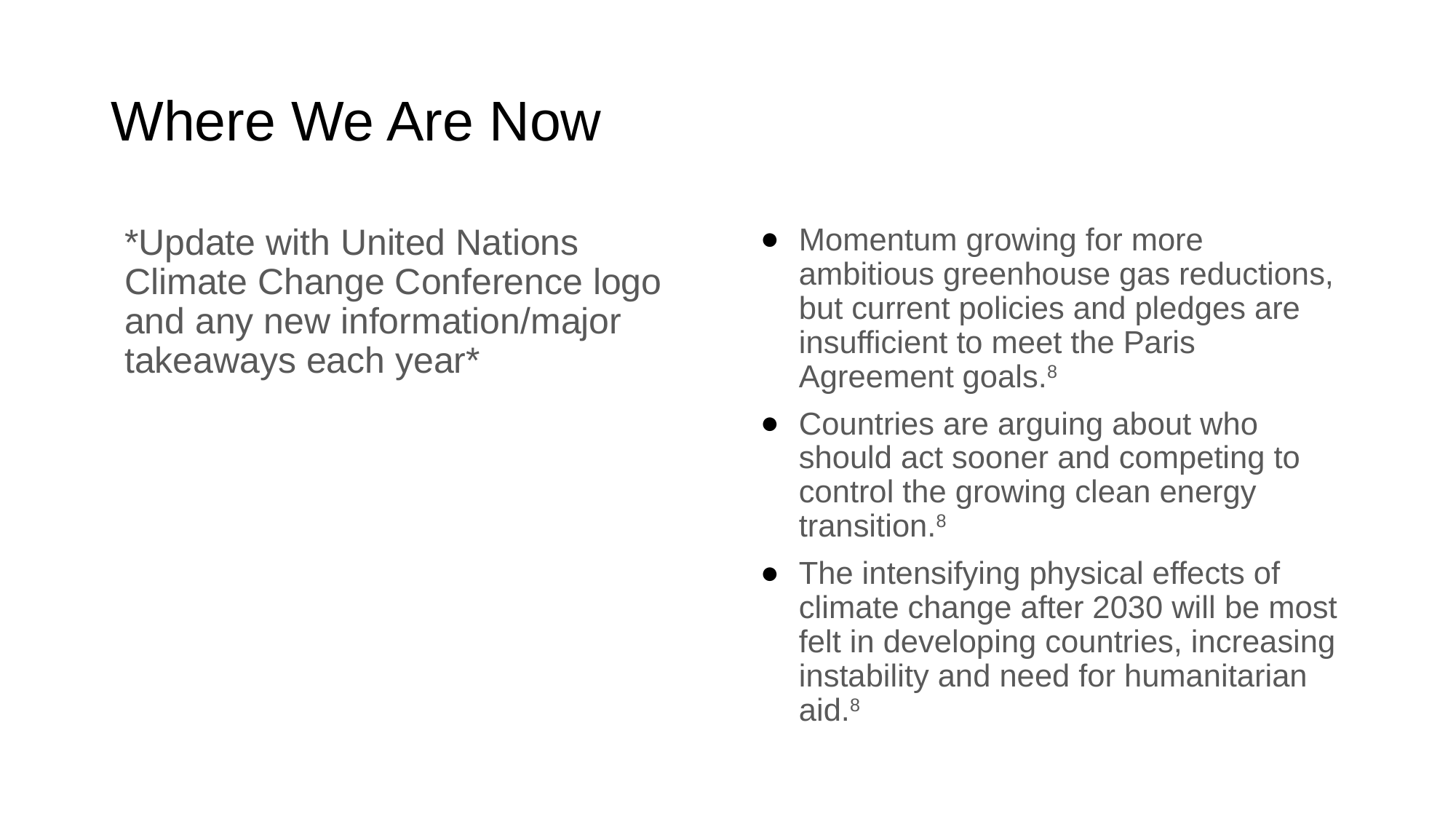

# Where We Are Now
*Update with United Nations Climate Change Conference logo and any new information/major takeaways each year*
Momentum growing for more ambitious greenhouse gas reductions, but current policies and pledges are insufficient to meet the Paris Agreement goals.8
Countries are arguing about who should act sooner and competing to control the growing clean energy transition.8
The intensifying physical effects of climate change after 2030 will be most felt in developing countries, increasing instability and need for humanitarian aid.8

## Slide 44
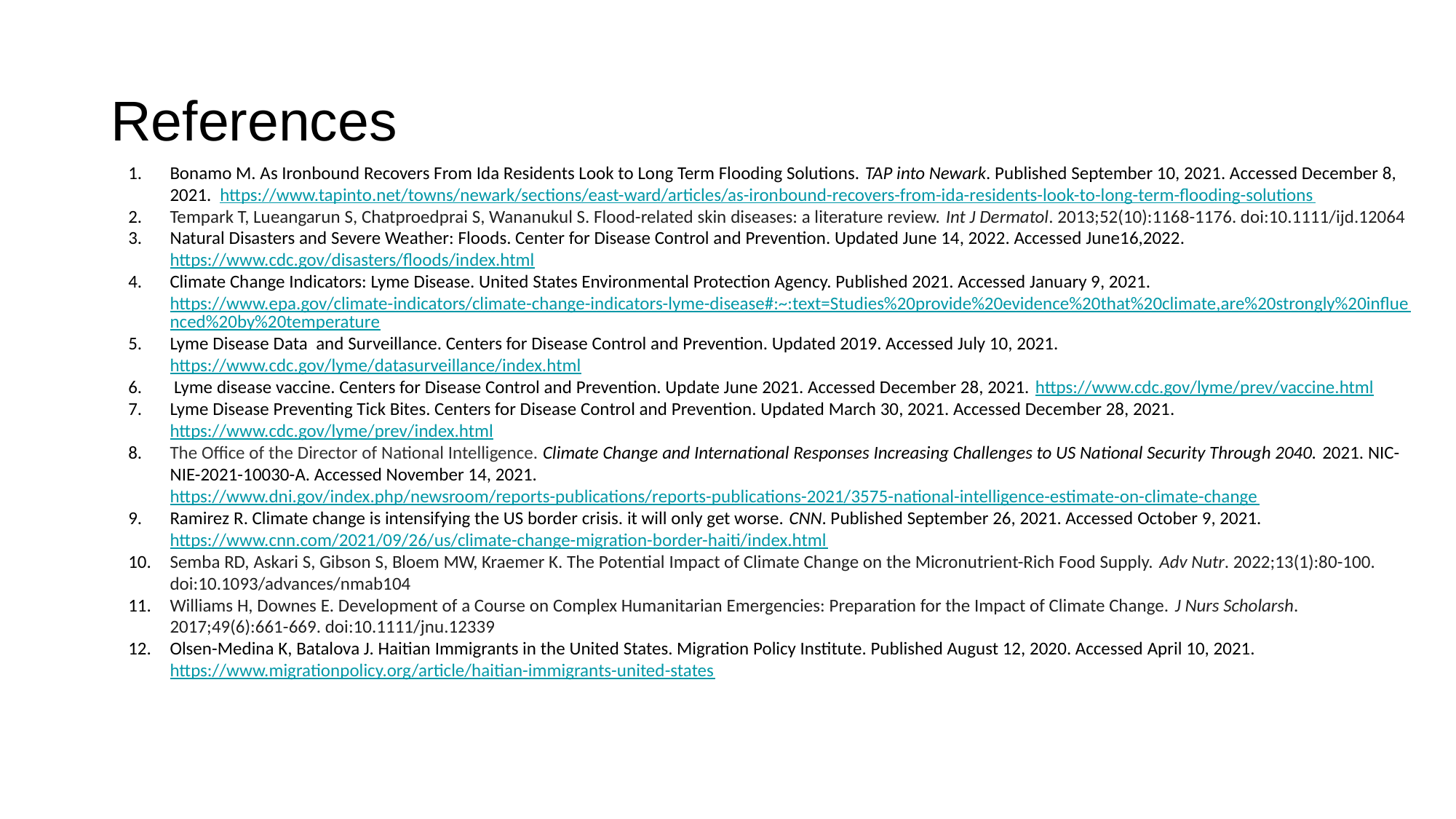

# References
Bonamo M. As Ironbound Recovers From Ida Residents Look to Long Term Flooding Solutions. TAP into Newark. Published September 10, 2021. Accessed December 8, 2021.  https://www.tapinto.net/towns/newark/sections/east-ward/articles/as-ironbound-recovers-from-ida-residents-look-to-long-term-flooding-solutions
Tempark T, Lueangarun S, Chatproedprai S, Wananukul S. Flood-related skin diseases: a literature review. Int J Dermatol. 2013;52(10):1168-1176. doi:10.1111/ijd.12064
Natural Disasters and Severe Weather: Floods. Center for Disease Control and Prevention. Updated June 14, 2022. Accessed June16,2022. https://www.cdc.gov/disasters/floods/index.html
Climate Change Indicators: Lyme Disease. United States Environmental Protection Agency. Published 2021. Accessed January 9, 2021. https://www.epa.gov/climate-indicators/climate-change-indicators-lyme-disease#:~:text=Studies%20provide%20evidence%20that%20climate,are%20strongly%20influenced%20by%20temperature
Lyme Disease Data and Surveillance. Centers for Disease Control and Prevention. Updated 2019. Accessed July 10, 2021. https://www.cdc.gov/lyme/datasurveillance/index.html
 Lyme disease vaccine. Centers for Disease Control and Prevention. Update June 2021. Accessed December 28, 2021. https://www.cdc.gov/lyme/prev/vaccine.html
Lyme Disease Preventing Tick Bites. Centers for Disease Control and Prevention. Updated March 30, 2021. Accessed December 28, 2021. https://www.cdc.gov/lyme/prev/index.html
The Office of the Director of National Intelligence. Climate Change and International Responses Increasing Challenges to US National Security Through 2040. 2021. NIC-NIE-2021-10030-A. Accessed November 14, 2021. https://www.dni.gov/index.php/newsroom/reports-publications/reports-publications-2021/3575-national-intelligence-estimate-on-climate-change
Ramirez R. Climate change is intensifying the US border crisis. it will only get worse. CNN. Published September 26, 2021. Accessed October 9, 2021. https://www.cnn.com/2021/09/26/us/climate-change-migration-border-haiti/index.html
Semba RD, Askari S, Gibson S, Bloem MW, Kraemer K. The Potential Impact of Climate Change on the Micronutrient-Rich Food Supply. Adv Nutr. 2022;13(1):80-100. doi:10.1093/advances/nmab104
Williams H, Downes E. Development of a Course on Complex Humanitarian Emergencies: Preparation for the Impact of Climate Change. J Nurs Scholarsh. 2017;49(6):661-669. doi:10.1111/jnu.12339
Olsen-Medina K, Batalova J. Haitian Immigrants in the United States. Migration Policy Institute. Published August 12, 2020. Accessed April 10, 2021. https://www.migrationpolicy.org/article/haitian-immigrants-united-states

## Slide 45
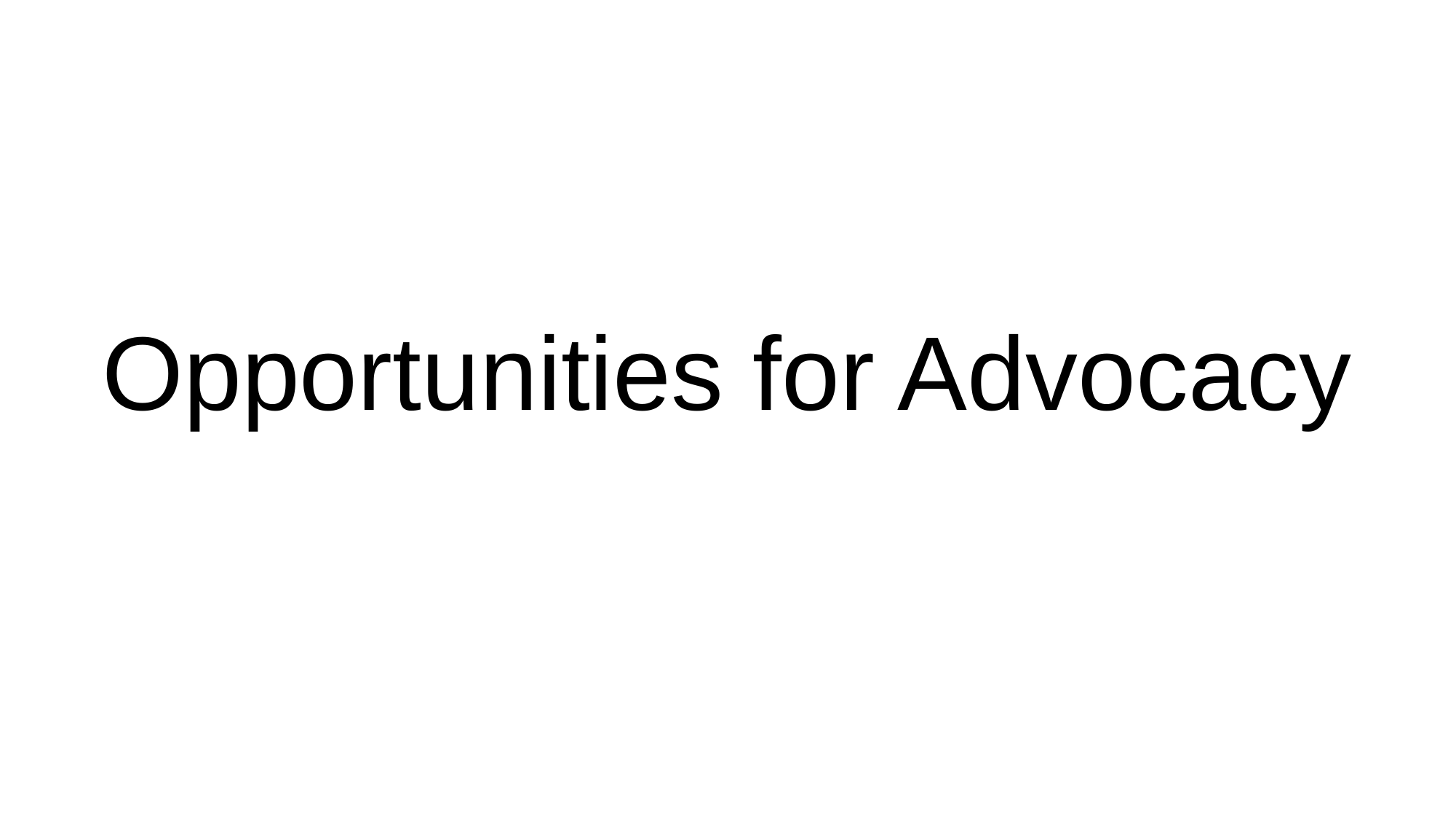

# Opportunities for Advocacy

## Slide 46
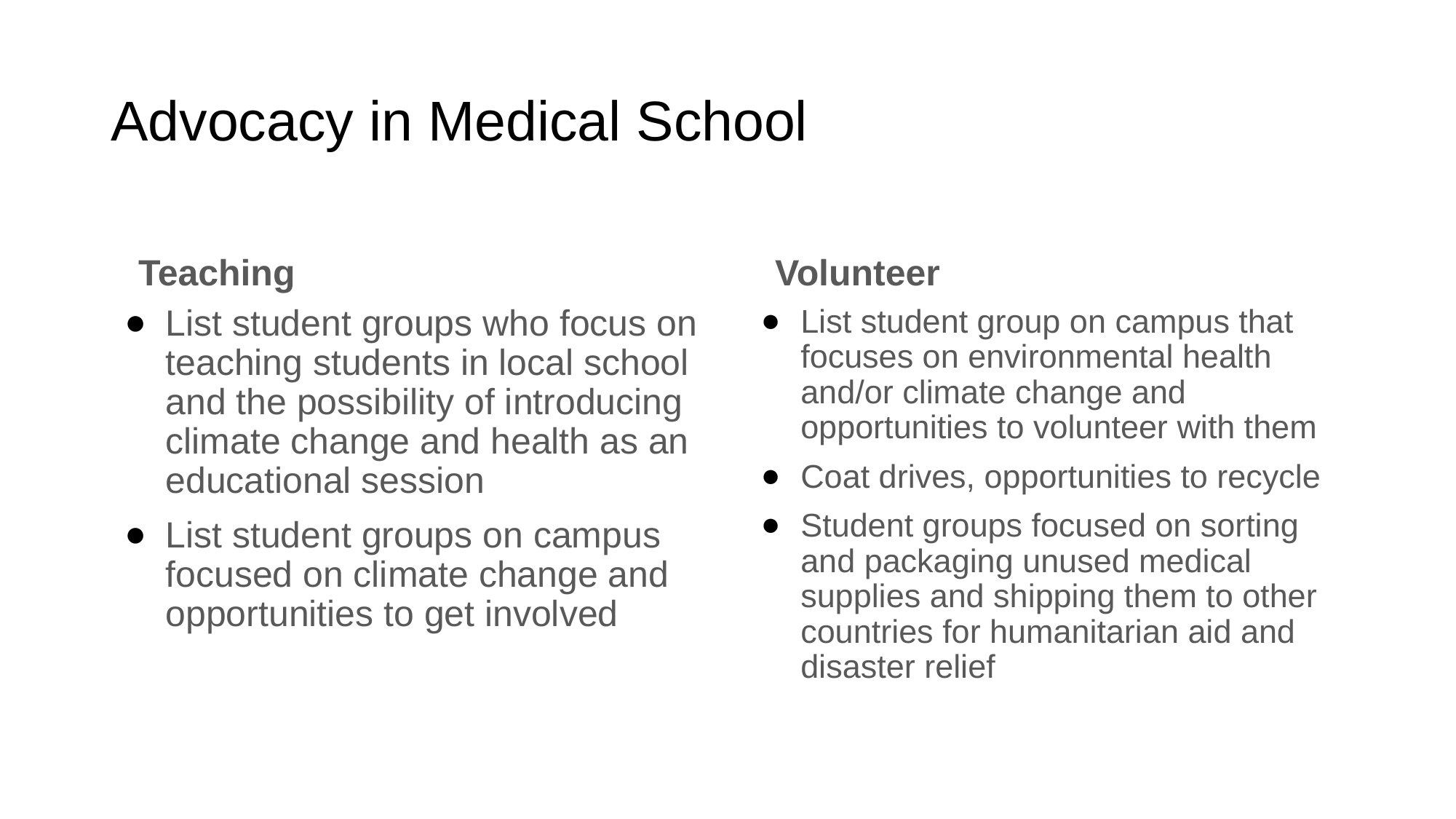

# Advocacy in Medical School
Teaching
Volunteer
List student groups who focus on teaching students in local school and the possibility of introducing climate change and health as an educational session
List student groups on campus focused on climate change and opportunities to get involved
List student group on campus that focuses on environmental health and/or climate change and opportunities to volunteer with them
Coat drives, opportunities to recycle
Student groups focused on sorting and packaging unused medical supplies and shipping them to other countries for humanitarian aid and disaster relief

## Slide 47
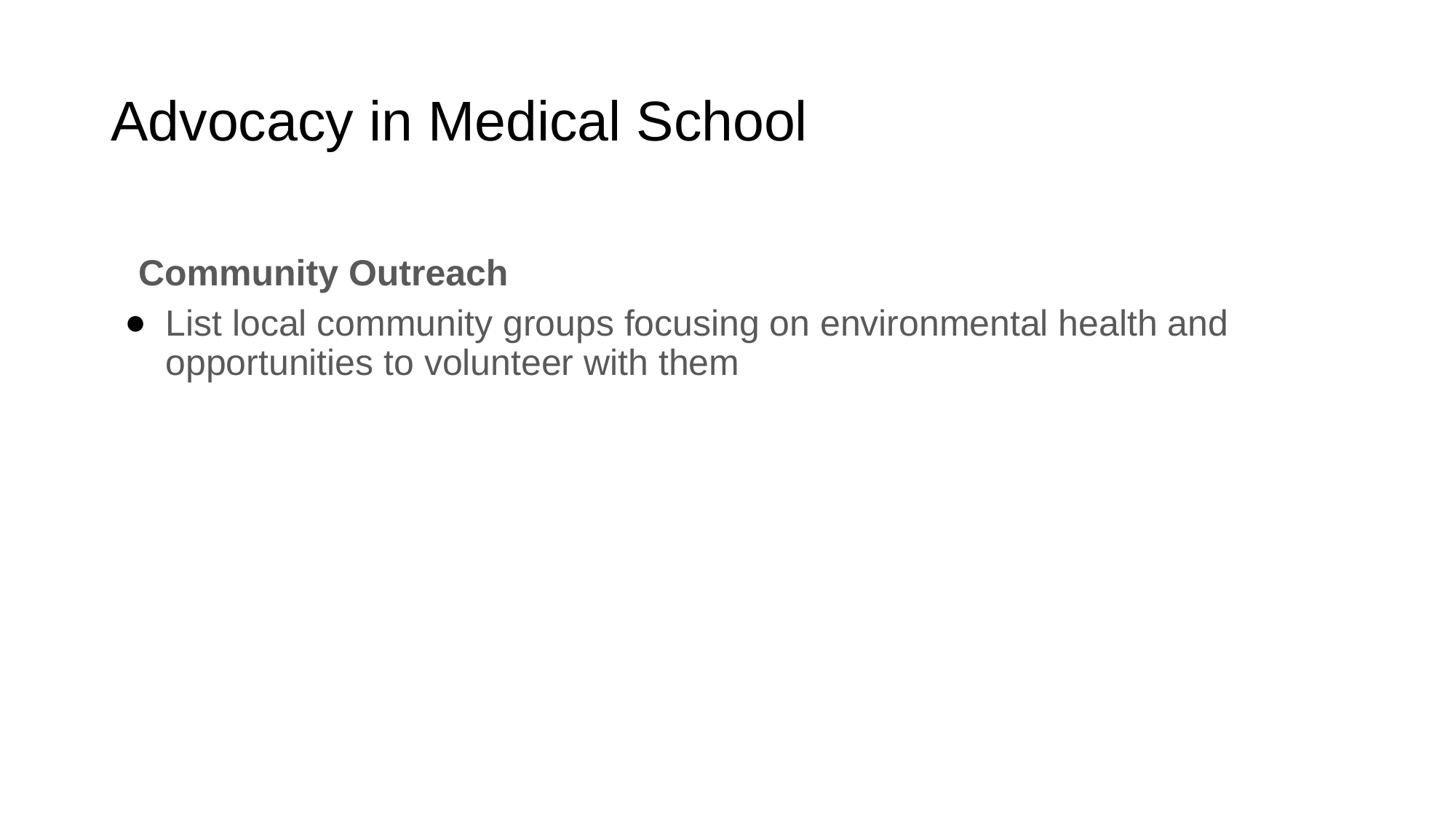

# Advocacy in Medical School
Community Outreach
List local community groups focusing on environmental health and opportunities to volunteer with them

## Slide 48
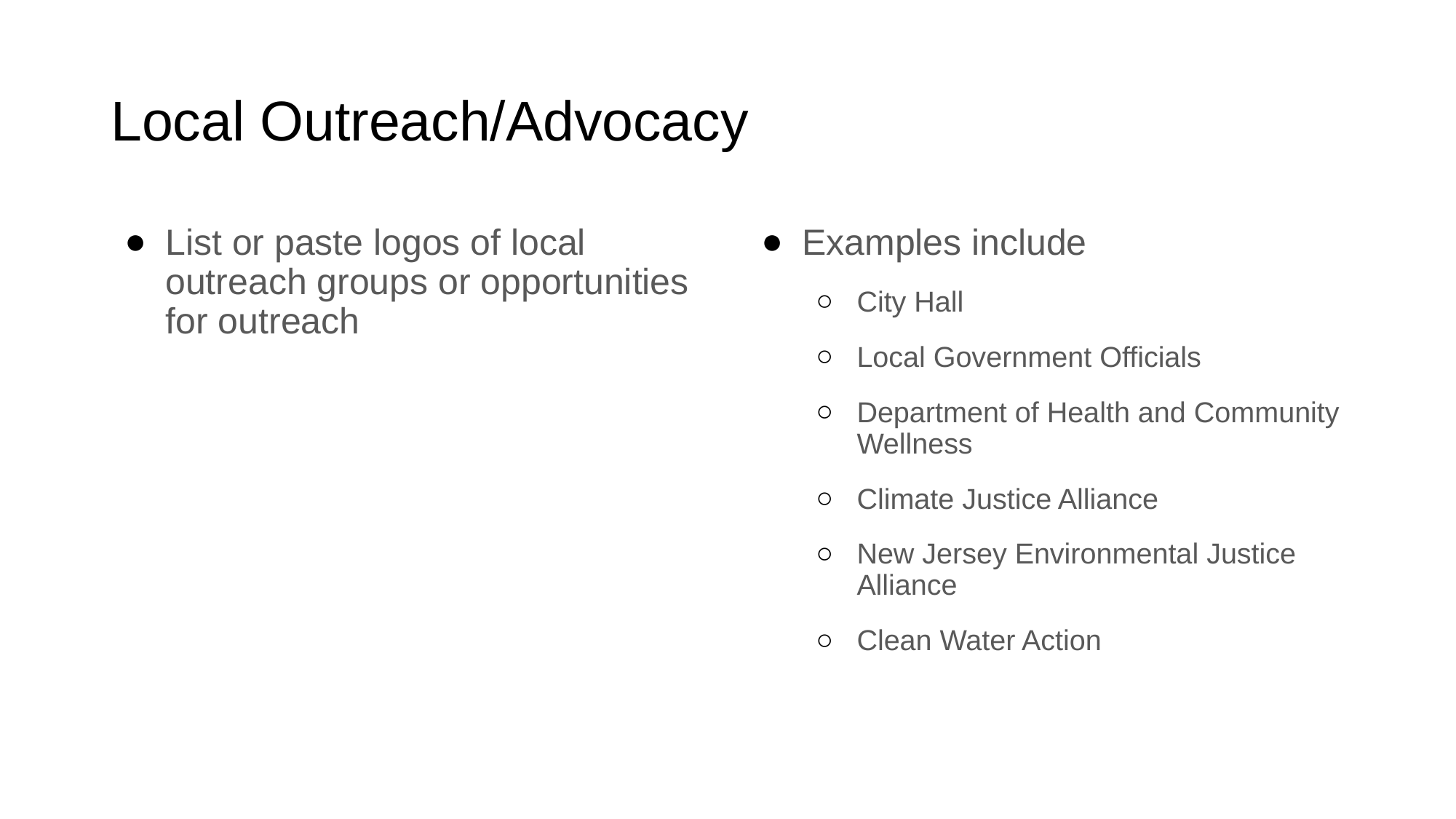

# Local Outreach/Advocacy
List or paste logos of local outreach groups or opportunities for outreach
Examples include
City Hall
Local Government Officials
Department of Health and Community Wellness
Climate Justice Alliance
New Jersey Environmental Justice Alliance
Clean Water Action

## Slide 49
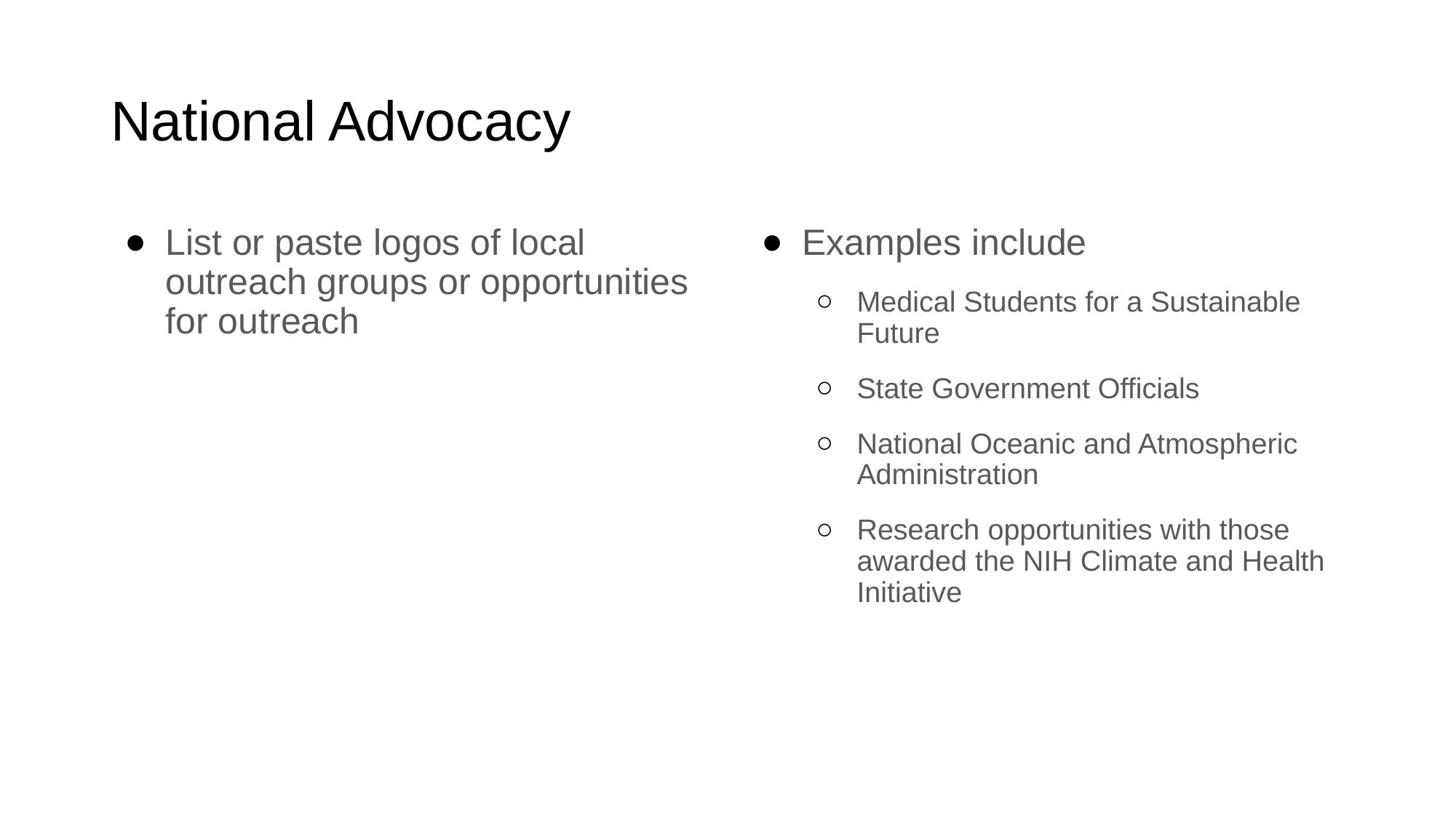

# National Advocacy
List or paste logos of local outreach groups or opportunities for outreach
Examples include
Medical Students for a Sustainable Future
State Government Officials
National Oceanic and Atmospheric Administration
Research opportunities with those awarded the NIH Climate and Health Initiative

## Slide 50
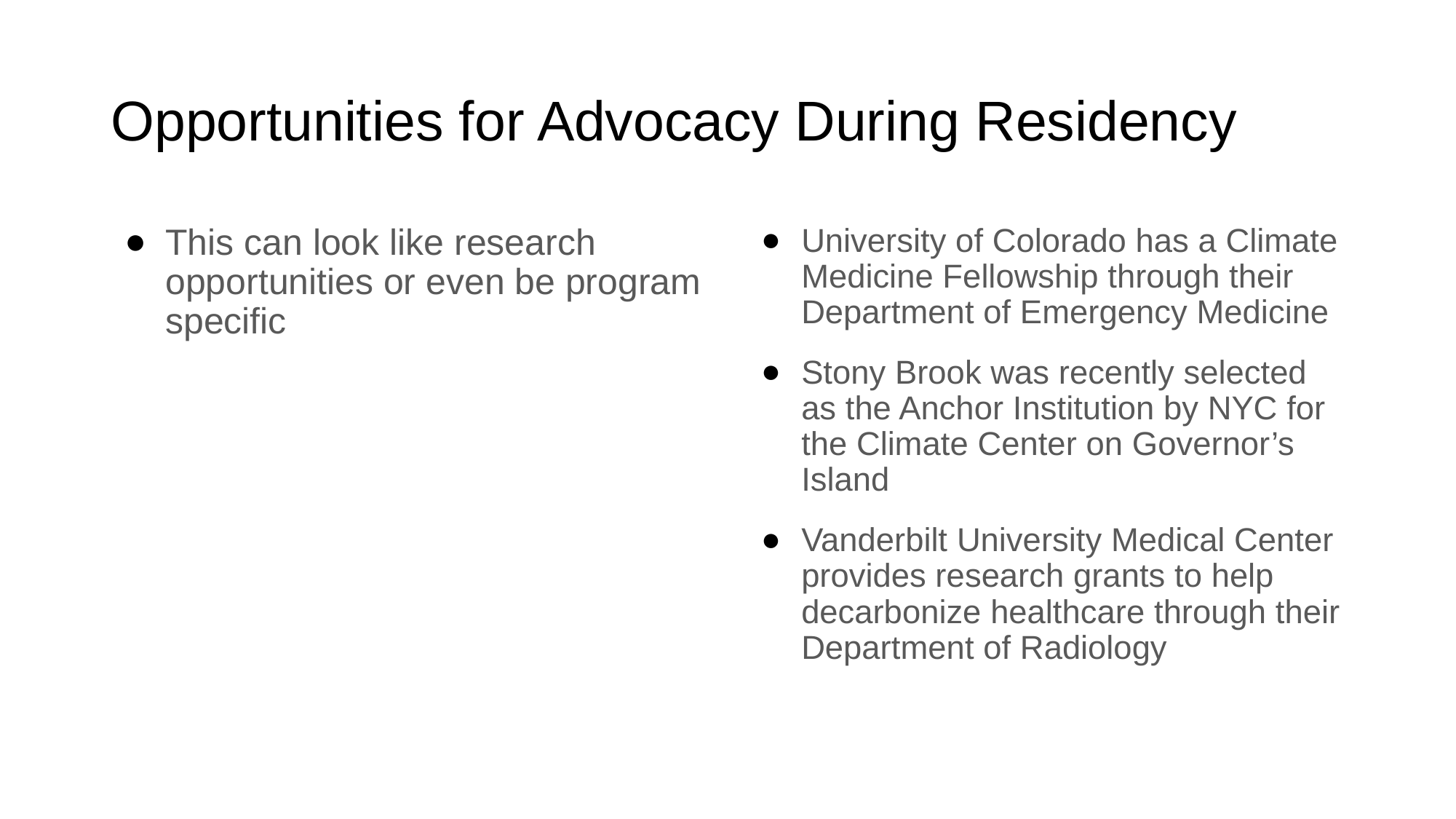

# Opportunities for Advocacy During Residency
This can look like research opportunities or even be program specific
University of Colorado has a Climate Medicine Fellowship through their Department of Emergency Medicine
Stony Brook was recently selected as the Anchor Institution by NYC for the Climate Center on Governor’s Island
Vanderbilt University Medical Center provides research grants to help decarbonize healthcare through their Department of Radiology

## Slide 51
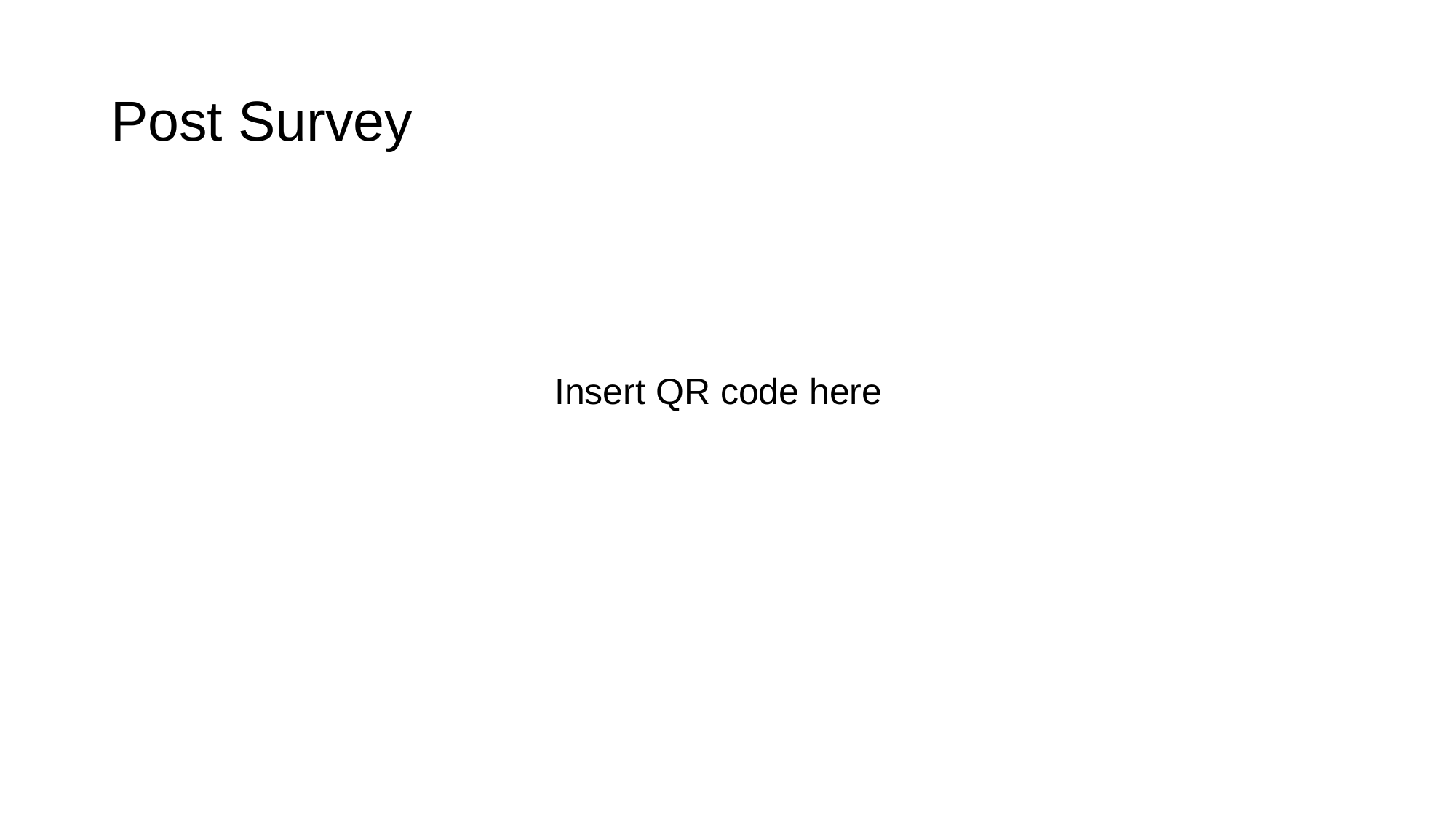

# Post Survey
Insert QR code here
