# Supplementary material for: An Interactive Mapping and Case Discussion Seminar Introducing Medical Students to Climate Change, Environmental Justice, and Health
Source: MedEdPORTAL. 2024 Apr 16;20:11398. doi: 10.15766/mep_2374-8265.11398 (PMC11018717; doi:10.15766/mep_2374-8265.11398)
Supplement: Supplementary file 1 — Didactic Lectures.pptxSmall-Group Student Handouts Example.docxSmall-Group Debriefs Example.pptxPre- and Postseminar Surveys.docxQuiz.docx [file mep_2374-8265.11398-s001.zip › D. Pre- and Postseminar Surveys.docx]

**Appendix D: Pre-and Post- Seminar Surveys**

***Instructions:*** *Students can be asked to complete the survey by allotting 5 minutes at the beginning and the end of the seminar. The survey can be administered using an electronic survey manager with a QR code or paper copies.*

**Pre-survey**

Rate the degree to which you agree that you can accomplish the following:

1. Explain the relationship between human activities and climate change.

| 1 | 2 | 3 | 4 | 5 |
| --- | --- | --- | --- | --- |
| Hardly at all | To a small degree | To a moderate degree | To a considerable degree | To a very high degree |

1. Describe the impact of climate change on socioeconomic systems and health, especially within the local context.

| 1 | 2 |  | 3 | 4 | 5 |
| --- | --- | --- | --- | --- | --- |
| Hardly at all | To a small degree |  | To a moderate degree | To a considerable degree | To a very high degree |

1. Describe clinical care strategies to address the health effects of climate change.

| 1 | 2 | 3 | 4 | 5 |
| --- | --- | --- | --- | --- |
| Hardly at all | To a small degree | To a moderate degree | To a considerable degree | To a very high degree |

1. Outline system-wide climate change mitigation strategies for healthcare systems.

| 1 | 2 | 3 | 4 | 5 |
| --- | --- | --- | --- | --- |
| Hardly at all | To a small degree | To a moderate degree | To a considerable degree | To a very high degree |

1. Integrate climate change advocacy as a component of health justice advocacy.

| 1 | 2 | 3 | 4 | 5 |
| --- | --- | --- | --- | --- |
| Hardly at all | To a small degree | To a moderate degree | To a considerable degree | To a very high degree |

1. Rate the degree to which you agree with the following statement: “The topic of climate change and health is important to learn in order to become an effective physician

| 1 | 2 | 3 | 4 | 5 |
| --- | --- | --- | --- | --- |
| Hardly at all | To a small degree | To a moderate degree | To a considerable degree | To a very high degree |

**Narrative Feedback**

List any topics about which you would like to learn more for this session.

**Post-seminar survey**

*Using the scale below, please evaluate this activity in terms of:*

1. Overall content

| 1 | 2 | 3 | 4 | 5 |
| --- | --- | --- | --- | --- |
| Needs significant improvement | Fair | Adequate | Good | Excellent |

1. Didactics/Lectures

| 1 | 2 | 3 | 4 | 5 |
| --- | --- | --- | --- | --- |
| Needs significant improvement | Fair | Adequate | Good | Excellent |

1. Effectiveness of the lecturer

| 1 | 2 | 3 | 4 | 5 |
| --- | --- | --- | --- | --- |
| Needs significant improvement | Fair | Adequate | Good | Excellent |

1. Interactive Map Activity

| 1 | 2 | 3 | 4 | 5 |
| --- | --- | --- | --- | --- |
| Needs significant improvement | Fair | Adequate | Good | Excellent |

1. Case Discussion Activity

| 1 | 2 | 3 | 4 | 5 |
| --- | --- | --- | --- | --- |
| Needs significant improvement | Fair | Adequate | Good | Excellent |

*Rate the degree to which you agree that you can accomplish the following:*

1. Explain the relationship between human activities and climate change.

| 1 | 2 | 3 | 4 | 5 |
| --- | --- | --- | --- | --- |
| Hardly at all | To a small degree | To a moderate degree | To a considerable degree | To a very high degree |

1. Describe the impact of climate change on socioeconomic systems and health, especially within the local context.

| 1 | 2 | 3 | 4 | 5 |
| --- | --- | --- | --- | --- |
| Hardly at all | To a small degree | To a moderate degree | To a considerable degree | To a very high degree |

1. Describe clinical care strategies to address the health effects of climate change.

| 1 | 2 | 3 | 4 | 5 |
| --- | --- | --- | --- | --- |
| Hardly at all | To a small degree | To a moderate degree | To a considerable degree | To a very high degree |

1. Outline system-wide climate change mitigation strategies for healthcare systems.

| 1 | 2 | 3 | 4 | 5 |
| --- | --- | --- | --- | --- |
| Hardly at all | To a small degree | To a moderate degree | To a considerable degree | To a very high degree |

1. Integrate climate change advocacy as a component of health justice advocacy.

| 1 | 2 | 3 | 4 | 5 |
| --- | --- | --- | --- | --- |
| Hardly at all | To a small degree | To a moderate degree | To a considerable degree | To a very high degree |

1. *Rate the degree to which you agree with the following statement:* “The topic of climate change and health is important to learn in order to become an effective physician

| 1 | 2 | 3 | 4 | 5 |
| --- | --- | --- | --- | --- |
| Hardly at all | To a small degree | To a moderate degree | To a considerable degree | To a very high degree |

1. *Rate the degree to which you agree with the following statement:* “I plan to apply what I have learned to the practice of medicine in the future.”

| 1 | 2 | 3 | 4 | 5 |
| --- | --- | --- | --- | --- |
| Hardly at all | To a small degree | To a moderate degree | To a considerable degree | To a very high degree |

**Narrative Feedback**

1. Please comment on the strengths of this activity.
2. Please include your suggestions for how this activity can be improved.
