# Supplementary material for: An Interactive Mapping and Case Discussion Seminar Introducing Medical Students to Climate Change, Environmental Justice, and Health
Source: MedEdPORTAL. 2024 Apr 16;20:11398. doi: 10.15766/mep_2374-8265.11398 (PMC11018717; doi:10.15766/mep_2374-8265.11398)
Supplement: Supplementary file 1 — Didactic Lectures.pptxSmall-Group Student Handouts Example.docxSmall-Group Debriefs Example.pptxPre- and Postseminar Surveys.docxQuiz.docx [file mep_2374-8265.11398-s001.zip › _Educational Summary Report_11398.pdf]

MedEdPORTAL® | The AAMC Journal of Teaching and Learning Resources

Original Publication

Open Access

An Interactive Mapping and Case Discussion Seminar Introducing Medical Students to Climate Change, Environmental Justice, and Health

Victoria Ribeiro, Evan Grossi, MD, Yaxel Levin-Carrion, Novneet Sahu, MD, Michelle DallaPiazza, MD*

*Corresponding author: mld229@njms.rutgers.edu

Abstract

Introduction: Integrating climate change and health into a medical school curriculum is critical for future physicians who will manage health crises caused by a rapidly changing climate. Although medical schools have increasingly included climate change in the curriculum, there remains a need to address the link between the climate crisis, environmental justice, and historical policies that shape environmental health disparities in local communities. **Methods:** In academic years 2021-2022 (AY22) and 2022-2023 (AY23), second-year medical students participated in a 2.5-hour seminar utilizing didactic teaching and small breakout groups that included interactive mapping activities and case scenarios. Learner knowledge and attitudes were self-assessed using pre- and postcurriculum surveys and a quiz. Qualitative thematic and content analysis was used to evaluate short-answer quiz responses and feedback. **Results:** Of 357 students who participated in the seminar, 208 (58%) completed both the precurriculum and postcurriculum surveys. Self-assessed ability increased significantly for all educational objectives across both years. Attitudes on the importance of climate change knowledge for patient health also improved from a mean of 3.5 precurriculum to 4.2 postcurriculum (difference = 0.7, $p < .01$) in AY22 and from 3.6 pre- to 4.3 postcurriculum (difference = 0.7, $p < .01$) in AY23 on a 5-point Likert scale. **Discussion:** This climate change and health session highlighting the link between environmental policy and climate change health vulnerability in the local context was successful in improving students' self-assessed ability across all stated educational objectives. Students cited the interactive small-group sessions as a major strength.

Keywords
Environmental Justice, Health Equity, Emergency Medicine, Case-Based Learning, Problem-Based Learning, Climate Change, Editor's Choice

Educational Objectives

By the end of this activity, learners will be able to:

1. Explain the relationship between human activities and climate change.
2. Describe the impact of climate change on socioeconomic systems and health, especially within the local context.
3. Describe clinical care strategies to address the health effects of climate change.
4. Outline systemwide climate change mitigation strategies for health care systems.
5. Integrate climate change advocacy as a component of health justice advocacy.

Citation:
Ribeiro V, Grossi E, Levin-Carrion Y, Sahu N, DallaPiazza M. An interactive mapping and case discussion seminar introducing medical students to climate change, environmental justice, and health. *MedEdPORTAL*. 2024;20:11398.
https://doi.org/10.15766/mep_2374-8265.11398

Introduction

When the World Health Organization declared climate change the "single biggest threat facing humanity,"[1] the American Medical Association responded by passing a policy supporting the integration of climate change and its health implications across all levels of medical education.[2] Despite the wide-ranging impacts of the changing climate on human health—including infectious disease outbreaks, chronic disease exacerbation, population displacement, and food insecurity—a recently published survey of 722 US medical students from 24 medical schools demonstrated that while 84% of students felt it important to include in medical school curricula, only 13% believed that their school provided adequate education on this topic.[3]

Prior work on climate change education in the preclerkship years has primarily included incorporating climate crisis slide decks into

Copyright © 2024 Ribeiro et al. This is an open-access publication distributed under the terms of the Creative Commons Attribution license.

existing lectures[4] and participating in elective courses.[4,5] Among required modules, the Hackensack Meridian School of Medicine has integrated climate change topics into its environmental health module, which is part of its required 3-year Human Dimension course,[6] and the Baylor College of Medicine recently described in *MedEdPORTAL* a mandatory 1-hour module for all first-year students.[7] Other programs have opted to integrate climate change education into the clerkship curriculum, including clinical electives,[8,9] online courses,[10] seminar series,[11] and simulated standardized patient encounters.[12] Much of the prior work has focused on climate change's strain on health care, health impacts, and clinical management. However, the link between the climate crisis, environmental justice, and historical policies shaping health disparities in local communities remains an area for further educational development. Furthermore, ensuring that climate change and health instruction is a required part of the medical school curriculum is essential for preparing future physicians to manage climate-related chronic illnesses and health crises. To address these gaps, we created a required, interactive, 2.5-hour seminar for second-year preclerkship medical students.

Using a collaborative approach across the medical education continuum, the curriculum development team consisted of a student champion (Victoria Ribeiro), a resident champion (Evan Grossi), and two faculty members (Novneet Sahu and Michelle DallaPiazza). This seminar was part of a medical education distinction project for the student champion, who played a key role in curriculum design and implementation under the guidance of the faculty. We chose the instructional method of integrating short didactics with mapping exercises and case-based discussions to efficiently convey foundational knowledge while also employing adult problem-based learning principles.[13] In designing the content to be locally relevant, we aimed to concretize learning for second-year students who would soon see the effect of climate change on the patient populations of New Jersey in their clinical clerkships.

In the academic year 2021-2022 (AY22,) we introduced this seminar within the longitudinal Health Equity and Social Justice (HESJ) thread at Rutgers New Jersey Medical School. HESJ spans the preclerkship years and includes content exploring structural competency and population health.[14] Specifically designed for a US-based audience, the seminar aimed to introduce the impact of climate change on health within the local context of Newark and New Jersey, as well as to provide guidance for integrating an environmental justice-based approach in patient care. This report summarizes the educational outcomes observed for the first 2 years, AY22 and 2022-2023 (AY23).

Methods

Structure and Timing of the Seminar

Appendix A includes a summary of the structure and timing of seminar activities. All activities used a virtual, video-based platform in AY22 due to a surge in the COVID pandemic, and in AY23, all activities were delivered in person. For the small-group activities, we used virtual breakout rooms in AY22 and a small-group format in a large lecture hall in AY23.

Recommended Background Resources

Prior to the session, we recommended that students read Renee N. Salas's perspective article "Environmental Racism and Climate Change—Missed Diagnoses,"[15] which described a patient story illustrating the relationship between policies such as redlining, community disinvestment, and adverse environmental exposures resulting from climate change. The article also discussed strategies for incorporating diagnostic and treatment considerations into clinical practice through the lens of climate change.

Didactic Lecture 1: Climate Change and Population Health

The first large-group didactic lecture (Appendix A) focused on important definitions and general principles for climate change, its effects on human health, the impact of extreme weather on infrastructure and health care, and how the climate crisis is currently affecting the local environment (Newark, New Jersey). Specific examples included urban heat islands, climate disasters and disrupted infrastructure, and the impact of climate disasters on health care delivery.

Interactive Mapping Exercise

Students were then randomly assigned into virtual breakout groups (AY22) or asked to turn to the people sitting next to them (AY23) in groups of five to six students and asked to examine one of three maps of Newark with a focus on (1) air pollution and asthma, (2) redlining and urban heat islands, and (3) flooding and housing insecurity. Each third of the large group was assigned one of the three maps. Maps were selected based on points emphasized during the lecture, environmental and health issues the city was experiencing, and availability of interactive maps. Students received links to the maps and discussion questions on detailed worksheets (Appendix B) for the 20-minute breakouts. The discussion questions were developed to highlight the relationship between environmental inequities, risk factors, and

Copyright © 2024 Ribeiro et al. This is an open-access publication distributed under the terms of the Creative Commons Attribution license.

historical policies contributing to population health issues across different city areas. After the small groups, the students returned to the large group for a 10-minute facilitated discussion of their findings (Appendix C). Both Appendices B and C are meant to be used as guides for creating customized maps and cases for the local context and provide the materials used for this session as examples.

Didactic Lecture 2: Climate Change and Health Systems

The second large-group lecture explored the medical workup through a climate change lens, the health care system's contribution to climate change, and mitigation strategies (Appendix A).

Case-Based Discussions

Similar to the map-based activity, the 20-minute small group that followed consisted of three case studies showing different ways climate change could present in a clinical setting (Appendix B). The cases explored (1) flooding, contaminated water, and health care access after a climate event; (2) the changing epidemiology of Lyme disease as influenced by climate change; and (3) the medical and public health impact of climate migrants. Each case was based on events and/or trends observed in northern New Jersey. After the case discussions, students returned to the large group for a 10-minute facilitated discussion of their findings (Appendix C).

Evaluation

Before and after the seminar, students completed anonymous surveys linked with an anonymous identifier to evaluate the effectiveness of the teaching (Appendix D). Self-assessed ability in achieving the stated Educational Objectives, attitudes on climate change and health, and satisfaction with seminar components were measured using a 5-point Likert scale (*1 = hardly at all, 5 = to a very high degree*). The survey also provided space for narrative feedback.

Assessment

Student knowledge assessment consisted of an open-content online quiz (Appendix E) testing content from the lectures and the small groups, due 1 week after the session. The course director developed and graded the quiz, which included five multiple-choice and two short-answer essay questions. Quiz scores were incorporated into the overall grade for the HESJ course. The short-answer responses were graded based on demonstrating (1) understanding of the content, (2) thoughtfulness and self-reflection in the responses, and (3) evidence of having engaged with the topics of discussion and resources (see the grading rubric in Appendix E). Students were given these criteria in advance in the course syllabus and during the course introduction.

Data Analysis

Data analysis was performed using Excel. All students who completed the pre- and postsurveys were included in the knowledge and attitudes analysis. We compared mean composite Likert scores for knowledge and attitudes pre- and postcurriculum using paired Student *t* tests. Unpaired *t* tests were used to compare between AY22 and AY23. All *t* tests were two-sided, and $p < .05$ was considered statistically significant. We also used *t* tests to assess associations between student demographics and personal experience with climate change and survey responses. We analyzed content and themes from feedback sections and the students' text answers for the short-answer questions on the online quiz.[16] Quiz responses were downloaded from the online educational platform, and student identifiers (names) were permanently removed. Authors Victoria Ribeiro and Michelle DallaPiazza independently categorized, coded, and quantified the themes expressed in each response using NVivo. Discrepancies were resolved by consensus. We were granted institutional IRB approval to analyze evaluation and quiz data.

Results

Over the 2 academic years, 357 students participated in the seminar, 179 in AY22 and 178 in AY23. On the presurveys ($n = 290$), student-reported demographics and previous experiences with climate change were similar across both years (Table 1).

Matched Pre- and Postsurveys

Anonymous identifier-matched pre- and postsurveys were submitted by 208 of students (58%). In both AY22 ($n = 92$) and AY23 ($n = 116$), there were significant increases in self-assessed ability to achieve all the Educational Objectives, with the largest improvements related to the health equity and health care system mitigation strategies objectives (Table 2). Student attitudes on "climate change is an important topic to learn in order to become an effective physician" also showed an increase in AY22 (pre $M = 3.5$, post $M = 4.2$, difference $= 0.7$, $p < .01$) and AY23 (pre $M = 3.6$, post $M = 4.3$, difference $= 0.7$, $p < .01$). There was no significant association between prior climate change experiences and change in self-assessed Educational Objectives or for how students rated the importance of learning about the topic of climate change and health.

In the unpaired *t*-test analysis comparing the 2 years (AY22 virtual, AY23 in person), there was a significant difference in self-assessment of Educational Objectives 2-5, all of which showed a

Copyright © 2024 Ribeiro et al. This is an open-access publication distributed under the terms of the Creative Commons Attribution license.

Table 1. Student Demographics for AY22 ($N = 141$) and AY23 ($N = 149$)

Characteristic	AY22 No. (%)	AY23 No. (%)
Gender		
Man	63 (45)	76 (51)
Woman	74 (52)	70 (47)
Transgender or gender diverse	0 (0)	0 (0)
Chose not to answer	4 (3)	3 (2)
Race/ethnicity		
American Indian/Alaska Native	0 (0)	0 (0)
Asian (East Asian, South Asian, Southeast Asian, Native Hawaiian/Pacific Islander)	62 (44)	61 (41)
Black/African American	12 (9)	14 (9)
Hispanic/Latino/x	11 (8)	12 (8)
Middle Eastern/North African	0 (0)	0 (0)
Multiethnicity/something else	15 (11)	22 (15)
White	6 (4)	6 (4)
Chose not to answer	35 (25)	34 (23)
Experience with climate change		
Answered yes to "I or someone in my immediate family has been directly impacted by a climate disaster."	64 (45)	65 (46)
Answered yes to "I or someone in my immediate family has a chronic medical condition that has/can be impacted by climate change."	43 (30)	49 (35)

Abbreviations: AY22, academic year 2021-2022; AY23, academic year 2022-2023.

greater increase for AY22 (Table 2), which was largely driven by higher levels at baseline on the presurvey for AY23. With respect to satisfaction with the seminar components, all components were rated as a mean of 4.0 or greater in the postsurveys for both years. Students from AY23 demonstrated slightly higher satisfaction with the overall content (AY22 $M = 4.0$, $SD = 0.7$; AY 23 $M = 4.1$, $SD = 0.8$; $p < .01$), and AY22 demonstrated higher satisfaction with the lectures (AY22 $M = 4.4$, $SD = 0.8$; AY23 $M = 4.1$, $SD = 0.8$; $p < .01$).

Quiz Response Thematic Analysis

The average scores for the open-online quizzes were 97% in AY22 and 98% in AY23. Content and thematic analysis of quiz essay responses is summarized in Table 3. Students frequently commented that while they already had some awareness of health themes associated with climate change, the cases and maps helped them concretize the information and provided specific examples (e.g., tick distribution and seasonal changes, micronutrient deficiencies and food insecurity) that they had not previously considered.

Narrative Feedback

Students appreciated the relevance of the topic and the small-group components. Suggestions for improvement focused on covering more advocacy points and allotting less time to the small-group components (Table 4).

Discussion

Because the health effects of a rapidly changing climate are wide-ranging and increasingly evident, education for health professionals on clinical care through a climate justice lens is paramount. This interactive session consisting of short didactic lectures, interactive mapping activities, and case-based learning improved students' self-assessed knowledge and attitudes related to the stated Educational Objectives. Thematic

Table 2. Analysis of Matched Pre- and Postsurvey Learning Objective Confidence Levels for AY22 and AY23

	AY22 ($n = 92$)				AY23 ($n = 116$)				
Objective[a]	Presurvey *M* (*SD*)	Postsurvey *M* (*SD*)	*M* Difference (95% CI)	*p*	Presurvey *M* (*SD*)	Postsurvey *M* (*SD*)	*M* Difference (95% CI)	*p*	AY22, AY23 Difference: *p*[b]
1. Explain the relationship between human activities and climate change.	3.4 (1.0)	4.2 (0.7)	0.8 (0.6-1.0)	<.01	3.4 (1.1)	4.1 (0.7)	0.7 (0.5-0.9)	<.01	.55
2. Describe the impact of climate change on socioeconomic systems and health, especially within the local context.	3.0 (1.0)	4.3 (0.7)	1.3 (1.1-1.5)	<.01	3.2 (1.0)	4.2 (0.7)	1.0 (0.8-1.2)	<.01	.02
3. Describe clinical care strategies to address the health effects of climate change.	2.7 (1.0)	4.2 (0.8)	1.5 (1.3-1.8)	<.01	3.0 (1.1)	4.1 (0.7)	1.1 (1.0-1.4)	<.01	.02
4. Outline systemwide climate change mitigation strategies for health care systems.	2.4 (0.9)	4.1 (0.8)	1.7 (1.5-2.0)	<.01	2.7 (1.2)	4.0 (0.7)	1.3 (1.1-1.5)	<.01	<.01
5. Integrate climate change advocacy as a component of health justice advocacy.	2.5 (1.0)	4.1 (0.8)	1.6 (1.4-1.9)	<.01	2.8 (1.1)	4.1 (0.7)	1.3 (1.1-1.5)	<.01	.03

Abbreviations: AY22, academic year 2021-2022; AY23, academic year 2022-2023.
[a]Rated on a 5-point Likert scale (1 = *hardly at all*, 5 = *to a very high degree*).
[b]Comparative analysis of yearly differences between AY22 and AY23.

Copyright © 2024 Ribeiro et al. This is an open-access publication distributed under the terms of the Creative Commons Attribution license.

Table 3. Thematic and Content Analysis of Quiz Essay Responses for AY22 (*N* = 178) and AY23 (*N* = 179)

Theme[a]	AY22 No. (%)	AY23 No. (%)
Medical history: Of the aspects of the comprehensive medical history presented in the lecture, which stood out to you as particularly important when considering the effect of climate change on a patient's health and why?		
History of present illness		
Patient perspective on illness presentation	11 (6)	17 (10)
Past medical history		
Exacerbation of chronic illness (e.g., asthma)	53 (30)	60 (34)
Social history		
Exposure to pollutants, toxins	58 (33)	52 (29)
Vulnerability to UHI, natural disasters	56 (32)	39 (22)
Difficulty coping with the impacts of climate change (e.g., disability, socioeconomic status)	35 (20)	37 (21)
Review of systems		
Impact on body systems	1 (1)	3 (2)
Physical exam		
Acute presentation of illnesses (e.g., lung function)	1 (1)	3 (2)
Assessment and plan		
Impact on health infrastructure and ability to provide care	5 (3)	1 (1)
Providing patients with education, tools, and resources	15 (8)	30 (17)
Recognizing atypical presentations of disease/illness	21 (12)	9 (5)
Cases: Of the topics discussed in the case scenarios, what stood out to you as particularly important or surprising and why?		
Redlining and UHI		
UHI definition	16 (9)	11 (6)
UHI effect on medications	3 (2)	2 (1)
UHI as a result of environmental injustice	9 (5)	3 (2)
Pollution and asthma		
Air pollution and respiratory diseases	30 (17)	10 (6)
Hospitals as major sources of pollution	17 (10)	7 (4)
Flooding		
Inability to access/provide care	13 (7)	34 (19)
Housing insecurity	13 (7)	16 (9)
Increased risk of infections	33 (19)	51 (28)
Climate refugees		
Definition of a climate refugee	21 (12)	23 (13)
Mass migrations and burden on health systems	24 (13)	26 (15)
Food insecurity and micronutrient deficiencies	31 (17)	30 (17)
Lyme disease		
Habitat expansion	8 (4)	20 (11)
Increased prevalence outside traditional tick season	31 (17)	37 (21)

Abbreviations: AY22, academic year 2021-2022; AY23, academic year 2022-2023; UHI, urban heat islands.
[a]Multiple themes could be counted in each response.

Table 4. Thematic and Content Analysis of Student Evaluation Survey Comments for AY22 and AY23 on Strengths and Suggestions for Improvement, With Examples

Theme	AY22 No.	AY23 No.	Example
Strengths			
Small-group discussion and/or interactive mapping activity	15	16	"The graphs and maps were something I thought were great in explaining this topic, it allowed me a tangible way to see the impacts of climate change and really made it easy to follow along."
Didactic lectures	13	10	"Very well-organized lecture. The content is very relevant, and the lecturers gave us some solutions to combat climate change in relation to the healthcare industry."
Suggestions for improvement			
Time allotted for discussion/ case	7	7	"Less time could be allotted for the small group activities."
Expand discussion on how to advocate for patients and the ways climate change will impact their health	4	2	"Bullet points/text for advocacy points would be very helpful—it felt like a very brief section at the end."
Make didactics more interactive	1	1	"Having more participation in the didactic may be more engaging for the class (e.g., using audience response system)."
Lectures should be more evidence-based	1	1	"More cohort studies of patients affected by climate change with statistically significant information."
Have a small-group facilitator	2	0	"I think having a facilitator in the small group sessions would have made the individual exercise run more smoothly."

Abbreviation: AY22, academic year 2021-2022; AY23, academic year 2022-2023.

Copyright © 2024 Ribeiro et al. This is an open-access publication distributed under the terms of the Creative Commons Attribution license.

content analysis of the quiz responses showed increased awareness of the importance of climate-aware clinical care for improving patient outcomes. Student feedback highlighted the interactive small-group activities (maps and cases) as a strength.

Interestingly, we found that presurvey self-assessment with respect to the Educational Objectives was higher for AY23 than AY22. This may have been related to increasing urgency, awareness, and advocacy among medical students around the health effects of climate change over time in the setting of the increasing frequency of climate events or to improved engagement with the return to in-person instruction. Follow-up research would be needed to fully assess this finding and the document trends over time.

A strength of the seminar was the prioritization of exploring environmental health disparities in the local context. Knowledge of how vulnerable populations bear disproportionate burdens from climate-related health stressors can result in more effective physician advocacy and public health policies. Furthermore, the US health care sector is responsible for 9%-10% of all greenhouse gas emissions in the United States.[17] Failure to acknowledge and address these issues at a systemic level can result in hospitals continuing to impose environmental harm on the communities they serve.[18] The mapping activity and case-based learning as presented in the Appendices are meant to be customized to any geographic region, thereby increasing awareness of environmental injustices and how they contribute to present-day inequities and climate-related illnesses in local communities.

An additional strength of the curriculum design approach was the collaboration of a student, a resident, and faculty. The peer-to-peer and near-peer approach proved successful in ensuring that the topics and methods chosen were relevant, digestible, and interactive. The interactive components also allowed for practical application of adaptation and mitigation strategies at the individual, community, and structural levels. For example, the mapping exercises highlighted how publicly available information could help inform management plans and health policy; the case-based exercises emphasized individual and structural-level interventions that could aid in the health care response to climate change events. Based on student feedback, future offerings will reduce the time spent on the small-group activities from 20 minutes to 15 minutes each (as reflected in Appendix A). As demonstrated here, this interactive seminar was feasible and had similar outcomes both virtually and in-person. It can be tailored to the needs of other institutions looking to provide a similar educational session.

As a form of assessment, the open-content quiz was designed to have learners reengage with the content and reflect on what they had learned. The high scores demonstrate the pitfall of an open-content format, where students can simply look up the answers to multiple-choice questions. The more reflective components of the quiz, the short-answer essay questions, had long been integrated into the HESJ curriculum at our institution at the suggestion of students, who felt that this question format demonstrated more meaningfully how they had learned from and engaged with the content. However, the short-answer essay question responses required significant time investment on the part of educators to thoughtfully review and grade, an important consideration for others seeking to implement a similar seminar.

A major limitation of the data analysis is the use of pre- and postsurvey instruments asking students to self-assess knowledge and skills on a 5-point Likert scale, which can be challenging to translate into educational impact and influence on behavior and skills. There is also a need for long-term outcomes, as our analysis assesses only short-term changes in self-assessed knowledge and skills. The analysis would be strengthened by reassessment once students have been immersed in their clinical clerkships.

This work is not comprehensive, but a single 2.5-hour introductory session. Additional work will be needed to build on this content, particularly during the clinical years. A series of climate change sessions spaced out across the 4 years of medical school would allow for more in-depth instruction. As demonstrated by student feedback, building on this content can highlight student and physician engagement in advocacy at multiple levels—including hospital administration, community leaders, and policymakers—focused on mitigation and adaptation. Furthermore, extending instruction to trainees and attending physicians across the institution to respond to rapidly evolving climate events will be essential for providing evidence-based care with a climate justice lens to the communities we serve.

Appendices

A. Didactic Lectures.pptx

B. Small-Group Student Handouts Example.docx

Copyright © 2024 Ribeiro et al. This is an open-access publication distributed under the terms of the Creative Commons Attribution license.

C. Small-Group Debriefs Example.pptx

D. Pre- and Postseminar Surveys.docx

E. Quiz.docx

All appendices are peer reviewed as integral parts of the Original Publication.

Victoria Ribeiro: Fourth-Year Medical Student, Rutgers New Jersey Medical School

Evan Grossi, MD: Chief Resident, Department of Emergency Medicine, Rutgers New Jersey Medical School

Yaxel Levin-Carrion: Second-Year Medical Student, Rutgers New Jersey Medical School

Novneet Sahu, MD: Associate Professor, Departments of Family and Emergency Medicine, Rutgers New Jersey Medical School

Michelle DallaPiazza, MD: Associate Professor, Division of Infectious Diseases, Department of Medicine, Rutgers New Jersey Medical School; ORCID: https://orcid.org/0000-0003-3262-2368

Disclosures
None to report.

Funding/Support
None to report.

Ethical Approval
The Rutgers electronic Institutional Review Board (eIRB) approved this project.

References

1. Climate change and health. World Health Organization. October 30, 2021. Accessed November 10, 2021. https://www.who.int/news-room/fact-sheets/detail/climate-change-and-health
2. Global climate change and human health H-135.938. American Medical Association. 2019. Updated 2022. Accessed March 5, 2024. https://policysearch.ama-assn.org/policyfinder/detail/climate%20change?uri=%2FAMADoc%2FHOD.xml-0-309.xml
3. Hampshire K, Ndovu A, Bhambhvani H, Iverson N. Perspectives on climate change in medical school curricula—a survey of U.S. medical students. *J Clim Chang Health*. 2021;4:100033. https://doi.org/10.1016/j.joclim.2021.100033
4. Marill MC. Pressured by students, medical schools grapple with climate change. *Health Aff (Millwood)*. 2020;39(12):2050-2055. https://doi.org/10.1377/hlthaff.2020.01948
5. Pre-clerkship electives. Warren Alpert Medical School. Accessed March 5, 2024. https://education.med.brown.edu/md-curriculum/pre-clerkship/pre-clerkship-electives
6. Kligler B, Pinto Zipp G, Rocchetti C, Secic M, Ihde ES. The impact of integrating environmental health into medical school curricula: a survey-based study. *BMC Med Educ*. 2021;21:40. https://doi.org/10.1186/s12909-020-02458-x
7. Sarkar A, Appelbaum NP, Asaithambi R, et al. Climate change as a social determinant of health: an interactive case-based learning activity. *MedEdPORTAL*. 2023;19:11332. https://doi.org/10.15766/mep_2374-8265.11332
8. Climate medicine: EMED 8010. University of Colorado Section of Wilderness and Environmental Medicine. Accessed March 5, 2024. https://www.coloradowm.org/courses/med-student-electives/4th-year-medical-student-elective-climate-medicine-emed-8010/
9. Climate crisis and clinical medicine virtual elective for medical students. Emory. April 30, 2020. Accessed March 5, 2024. https://sustainability.emory.edu/climate-change-elective-for-medical-students-virtual-classes-and-recordings-in-spring-2020/
10. The health effects of climate change. Harvard University. Accessed March 5, 2024. https://pll.harvard.edu/course/health-effects-climate-change?delta=0
11. Virtual Global Climate Change Seminar Series: fall 2020. Weill Cornell Medicine International Medical Student Education. Accessed March 5, 2024. https://international.weill.cornell.edu/whats-happening/events/virtual-global-climate-change-seminar-series
12. Ramkumar J, Rosencranz H, Herzog L. Asthma exacerbation triggered by wildfire: a standardized patient case to integrate climate change into medical curricula. *MedEdPORTAL*. 2021;17:11063. https://doi.org/10.15766/mep_2374-8265.11063
13. Edmunds S, Brown G. Effective small group learning: AMEE Guide no. 48. *Med Teach*. 2010;32(9):715-726. https://doi.org/10.3109/0142159X.2010.505454
14. DallaPiazza M, Ayyala MS, Soto-Greene ML. Empowering future physicians to advocate for health equity: a blueprint for a longitudinal thread in undergraduate medical education. *Med Teach*. 2020;42(7):806-812. https://doi.org/10.1080/0142159X.2020.1737322
15. Salas RN. Environmental racism and climate change—missed diagnoses. *N Engl J Med*. 2021;385(11):967-969. https://doi.org/10.1056/NEJMp2109160
16. Braun V, Clarke V. Using thematic analysis in psychology. *Qual Res Psychol*. 2006;3(2):77-101. https://doi.org/10.1191/1478088706qp063oa

Copyright © 2024 Ribeiro et al. This is an open-access publication distributed under the terms of the Creative Commons Attribution license.

17. Eckelman MJ, Sherman J. Environmental impacts of the U.S. health care system and effects on public health. *PLoS One*. 2016;11(6):e0157014. https://doi.org/10.1371/journal.pone.0157014

18. Eckelman MJ, Huang K, Lagasse R, Senay E, Dubrow R, Sherman JD. Health care pollution and public health damage in the United States: an update. *Health Aff (Millwood)*. 2020;39(12):2071-2079. https://doi.org/10.1377/hlthaff.2020.01247

Received: August 24, 2023
Accepted: January 24, 2024
Published: April 16, 2024

Copyright © 2024 Ribeiro et al. This is an open-access publication distributed under the terms of the Creative Commons Attribution license.
